# ChIP-exo signal associated with DNA-binding motifs provides insight into the genomic binding of the glucocorticoid receptor and cooperating transcription factors

**DOI:** 10.1101/gr.185157.114

**Published:** 2015-06

**Authors:** Stephan R. Starick, Jonas Ibn-Salem, Marcel Jurk, Céline Hernandez, Michael I. Love, Ho-Ryun Chung, Martin Vingron, Morgane Thomas-Chollier, Sebastiaan H. Meijsing

**Affiliations:** 1 Department of Computational Molecular Biology, Max Planck Institute for Molecular Genetics, 14195 Berlin, Germany;; 2 Institut de Biologie de l'Ecole Normale Supérieure, Institut National de la Santé et de la Recherche Médicale, U1024, Centre National de la Recherche Scientifique, Unité Mixte de Recherche 8197, F-75005 Paris, France

## Abstract

The classical DNA recognition sequence of the glucocorticoid receptor (GR) appears to be present at only a fraction of bound genomic regions. To identify sequences responsible for recruitment of this transcription factor (TF) to individual loci, we turned to the high-resolution ChIP-exo approach. We exploited this signal by determining footprint profiles of TF binding at single-base-pair resolution using ExoProfiler, a computational pipeline based on DNA binding motifs. When applied to our GR and the few available public ChIP-exo data sets, we find that ChIP-exo footprints are protein- and recognition sequence-specific signatures of genomic TF association. Furthermore, we show that ChIP-exo captures information about TFs other than the one directly targeted by the antibody in the ChIP procedure. Consequently, the shape of the ChIP-exo footprint can be used to discriminate between direct and indirect (tethering to other DNA-bound proteins) DNA association of GR. Together, our findings indicate that the absence of classical recognition sequences can be explained by direct GR binding to a broader spectrum of sequences than previously known, either as a homodimer or as a heterodimer binding together with a member of the ETS or TEAD families of TFs, or alternatively by indirect recruitment via FOX or STAT proteins. ChIP-exo footprints also bring structural insights and locate DNA:protein cross-link points that are compatible with crystal structures of the studied TFs. Overall, our generically applicable footprint-based approach uncovers new structural and functional insights into the diverse ways of genomic cooperation and association of TFs.

Transcriptional regulatory factors (TFs) control where, when, and at which level a gene is expressed by binding to specific regulatory sequences associated with their target genes. For example, upon hormone binding, the glucocorticoid receptor (GR) associates with GR binding sequences (GBSs) that consist of inverted repeats of hexameric half-sites separated by a 3 base pair (bp) spacer (see sequence logo, Fig. 1C). The recognition sequences of TFs are, however, insufficient to explain their genomic binding profile as these are typically ubiquitously found in the genome and only a cell-type-specific subset is bound (John et al. 2011). For GR, this bound subset localizes predominantly to preexisting loci of accessible chromatin and accordingly, sequence motifs for factors involved in keeping or making chromatin accessible, such as JUN and FOXA1, are overrepresented at these loci (Biddie et al. 2011; John et al. 2011; Reddy et al. 2012).

**Figure 1.**
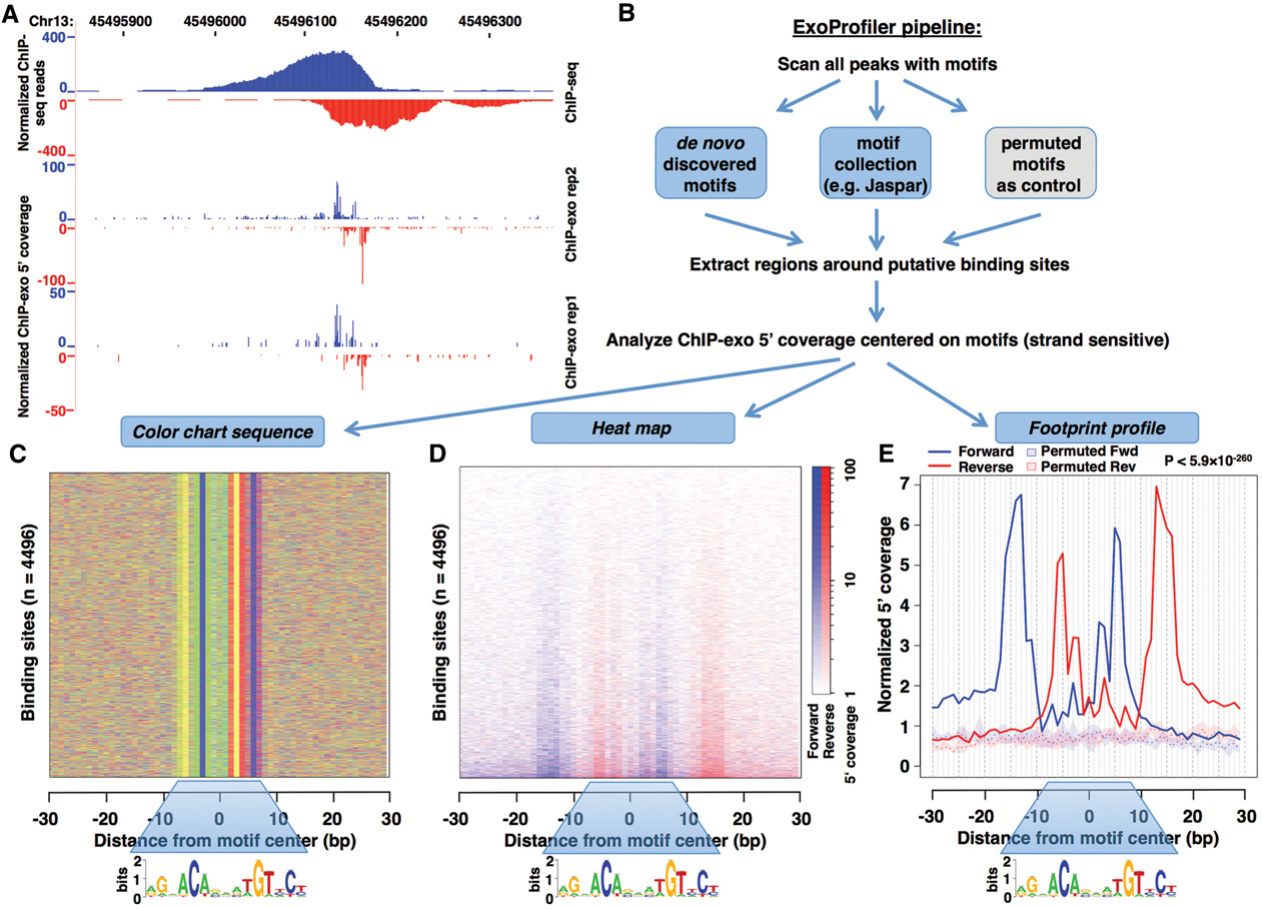
Comparison between ChIP-seq and ChIP-exo signal and a flowchart of the ExoProfiler tool. (*A*) GR binding coverage at the *NUFIP1* locus is shown as a UCSC Genome Browser screenshot for IMR90 ChIP-seq data (*top*) and two ChIP-exo replicates (*bottom*). (*B*) Schematic flowchart of the ExoProfiler pipeline. The pipeline takes as input peak regions and ChIP-exo signal (mapped reads). Peak regions are scanned with motifs to find putative TFBSs and to define short regions around them; the example shown here is for a GBS motif (JASPAR MA0113.2). The 5′ ChIP-exo coverage is calculated in a strand-specific way within these regions. As output, ExoProfiler produces plots, including a color chart summarizing the sequence of motif matches (*C*), a heatmap displaying the 5′ ChIP-exo coverage (*D*), and a footprint profile recapitulating the 5′coverage for all short regions (*E*). As a control, this plot displays the 5′ coverage for regions matching permutated motifs. The permutations are summarized by the median (dotted line) and the interquartile range (shaded area).

Recruitment of GR is not exclusively achieved by GBSs, which adds another level of complexity in understanding its genomic binding profile. For example, the activity of GR at the so-called composite response elements critically depends on the presence of both a GBS (or GBS half-site) and a recognition sequence for another TF that “partners” with GR at such elements (for review, see Ratman et al. 2013). Furthermore, GR can be recruited through protein:protein tethering, for example, via STAT3 (Langlais et al. 2012), JUN (Schule et al. 1990; Rogatsky et al. 2001), and the NFKB complex (Ray and Prefontaine 1994; Luecke and Yamamoto 2005). In addition, several studies have proposed alternative recognition sequences that can be directly recognized by GR (Drouin et al. 1993; Surjit et al. 2011). Consistent with a broader spectrum of sequences that can recruit GR, only a subset of bound genomic loci appear to encode a canonical GBS (John et al. 2011; Siersbaek et al. 2014). Similarly, the analysis of the genomic binding profile of a broad panel of TFs has revealed that many genomic regions appear not to have a canonical binding site motif, suggesting that alternative modes of recruitment might be a general principle for TFs (The ENCODE Project Consortium 2012; Yip et al. 2012).

Typically, candidates for TF binding sites (TFBSs) result from computational analyses aimed at identifying overrepresented sequence motifs within bound regions. However, if and how such predicted sites are indeed bound by the TF of interest is unclear for several reasons: (1) enriched sequences do not necessarily indicate TF binding; (2) they can be directly recognized by the TF studied or be involved in tethering it to the DNA; and (3) they might recruit other TFs that play a role in opening specific genomic regions and thus are not directly involved in providing a physical connection between DNA and the studied TF. Without additional experiments, one cannot differentiate between these scenarios. These limitations are, in part, a consequence of the limited resolution of ChIP-seq that identifies regions rather than the exact location of TF binding. Therefore, we turned to ChIP-exo, which combines ChIP-seq with a subsequent exonuclease step aimed at trimming the ChIPed DNA to the point where the cross-linked protein protects the DNA from further digestion (Rhee and Pugh 2011).

Here, we present a computational pipeline that performs a comprehensive motif-based analysis of the ChIP-exo signal. Our approach uncovers high-resolution footprint profiles of several DNA:bound complexes. Furthermore, we show that the ChIP-exo signal contains information that allows one to discriminate between alternative modes of TF recruitment. We applied our method to novel ChIP-exo data sets to study genomic binding by GR at high resolution in several cell lines. The resulting footprint profiles enabled us to generate new testable hypotheses, ultimately leading to new insights into mechanisms responsible for recruiting GR to specific genomic loci.

## Results

### Genome-wide binding of GR

Processing of GR ChIP-seq data sets resulted in the identification of 47,630 bound loci in IMR90 (primary fetal lung fibroblast), 6,329 in K562 cells (erythromyeloblastoid leukemia cell line), and 41,402 in U2OS cells (osteosarcoma) (Thomas-Chollier et al. 2013). Subsequent GR motif searches in these GR-bound regions showed a striking difference in the fraction of peaks with an apparent GBS between these cell lines at various *P*-value thresholds (Supplemental Fig. S1a), with <42% of peaks having a high-stringency motif match. One potential explanation is that GR may bind to highly degenerate sequences. Alternatively, other sequences present at GR-bound regions may recruit GR directly or indirectly to the DNA. Because ChIP-seq does not have the resolution to discriminate between these scenarios, we turned to ChIP-exo (Rhee and Pugh 2011) to study the genomic interactions of GR at higher resolution. A comparison of ChIP-seq and ChIP-exo signals at a GR-bound region (Fig. 1A; Supplemental Fig. S2b) illustrates the increased resolution.

### ExoProfiler pipeline

To identify the sequences involved in GR recruitment to the DNA, we developed a computational pipeline named ExoProfiler, which analyzes the ChIP-exo signal around sites matching a given motif (for a complete description, see Methods) (Fig. 1B). In brief, ExoProfiler first scans bound regions (here ChIP-seq peaks) with motifs of interest (here JASPAR and de novo identified motifs) to identify putative TFBSs with a high-scoring motif match. For each motif, the tool calculates the ChIP-exo coverage of the most 5′ base of the sequenced reads on both forward and reverse strands relative to the TFBS center as it marks the boundary of protection from lambda exonuclease digestion provided by cross-linked proteins. As output, ExoProfiler produces several plots (Fig. 1C–E), including a footprint profile displaying the total sum of counts over all sites, which is plotted along with a profile obtained with permuted motifs (Fig. 1E).

### Insights into GR binding from GR ChIP-exo signal

#### Canonical GBS

**Figure 2.**
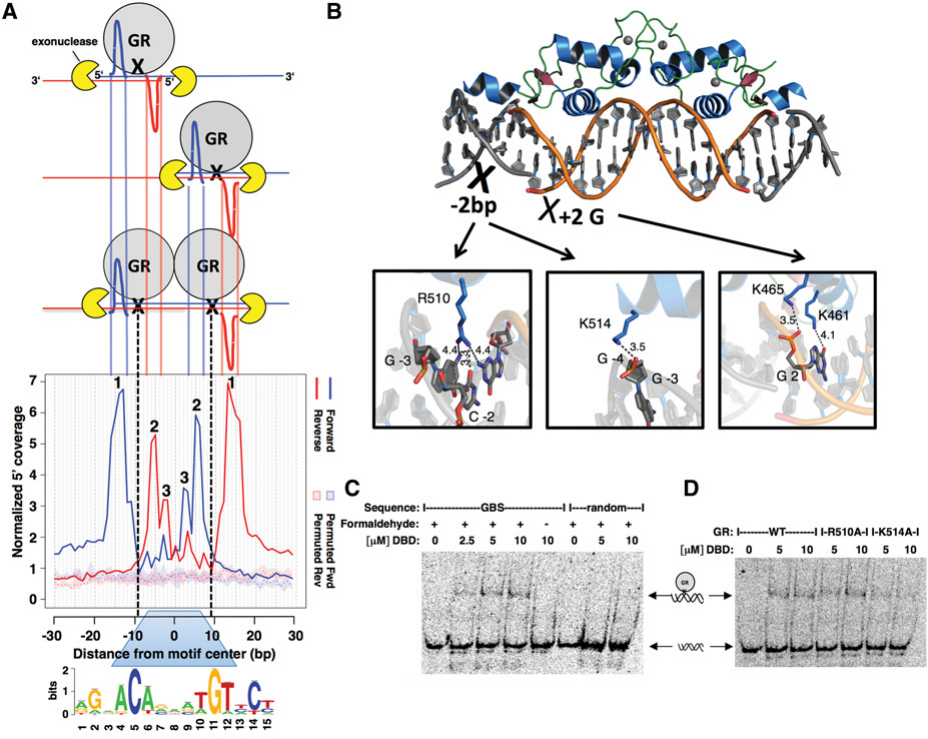
Model explaining the footprint profile for GBSs and identification of one of the GR:DNA cross-linking points. (*A*) Inefficiency of cross-linking and monomeric GR binding results in the cross-linking of either one or both GR monomers. Notably, a population of cells with different cross-link scenarios is analyzed, thus explaining the observed footprint profile. Dashed black lines indicate the hypothesized main DNA:GR cross-linking point (in the *center* of the peak-pair for each monomer). 1, “outer peaks”; 2 and 3, “inner peaks.” (*B*) Contacts mapping to the hypothesized GR:DNA cross-linking region based on the crystal structure of the DNA-binding domain (DBD) of GR (PDB 3G6U). (*C*) Denaturing EMSA identifies cross-linked DNA:GR complexes. Shifted complex is only observed on denaturing gels when DNA:GR DBD (human residues 380–540) complexes are formaldehyde cross-linked. (*D*) Denaturing EMSA showing that the K514A mutation results in decreased DNA:GR DBD cross-linking.

For all cell lines examined, ExoProfiler returned a striking footprint surrounding GBSs (Supplemental Fig. S2). This footprint was specific to GR ChIP-exo experiments, as no such footprint profile was found for GBSs in ChIP-exo data targeting CTCF (Supplemental Fig. S2a) or FOXA1 (data not shown). The GBS-bound GR protects a region of ∼30 bp, which is comparable to the footprint obtained by DNase I footprinting (Payvar et al. 1983). The GR profile (Fig. 2A) can be divided into several peaks: (1) the broader “outer” peaks on the forward and reverse strand (indicated by a “1”) explained by dimeric GR-binding that protects the region surrounding the 15-bp core GBS; and (2) the centrally flanking smaller and sharper “inner” peaks (indicated by a “2”) on the opposite strand arising when only one of the GR monomers is cross-linked to the DNA due to the low efficiency of formaldehyde cross-linking. In addition, it could reflect DNA-dependent GR dimerization (Dahlman-Wright et al. 1990) with one monomer binding prior to the other.

The first ChIP-exo study (Rhee and Pugh 2011) indicated that the DNA:protein cross-link itself protects the 5′ ends from exonuclease digestion and that cleavage occurs ∼5–6 bp upstream of the cross-linking point. The profile observed for the GR motif is compatible with two main cross-linking sites: one for each GR monomer, located between the “outer” and “inner” peak-pair (Fig. 2A, black crosses). The structure of the DNA-binding domain (DBD) of GR (Meijsing et al. 2009) in this region contains several potential DNA:protein cross-linking sites, in particular the contacts made by R510 and K514 from the C-terminal helix 3 (Fig. 2B). Additionally, K465 and K461 contact the G at position 2 of the motif (numbering refers to individual bases within the GBS as shown at the bottom of Fig. 2A) and could explain the smaller additional peak of the inner pair of peaks, 3 bp downstream (indicated by a “3”; Fig. 2A). To test the role of R510 and K514, we compared the in vitro cross-linking efficiency of the wild-type DBD with that of mutant versions, where these residues were changed to alanines that cannot form formaldehyde cross-links (Metz et al. 2004). Although the efficiency of cross-linking was low, we reproducibly observed a shifted complex on our denaturing gels (Fig. 2C) indicative of monomeric GR cross-linking to the DNA. The DNA:GR complex was only observed when the cross-linking step was added and when a GBS was used as DNA, arguing that the assay detected specifically cross-linked GR:GBS complexes. Next, we tested the role of the K514 or R510 residues and found that the R510A mutation did not have a discernable effect on the cross-linking efficiency (Fig. 2D). In contrast, a reduction in efficiency was observed for the K514A mutant (Fig. 2D), indicating that K514 is indeed involved in cross-linking GR to DNA. The reduced cross-linking efficiency for the K514A mutant likely does not reflect the lower DNA-binding affinity of this mutant for two reasons. First, the experiments were done at saturating protein concentrations (Meijsing et al. 2009). Second, reduced cross-linking efficiency was only seen for the K514A mutant, even though both mutants have an approximately twofold reduction in DNA-binding affinity (Meijsing et al. 2009). Notably, for the K514A mutant, cross-linking was reduced but not lost, suggesting that additional GR residues cross-link to the DNA, possibly K465, K461, and additional residues of helix 3, which might explain the broader “outer” peaks.

Together, the footprint profile suggested that, consistent with structural data in vitro, GR binds as a dimer to genomic GBSs in vivo, and indicated that cross-linking occurs, at least in part, by DNA contacts made by helix 3 outside the GBS.

#### GR binding to degenerate GBSs

A possible explanation for the low fraction of ChIP-seq peaks harboring a GBS is that GR binds to highly degenerate sequences. Simply loosening the motif-scanning threshold to find these sequences is nevertheless not informative, as peak and control regions then contain a similar fraction of motif-matching sequences (Supplemental Fig. S1a). In contrast, the distinct footprints obtained by ChIP-exo provides us with the opportunity to test if degenerate sequences are bound by GR. Therefore, we divided the motif matches in IMR90 ChIP-seq peaks into six subsets of increasing *P*-value thresholds before applying ExoProfiler. GR motif matches at less stringent thresholds (*P*-value ≤10^−3^) yielded footprints resembling the GBS footprint in both the shape and position of peaks (Supplemental Fig. S3a). Furthermore, a cut-off (*P*-value ≤5 × 10^−3^) at which most control and ChIP-seq peaks harbor a motif match (86% for IMR90 vs. 83% for control regions) (Supplemental Fig. S1a) still yielded a GBS-like footprint, indicating GR binding to such sequences.

**Figure 3.**
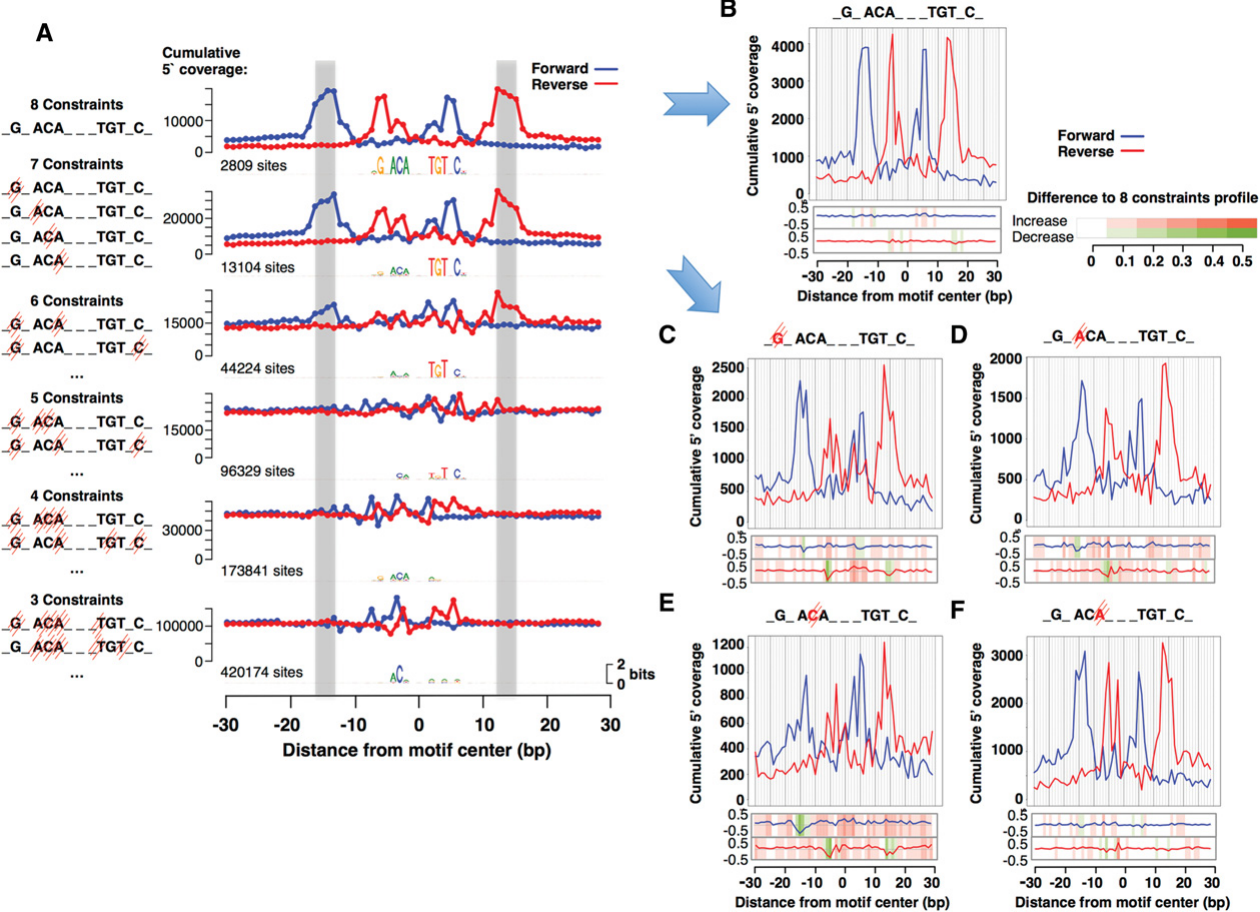
ChIP-exo footprint profiles for different numbers of bases matching the GBS consensus sequence. (*A*) Cumulative footprint profile for sequences with the number of constrained bases matching the GBS consensus (nGnACAnnnTGTnCn) as indicated. Gray area marks the position of the “outer peaks.” (*B*) Footprint profile on a random subsampling of 505 GBS with eight constrained bases. The heatmap *below* indicates the difference between this subsampled profile and the full profile on its *left*. (*C*–*F*) Footprint profiles on a random subsampling of 505 matches of four “mutated” GBS consensuses, all with seven constrained bases. The strike-through base is fixed to be any base except the one matching the consensus preference at that position. The heatmap *below* indicates the difference between this subsampled profile and the full 8-bp constrained profile.

To determine which position(s) of the motif were important for GR binding, we performed in silico “mutations” of positions within the GBS consensus motif and assayed if a footprint was still found for such sequences. Sequences matching all eight bases with the highest information content in the motif (eight constrained positions) resulted in a footprint profile (Fig. 3A). Similarly, when six or seven bases were fixed to match the consensus, we found a footprint profile, but none with five or fewer matching bases (Fig. 3A). The footprint for sequences with six constrained positions further underscores that GR can bind to degenerate sequences in vivo, as such sequences are found frequently in the genome. Regarding the contribution of individual bases on GR's ability to interact with DNA, each combination of sequences with seven bases matching the consensus produced a footprint profile indicative of GR binding (Fig. 3C–F). However, when one of the GR half-sites lacked the preferred C at position 5, the footprint coverage values show the largest decrease on both strands (distance −16 on forward and −5 on reverse) (Fig. 3E), likely reflecting weaker binding of GR to such GBSs. The profile is affected differently depending on which position diverges from the consensus preference (Fig. 3C–F), and changes most at the GBS half-site with the “mutated” base. For example, the absence of the preferred G at position 2 lowers peak 2 (distance −6) (Fig. 3C), whereas the relative intensity of peak 3 (distance −3) increases when position 6 diverges from the preferred A (Fig. 3F). Notably, no footprint profile was observed for sequences that have only one half-site matching the consensus sequence at all important positions, indicating that GR is unable to bind such sequences in this cell line. Together, these results suggest that GR can bind to highly degenerate sequences. However, in contrast to high-scoring motifs within GR peaks that are typically bound, additional inputs are likely needed to specify which of the loosely defined sequences, for which a much smaller fraction is bound (data not shown), are occupied.

Depending on the cell-type examined, different fractions of the ChIP-seq peaks appear to have a GBS sequence (Supplemental Fig. S1a). Especially for K562 cells, fewer GBS-like sequences are found (313 vs. 4496 in IMR90 and 6236 in U2OS) (Supplemental Fig. S2a). This suggests that in K562 cells, GR is either recruited by other sequences or preferably associates with more degenerate GBS sequences. To test the latter possibility, we repeated the analysis of degenerate GBSs for all cell lines. For high-scoring motifs, we found a similar footprint profile for each cell line (Supplemental Fig. S3). This indicates that GR can bind to GBSs in each of these cell lines. Contrary to our expectation and in contrast to U2OS and IMR90 cells, more degenerate GBS sequences failed to produce a footprint profile in K562 cells (Supplemental Fig. S3). Together, this indicates that the low percentage of peaks with a high-scoring GBS motif match in K562 cells is not a consequence of GR binding to highly degenerate sequences.

### ExoProfiler identifies profiles for non-GBS motifs in GR ChIP-exo data

ExoProfiler was systematically applied to all motifs resulting from de novo motif discovery in GR ChIP-seq peaks and motifs from JASPAR. The motifs to study further were selected by ranking on their coverage *P*-value (Supplemental Data 2 for IMR90), choosing a representative motif for groups of related/redundant motifs, and visual inspection of the profile, to ensure coherent peak-pairs with forward reads density upstream of reverse reads density. Interestingly, several non-GBS motifs produced footprint profiles with significantly enriched ChIP-exo coverage. The shape of these profiles is distinct from the one for GBSs, indicating that other proteins are recruited by these sequences or that GR binds such sequences in a distinct manner. The interpretation and functional analysis of several individual footprint profiles and their role in recruiting GR to the genome are discussed below.

#### Combi motif footprint profile

**Figure 4.**
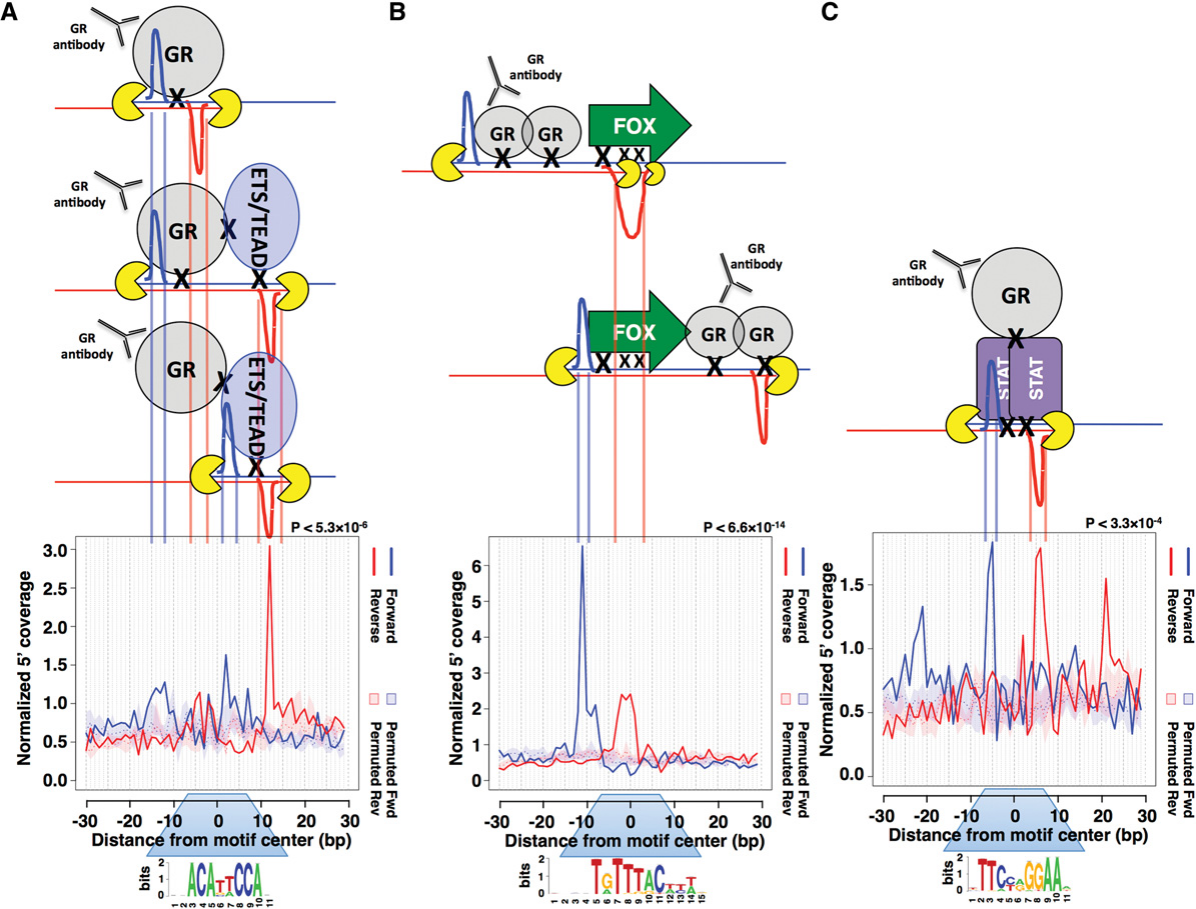
ExoProfiler identifies non-GBS footprint profiles in GR ChIP-exo data from IMR90 cells. Footprint profiles and models explaining these profiles: (*A*) for a de novo identified “combi” motif where monomeric GR binds together with a “partnering” protein from the ETS or TEAD family at a composite binding site; (*B*) for the FOXA1 motif (JASPAR MA0148.3), which could be a consequence of summarizing the reads derived from several loci at which GR and one of the FOX TFs are simultaneously cross-linked (combinatorial mode of binding); and (*C*) for the STAT1 motif (JASPAR MA0137.3) where GR is tethered to the DNA via STAT proteins.

**Figure 5.**
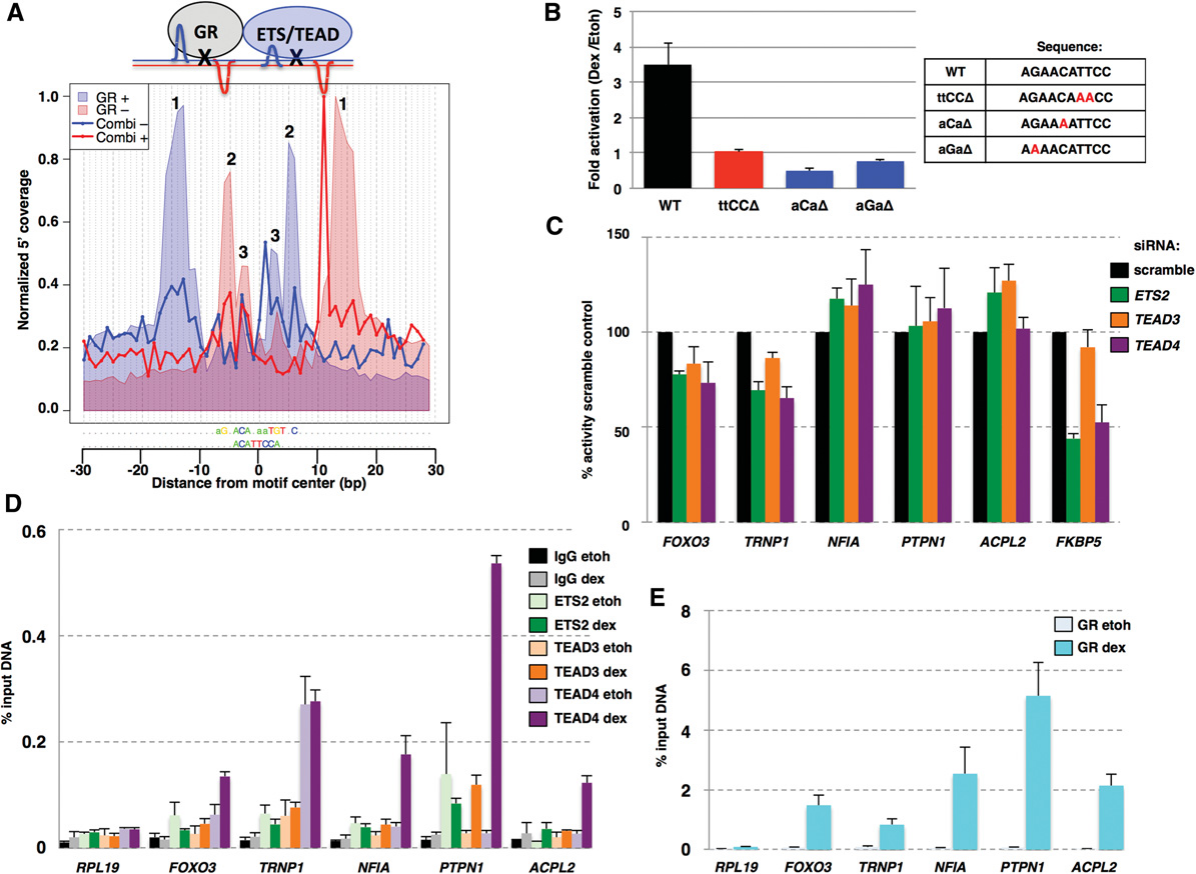
Characterization of the combi motif. (*A*) Alignment of the footprint profiles for the combi and the GBS motifs and a model indicating how this profile can be explained by binding of GR and another “partnering” protein. 1, “outer peaks”; 2 and 3, “inner peaks.” (*B*) Transcriptional activity of luciferase reporters containing a minimal promoter and three copies of the combi motif or mutant versions as indicated. Fold induction ± SEM (*n* ≥ 3) in U2OS cells upon treatment with 1 μM dexamethasone (dex) is shown. (*C*) Effect of siRNA knockdown of genes as indicated on transcriptional induction of GR target genes with nearby combi-motif-containing ChIP-seq peaks. Two days after U2OS cells were transfected with dsiRNAs, cells were treated with EtOH as vehicle control or dex, and percentage induction, relative to the scramble control, ±SEM (*n* ≥ 3) was determined by qPCR. (*D*) ETS2, TEAD3, TEAD 4, nonspecific binding (IgG) and (*E*) GR binding at genomic loci harboring a combi motif. ChIP experiments were performed for U2OS cells treated with EtOH as vehicle control or dex, and binding was examined for GR-bound regions with a combi motif or an unbound region (*RPL19*) as a control. The percentage of input immunoprecipitated ±SEM (*n* = 3) is shown.

A de novo motif identified in GR-bound regions in IMR90 cells resembles the recognition sequence for TEAD/TEF TFs (Fig. 4A; Wasserman and Fickett 1998). Several other studies have found enrichment of this motif at GR-bound regions (Biddie et al. 2011; Polman et al. 2012), but its role in recruiting GR to the genome in unclear. The TEAD motif resembles a GBS; however, instead of having two half-sites separated by a 3-bp spacer, it only contains a single half-site followed by TTCC. Aligning the footprints for the TEAD and GBS motifs on the half-site revealed an overlap in both the shape, position, and relative intensities of the inner and outer peaks (Fig. 5A), suggesting that a GR monomer is bound at this half-site for both motifs. In contrast, the second peak-pair for the TEAD motif looked different from the profile for GBSs, suggesting the binding of another protein. We therefore termed this motif “combi” as it appears to reflect a composite binding site where a GR monomer binds together with another protein. Consistent with monomeric GR binding, electrophoretic mobility shift assays (EMSAs) showed that the DBD of GR binds as a monomer to the combi site regardless of whether the additional TTCC flanked the GR half-site or not (Supplemental Fig. S4a).

We next studied the combi motif functionally, by constructing reporters containing genomic regions bearing combi binding sites. For all reporters tested, we found a GR-dependent activation that was reduced when we mutated the combi sequence at key positions (Supplemental Fig. S4b). We next tested if the combi sequence alone is sufficient to mediate GR-dependent transcriptional regulation by inserting three copies upstream of a minimal promoter driving the expression of a luciferase reporter gene. This reporter showed a hormone-dependent activation that was lost when positions in the GR half-site or the TTCC sequence were mutated, in both IMR90 (Supplemental Fig. S4c) and U2OS cells (Fig. 5B). A search for the TTCC motif in the JASPAR database identified several candidate binding TFs, including TEAD proteins, components of the NFKB complex, and members of the ETS family of TFs, for example, ETS1 and ETS2, that have been shown to physically interact with GR (Mullick et al. 2001). Structural alignments for ELK1 (ETS family), ETS1, and TEAD1 indicated that these factors are capable of cobinding with GR (Supplemental Fig. S4d); however, this was also true for other candidate proteins (STAT and NFKB components) we aligned (Supplemental Data 3). To assess the role of candidate factors in GR-dependent activation from the combi motif, we prioritized proteins whose complete recognition sequence is present in the combi motif (true for members of the ETS and TEAD family of TFs), and for ETS members we chose to test proteins that interact with GR (ETS1 and ETS2). Next, we knocked down their expression using dsiRNAs (Supplemental Fig. S4e) and found that knockdown of *ETS2*, *TEAD3* and *TEAD4* reduced activation from the combi motif by ∼50%, whereas knockdown of *ETS1*, *ELK1*, *TEAD1*, and *TEAD2* showed little effect (Supplemental Fig. S4f). As control, knockdown of *ETS2*, *TEAD3*, or *TEAD4* showed little to no effect on a luciferase reporter containing three GBS copies (Supplemental Fig. S4f; Meijsing et al. 2009), indicating that these factors are specifically involved in GR-dependent activation at the combi motif. Furthermore, knockdown of *ETS2* and *TEAD4*, and to a lesser degree *TEAD3*, resulted in a reduced GR-dependent activation of target genes with nearby combi-motif containing ChIP-seq peaks (*FOXO3*, *TRNP1*, and *FKBP5*) (Fig. 5C).

In addition, ChIP experiments targeting ETS2, TEAD3, or TEAD4 showed that they are bound at several GR-bound regions containing a combi motif (Fig. 5D,E; same regions as those tested with luciferase reporters Supplemental Fig. S4b). For example, all three factors appear to bind at the *PTPN1* locus, with ETS2 binding independently of hormone treatment, whereas TEAD3 and TEAD4 binding is only observed upon hormone treatment.

Collectively, our footprint, functional, and structural studies indicate that GR-dependent activation at sites matching the combi motif is a consequence of binding of a GR monomer in conjunction with a partnering protein (possibly ETS2, TEAD3, or TEAD4, but we cannot exclude other proteins).

#### FOX motif footprint profile

Motif discovery in IMR90 cells also revealed motifs bound by forkhead box (FOX) TFs sharing a similar DBD. For these motifs, ExoProfiler uncovered footprint profiles with significantly enriched ChIP-exo coverage that are markedly different from the one observed for GBSs (Fig. 4B). The distinct profile suggests that a protein other than GR binds and is consistent with this; EMSAs showed no increased affinity of the GR DBD for the FOX consensus sequence when compared to randomized control sequences (Supplemental Fig. S5a).

The most obvious candidates for binding are members of the FOX family of TFs. Because no structure is available for FOXA1, we examined the structure of a homologous protein, FOXK1 (Tsai et al. 2006), and found a conserved lysine residue, K328, in the wing domain that maps to the cross-linking point, which is located in between the forward and reverse peaks (Supplemental Fig. S5b). The sharp peak on the forward strand indicates that K328 cross-links very efficiently, whereas two additional lysine contacts, K300 and K318, might only cross-link in a fraction of cases, resulting in alternative protection sites and consequently a broader peak on the reverse strand.

**Figure 6.**
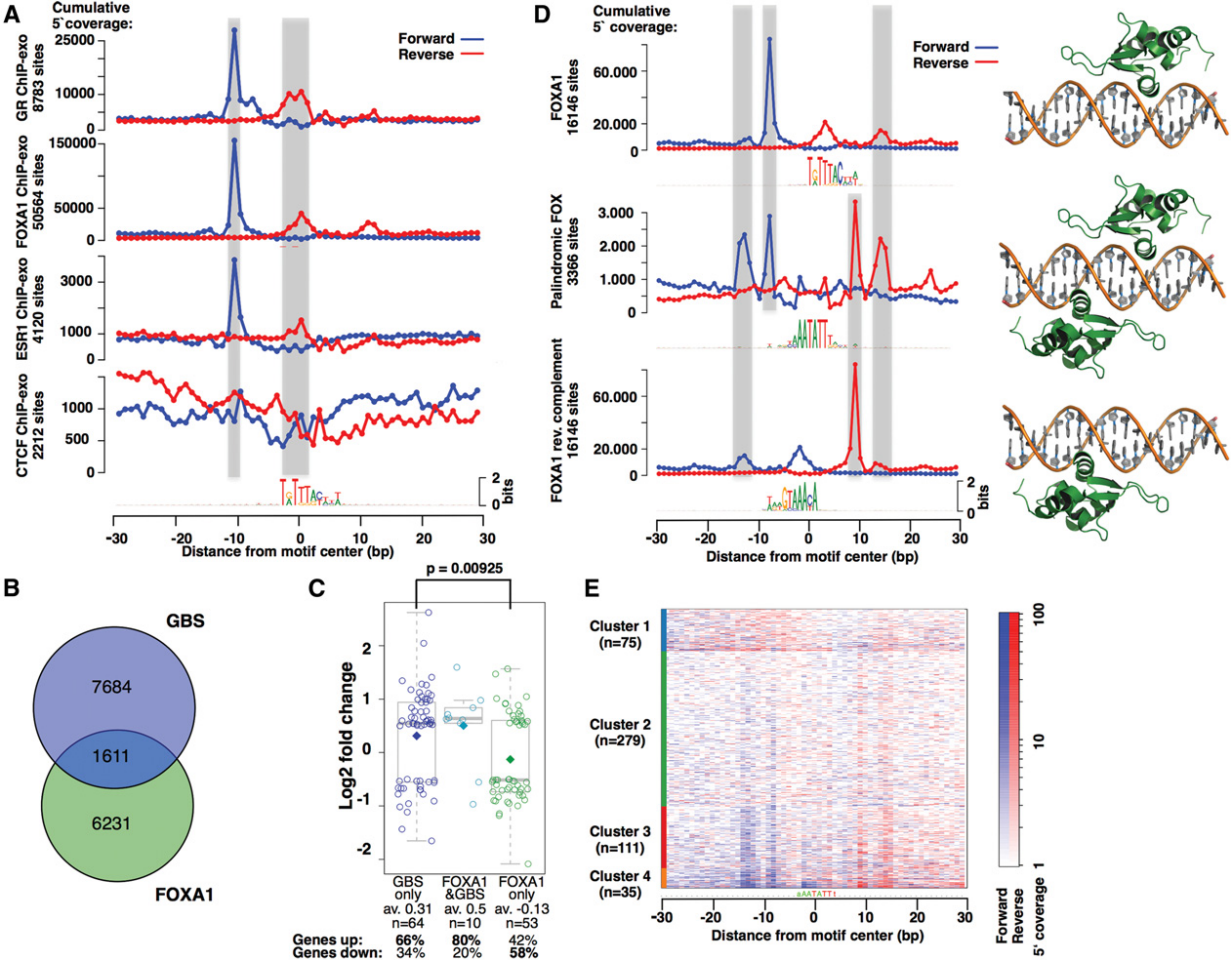
FOX motif analysis. (*A*) Footprint profiles for the FOXA1 motif in different ChIP-exo experiments: GR in IMR90, FOXA1 in MCF7, ESR1 in MCF7, and CTCF in HeLa cells. (*B*) Venn diagram showing the number of GR-bound regions with a FOXA1 and/or GBS motif in IMR90 cells. (*C*) Boxplot of log-fold change for genes that are differentially expressed upon treatment for 4 h with 1 μM dexamethasone (log-fold change ≤ −0.5 or ≥0.5). Genes with ChIP-seq peaks in the region ±20 kb around the TSS with only a FOXA1 motif (*P* < 0.0001) are marked in green; genes with peaks with only a GBS (*P* < 0.0001) motif in dark blue; and genes with peaks with sequences matching both motifs in turquoise. *Center* lines show the medians; diamonds show the mean; and box limits indicate the 25th and 75th percentiles as determined by R software; whiskers extend 1.5 times the interquartile range from the 25th and 75th percentiles. (*D*) FOXA1 ChIP-exo profile for the palindromic FOX motif (*middle*) align with those for the FOXA1 motif (*top*) and its reverse complement (*bottom*). Structural alignment indicates that the palindromic FOXA1 binding site can be simultaneously bound by two FOXK1 proteins (PDB 2C6Y), a close homolog of FOXA1. (*E*) K-means clustering of the 500 most occupied palindromic FOXA1 binding sites.

To test the hypothesis that the observed footprint profile reflects binding of a member of the FOX family of TFs, we applied ExoProfiler to FOXA1 ChIP-exo data (Serandour et al. 2013). Interestingly, both our GR-based and FOXA1-based ChIP-exo footprints showed the distinct sharp peak on the forward strand exactly 8 bp upstream of the motif and the broader peak on the opposite strand (Fig. 6A). In conclusion, although we have not directly identified the protein responsible for the FOX footprint, the structural clues and striking resemblance to the FOXA1 footprint argue that binding of a member of the FOX family of TFs is responsible for the FOX footprint observed in our study.

Notably, the FOX footprint is observed even though the ChIP was performed with a GR-specific antibody. This could happen when the associated protein is efficiently cross-linked to the DNA near sites of GR binding and consequently coprecipitates during the ChIP procedure (Fig. 4B; Supplemental Fig. S6a). We studied the functional connection between FOX binding sites and GR-dependent binding and regulation by constructing several reporters containing genomic regions bearing both FOX and GR binding sites. For these GR-activated reporters, mutation of the FOX sequence at key positions resulted in reduced GR-dependent regulation (Supplemental Fig. S6b). Similarly, mutating GBS-like sequences resulted in reduced activity for three out of four reporters (Supplemental Fig. S6b). To identify which of the FOX members cooperates with GR, we knocked down the expression of two FOX transcripts, *FOXL1* and *FOXF1*, which are expressed at high levels in IMR90 cells. However, we failed to see an effect of their knockdown on GR-dependent transcriptional activation, perhaps as a consequence of limited knockdown efficiency. We also performed DNA pull-down assays using nuclear extract from IMR90 cells and identified FOXK1 as an interaction protein, so perhaps another Fox or multiple FOX members cooperate with GR in IMR90 cells. Together, we find a clear connection between GR-dependent transcriptional activation and the presence of a FOX sequence, reflecting a role for a FOX TF in facilitating GR-dependent transcriptional activation from GBSs nearby.

Alternatively, the FOX footprint could reflect FOX-dependent, tethered GR binding. Indeed, many GR ChIP-seq peaks contain a FOXA1 motif match but no GBS match (Fig. 6B). Tethered GR binding has been linked to transcriptional repression (Ratman et al. 2013). We therefore examined the GR target genes associated with ChIP-seq peaks that harbor sequences matching the FOXA1 motif but lacking a GBS motif match. Interestingly, these FOXA1-only genes were, on average, down-regulated upon hormone treatment, whereas genes associated with ChIP-seq peaks that matched a GBS or both a GBS and FOXA1 motif were up-regulated (Fig. 6C).

Together, our data suggest that FOX proteins are cobound at genomic regions of GR binding and can either tether GR to such regions or play a role in facilitating GR binding to GBSs nearby.

#### STAT footprint profile

A distinct footprint profile was also observed for various related sequence motifs for members of the STAT family of TFs that bind as dimers to STAT binding elements (SBEs) consisting of inverted repeats of 4-bp half-sites separated by a 1-bp spacer (Fig. 4C; Langlais et al. 2008). We found that SBEs are enriched at GR ChIP-seq peaks in IMR90 cells and that a large fraction of these peaks appear to have an SBE matching sequence but not a GBS (Supplemental Fig. S7a). There is a well-established functional and physical connection between STAT proteins and GR (for review, see Rogatsky and Ivashkiv 2006). For example, STAT3 can tether GR to the DNA; this type of binding is associated with GR-dependent transcriptional repression (Langlais et al. 2012). Consistent with tethered GR binding, our ChIP experiments targeting STAT3 showed STAT3 binding at several GR-bound regions containing an SBE in both the presence and absence of hormone treatment (Supplemental Fig. S7b). Furthermore, genes associated with GR ChIP-seq peaks containing an SBE but lacking a GBS were, on average, transcriptionally repressed by GR (Supplemental Fig. S7c), whereas genes associated with ChIP-seq peaks containing GBSs but lacking SBEs were, on average, activated (Supplemental Fig. S7c). Together, these findings indicate that ChIP-exo data uncover footprints reflecting tethered GR binding.

### ExoProfiler applied to other ChIP-exo data

As our profile-based analysis with ExoProfiler is generic, we applied it to public ChIP-exo data sets for other TFs. For CTCF, we found a striking footprint for the CTCF consensus motif (Supplemental Fig. S8a), with two cross-linking regions, consistent with published data (Rhee and Pugh 2011). However, in contrast to our GR ChIP-exo data set, no clear additional footprint profiles were found for other sequence motifs. When applied to the FOXA1 ChIP-exo data, in addition to the footprint profile for FOX motifs (Fig. 6A), we identified a distinct footprint profile for a de novo identified motif (Fig. 6D). This motif, which resembles a FOXA1 motif described in another study (Jankowski et al. 2013), aligns with the FOXA1 motif in both forward and reverse orientation, and the footprint profile for this palindromic sequence can be explained by FOXA1 binding in two orientations (Fig. 6D). Clustering of the ChIP-exo coverage at individual sites indicates that the observed footprint profile is not simply a consequence of summarizing the ChIP-exo reads of loci at which FOXA1 binds in one orientation with loci at which it binds in the other orientation. Rather, the footprint profile for this palindromic FOX motif is also found at individual loci (e.g., for cluster 3 and 4, see Fig. 6E; Supplemental Fig. S6d), arguing that FOXA1 binds these sequences in two orientations. Structural alignment of the homologous FOXK1 protein indicated that this motif can be simultaneously bound by two FOX proteins (Fig. 6D; Jankowski et al. 2013). However, since the footprint is derived from many cells, it might also reflect FOXA1 binding in one orientation for some cells and in the opposite orientation for others.

Finally, when applied to ChIP-exo data for ESR1, ExoProfiler identified a footprint profile for the consensus ESR1 binding motif. When aligned and compared to the footprint profile for GR binding at GBSs, we found that the outer peaks overlap in position with those found for GR (Supplemental Fig. S8b). This is not surprising, given that the structure and sequence of the DBDs of GR and ESR1 are related (Supplemental Fig. S8b). In addition, we found a footprint profile for FOX motifs with striking similarity to the profile found in the FOXA1 ChIP-exo data (Fig. 6A), arguing for the binding of a FOX protein.

## Discussion

GR-bound regions are typically enriched for multiple sequence motifs. However, how and if such enriched sequences are involved in TF recruitment is often unclear. For example, nonspecific motifs in ChIP-seq peaks can be found as a result of cross-linking and enrichment of DNA fragments that simply colocalize in the nucleus with directly TF-bound loci (Worsley Hunt and Wasserman 2014). Here, we set out to identify sequences directly involved in the recruitment of GR to individual loci, taking advantage of the increased resolution provided by ChIP-exo.

In contrast to other studies (Rhee and Pugh 2011; Serandour et al. 2013), we found that only part of the ChIP-seq peaks is covered by ChIP-exo signal and vice versa. The additional signal found only by ChIP-exo might reflect the higher sensitivity of this assay (Rhee and Pugh 2011). It could also reflect the typical lack of a control for the ChIP-exo procedure, preventing one from filtering out nonspecific peaks. Accordingly, we found a strong ChIP-exo signal that overlapped with peaks found in the ChIP-seq input control (e.g., hg19 Chr 17: 22019198–22026876) that are thus filtered out when starting with ChIP-seq data. Here, our priority was to identify which sequences are responsible for binding at individual loci, and thus we opted to focus our analysis on GR-bound regions identified by both methods as these are most likely to reflect real binding events.

ChIP-exo is a powerful technique to reveal individual binding sites, but for some data sets, like ours on GR, combinatorial and tethered binding brings a fuzzy ChIP-exo signal. To fully take advantage of this technique, we developed a motif-based approach exploiting the 5′ ChIP-exo coverage. The resulting footprint profiles and associated plots are not a novel concept (Rhee and Pugh 2011). Here however, we systematically tested hundreds of motifs, rather than just the one for the TF of interest, thereby revealing extra information contained in the ChIP-exo data, which can serve to elaborate new testable hypotheses to uncover binding mechanisms. We provide our method as a free and usable open source tool for the community, named ExoProfiler (https://github.com/ComputationalSystemsBiology/ExoProfiler). In principle, DNase I footprinting could give similar information to that provided by the ChIP-exo procedure. However, recent studies have revealed that DNase I footprints, for example, for GR (Sung et al. 2014), reflect sequence bias of the nuclease. In contrast, the ChIP-exo footprints we observe here are specific to the protein precipitated (Supplemental Fig. S2a).

In addition to information regarding sequences responsible for recruiting TFs to specific loci, the footprint profiles derived from ChIP-exo data yield in vivo structural insights. For example, the proposed DNA:protein cross-link points for GR, based on the footprint profile, align with several DNA contacts found in the crystal structure. Similarly, the footprint profile for the FOXA1 ChIP-exo data is compatible with the crystal structure of DNA-bound FOX proteins (Tsai et al. 2006). Moreover, when applied to a de novo identified palindromic FOX binding motif, our analysis indicated that FOXA1 binds such sequences in two orientations.

Several studies have indicated that in addition to its role in guiding proteins to defined genomic loci, DNA can act as an allosteric ligand that influences the structure of associated proteins (Meijsing et al. 2009; Zhang et al. 2011; Watson et al. 2013). For example, changing the spacer sequence or the sequence of individual half-sites of GBSs influences the structure of the DBD of GR. We reasoned that these structural changes could have an effect on how the DBD cross-links to DNA and consequently influence the footprint profile. To test this, we compared the footprint profile for GBS-matches with a spacer bearing AAA sequence (which yields a narrow spacer) with those with a GGG spacer (wide spacer) (Meijsing et al. 2009). This comparison showed subtle changes in the position and relative signal intensities of individual positions of the peaks (Supplemental Fig. S8c). This suggests the GR DBD cross-links DNA at different positions, depending on the sequence of the spacer. Importantly, based on the footprint profile, the cross-linking point does not map to the spacer region, arguing that the changes in the footprint profile are not a simple consequence of sequence-specific, cross-linking efficiencies. Subtle changes in the footprint profile are also observed for GBSs with different half-site sequences (Fig. 3B–F). Interestingly, these footprint differences are also observed for the invariable second half-site, although it matches the consensus sequence at all key positions. These differences at the invariable half-site corroborate NMR data, indicating allosteric communication between dimer partners (Watson et al. 2013).

### New insights into GR binding

Distinct footprint profiles were also observed for degenerate GBS-like sequences, even though they are found at roughly the same frequency at both GR-bound and unbound regions. Consequently, conventional motif-scanning approaches with a stringent threshold likely underestimate the fraction of peaks where binding is a consequence of GR binding to GBS-like sequences. Surprisingly, degenerate GBS-like sequences failed to produce a clear footprint profile in K562, the cell line with the lowest fraction of GR ChIP-seq peaks harboring a high-scoring GBS (Supplemental Figs. S1a, S3b). This suggests that other non-GBS-like sequences might be responsible for directing GR to the chromatin in K562 cells. Accordingly, we found a footprint profile in K562 cells for several related GATA recognition sequences (Supplemental Fig. S7d), which are specifically enriched in K562 cells (Supplemental Fig. S7e) and might play a role in tethering GR to the DNA. Finally, our analysis uncovered a novel functional GR binding sequence we called combi motif, at which a GR monomer partners with other TFs to activate transcription.

### New insights into GR cofactors

A major finding of our study is that ChIP-exo uncovers signal from more bound proteins than the one targeted by the antibody used for precipitation. This shows that motifs, such as FOX, are not just enriched at GR-bound regions but are simultaneously occupied, perhaps reflecting that FOXA1-induced changes in the chromatin structure are a prerequisite for GR binding (Belikov et al. 2009). The connection between steroid hormone receptors and FOX proteins is perhaps best characterized for ESR1. For example, FOXA1 is required for almost all genomic ESR1-binding events, and accordingly, the introduction of FOXA1 in cells expressing low levels of this protein renders ESR1 capable of binding to many previously unbound loci (Hurtado et al. 2011). Part of this might be explained by FOXA1's role in establishing accessible chromatin (Cirillo et al. 2002). Our FOXA1 motif footprint profile for ESR1 ChIP-exo data provides an alternative explanation, in which ESR1 association with the DNA is mediated by its interaction with FOXA1. This tethered binding might in turn increase the likelihood of ESR1 binding to canonical binding sites nearby as ESR1-bound regions typically contain both a FOXA1 and an ESR1 motif match (Carroll et al. 2005). A similar mechanism might be in play for GR, although in contrast to ESR1, GR binding is redistributed rather than lost when FOXA1 levels are depleted (Sahu et al. 2013). In support of FOX-dependent tethered binding, GR physically interacts with FOXA2 (Sugiyama et al. 1998). Furthermore, the DBD of the androgen receptor, which is almost identical to the DBD of GR, interacts with FOXA1 (Gao et al. 2003). Tethered binding has been linked to transcriptional repression (Ratman et al. 2013), and accordingly, we found that genes with nearby GR-bound peaks matching a FOXA1 motif but lacking a GBS match were, on average, transcriptionally repressed. However, GR failed to regulate a reporter with multiple FOX binding sites (Supplemental Fig. S6c), arguing that the interplay between FOX proteins and GR is complex and that further studies are needed to understand the exact nature of their interaction.

We also explored the footprint profiles for other sequence motifs linked to GR binding. For sequences matching negative response elements (nGREs) that might be bound by GR as a monomer (Hudson et al. 2013), we did not find a corresponding footprint profile in our ChIP-exo data for IMR90 cells. JUN is a major partner required for productive GR–chromatin interactions, likely by making chromatin accessible for GR binding (Biddie et al. 2011) or by tethering GR to the DNA (Schule et al. 1990; Rogatsky et al. 2001). In spite of JUN motif enrichment at GR-bound regions, we failed to see a footprint profile for the JUN motif in any of the cell lines, arguing that its key role might be providing chromatin accessibility, rather than tethering GR to the genome in the cell lines examined.

### Footprint profiles as puzzles to reconstruct enhanceosomes

The power of our relatively simple approach is to reveal key information encoded in the ChIP-exo data: the characteristic footprint profiles that vary in peak shape, number, and relative position of peak-pairs depending on the bound protein. Indeed, the footprint profiles for CTCF, FOXA1, and GR are distinct with characteristic peak patterns, whereas for the related proteins ESR1 and GR, they look similar (Supplemental Fig. S8b). Here we show that a profile-based analysis can uncover structural and functional clues about the interaction and cooperative nature of genomic TF binding. Furthermore, our data suggest that the footprint-based analysis (Supplemental Fig. S9) allows one to distinguish among direct DNA binding, tethered binding, and cooperative binding with other proteins, as illustrated for the combi motif. Our approach does not discern which protein partners with GR, and its identification requires additional experiments because we only have footprint profiles for a few TFs. However, we envision that in the future the availability of ChIP-exo footprints for many TFs will allow one to align protein-specific footprints for a detailed reconstruction of the different modes by which TFs assemble either alone or in cooperation with partnering proteins at regulatory regions.

## Methods

### Cell lines, transient transfections, gene expression profiling, dsiRNA

IMR90 (ATCC: CCL-186), K562 (ATCC: CCL243), and U2OS cells were cultured as recommended by the provider. Transient transfections of plasmids and dsiRNA and a list of primers and dsiRNAs used are described in detail in the Supplemental Experimental Procedures. The effect of hormone treatment on gene expression in IMR90 cells (dexamethasone, 1 µM for 4 h) was analyzed using HumanHT-12 v3 BeadChip (Illumina).

### ChIP, ChIP-seq, and ChIP-exo

ChIP and ChIP-seq assays for cells treated with 0.1% ethanol vehicle or 1 M dexamethasone for 1 h were essentially done as previously described (Meijsing et al. 2009). For ChIP-exo experiments, sheared and cross-linked chromatin along with GR-antibody (N499) was sent to the Peconic Company for further processing. For more details, see Supplemental Experimental Procedures.

### EMSAs with and without formaldehyde cross-linking

EMSAs were performed as previously described (Thomas-Chollier et al. 2013) with several modifications to assay the efficiency of in vitro cross-linking. For more details, see the Supplemental Experimental Procedures.

### ExoProfiler pipeline, computational analysis

To analyze the local 5′ coverage distribution centered on TFBSs, we developed a computational pipeline called ExoProfiler (Fig. 1B). It takes as input the mapped reads from a ChIP-exo experiment and a list of peak coordinates. It first computes small regions centered on a motif of interest and calculates the coverage considering only the most 5′ position of the reads as it marks the boundary of protection from lambda exonuclease digestion provided by cross-linked proteins. The pipeline outputs four plots, including a heatmap of the 5′ coverage combining the forward and reverse strand and a footprint profile, summing the coverage at each position for all regions, for the forward and reverse strand. Details of the ExoProfiler pipeline and of all computational analysis (BeadChip gene expression analysis; ChIP-seq and ChIP-exo processing; scanning peaks for motif hits, and structural alignments) can be found in the Supplemental Experimental Procedures.

## Data access

Raw gene expression profiling (BeadChip) and raw and processed ChIP-seq and ChIP-exo data have been submitted to EBI ArrayExpress (http://www.ebi.ac.uk/arrayexpress) under accession numbers E-MTAB-2954, E-MTAB-2955, and E-MTAB-2956, respectively. We provide the source code for the ExoProfiler method in the Supplemental Material as a free and open source tool to the community (https://github.com/ComputationalSystemsBiology/ExoProfiler).

## Supplementary Material

Supplemental Material
