# Supplementary material for: ChIP-exo signal associated with DNA-binding motifs provides insight into the genomic binding of the glucocorticoid receptor and cooperating transcription factors
Source: Genome Res. 2015 Jun;25(6):825–35. doi: 10.1101/gr.185157.114 (PMC4448679; doi:10.1101/gr.185157.114)
Supplement: Supplemental Material [file supp_25_6_825__index.html]

ChIP-exo signal associated with DNA-binding motifs provides insight into the genomic binding of the glucocorticoid receptor and cooperating transcription factors — ChIP-exo signal associated with DNA-binding motifs provides insight into the genomic binding of the glucocorticoid receptor and cooperating transcription factors — Supplemental Material 

# ChIP-exo signal associated with DNA-binding motifs provides insight into the genomic binding of the glucocorticoid receptor and cooperating transcription factors

## Supplemental Material

**Files in this Data Supplement:**

- Supplemental Data 2.pdf
- Supplemental Data 3.pdf
- Supplemental Material.pdf
- ExoProfiler-master.zip
