# Supplementary material for: ChIP-exo signal associated with DNA-binding motifs provides insight into the genomic binding of the glucocorticoid receptor and cooperating transcription factors
Source: Genome Res. 2015 Jun;25(6):825–35. doi: 10.1101/gr.185157.114 (PMC4448679; doi:10.1101/gr.185157.114)
Supplement: Supplemental Material [file supp_gr.185157.114_Supplemental_Data_2.pdf]

Supplementary data 2

Starick, Ibn-Salem, Jurk et al *ChIP-exo signal associated with DNA-binding motifs provide insights into the genomic binding of the glucocorticoid receptor and cooperating transcription factors.*

Table presenting the profiles obtained with ExoProfiler in IMR90 cell line.

Here, ChIP-seq peaks were limited to ±30 bp around the peak summit. The motifs were obtained from JASPAR vertebrates only or discovered for this peak dataset with the program RSAT *peak-motifs*.
The table displays the profiles for all motifs; the following information are indicated: the provenance (JASPAR ID or de novo) and its name (from JASPAR, or for de novo motifs: name of the most similar motif within JASPAR, similarity values taken from *peak-motifs* result), the class and family of TF (information from JASPAR), motif logo, heatmap and profile, number of sites contributing to the profile, coverage p-value.

Table can be sorted by clicking on the column headers. For our analyses, this table was sorted on the coverage p-value (last column), lowest values first.

Factor/motif	Class	Family	Logo and exo-profile	Sites	Coverage p-value ▾
peaks-motifs de novo discovery<br>**dyads_m1**<br>Most similar motif in JASPAR:<br>**NR3C1**	-	-	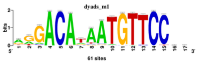 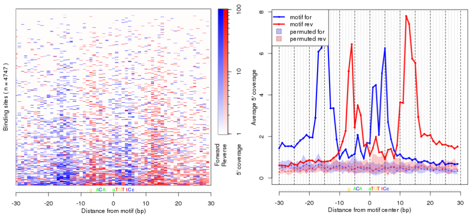	4747	0
**MA0007.2**<br>**AR**	Zinc-coordinating	Hormone-nuclear Receptor	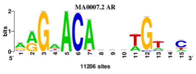 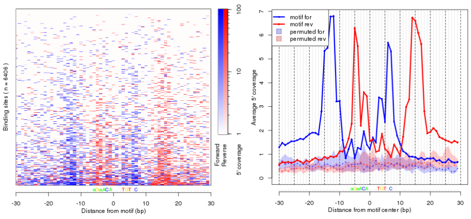	6406	0
**MA0113.2**<br>**NR3C1**	Zinc-coordinating	Hormone-nuclear Receptor	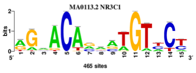 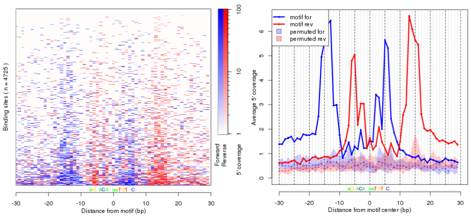	5446	0
peaks-motifs de novo discovery<br>**positions_6nt_m1**<br>Most similar motif in JASPAR:<br>**NR3C1**	-	-	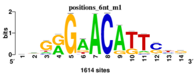 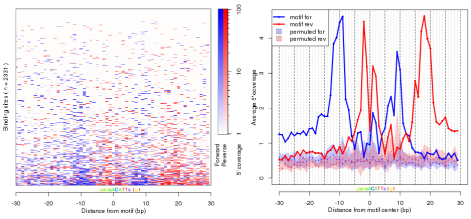	2331	7.6484320503644e-153
peaks-motifs de novo discovery<br>**positions_7nt_m1**<br>Most similar motif in JASPAR:<br>**NR3C1**	-	-	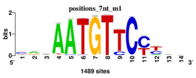 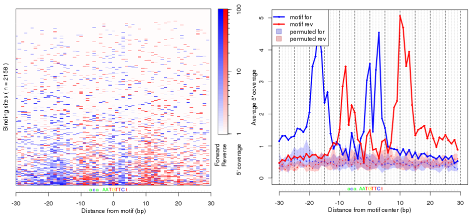	2158	3.51766965091321e-140
peaks-motifs de novo discovery<br>**positions_6nt_m2**<br>Most similar motif in JASPAR:<br>**NR3C1**	-	-	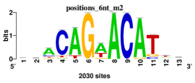 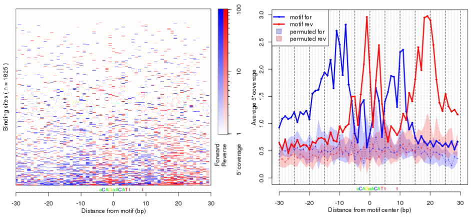	1825	1.08207690980014e-84
			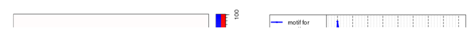		

peaks-motifs de novo discovery<br>**positions_7nt_m5**<br>Most similar motif in JASPAR:<br>**AR**	-	-	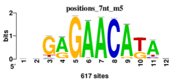	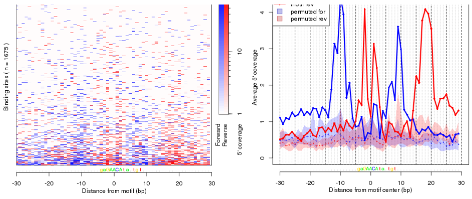		1675	2.03885290161182e-79
peaks-motifs de novo discovery<br>**positions_7nt_m3**<br>Most similar motif in JASPAR:<br>**NR3C1**	-	-	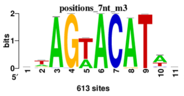	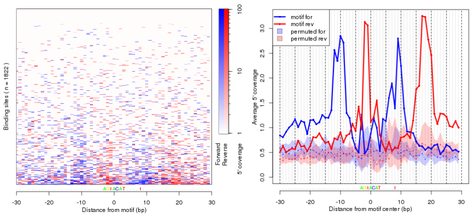		1822	3.12451482639409e-77
**MA0031.1**<br>**FOXD1**	Winged Helix-Turn-Helix	Forkhead	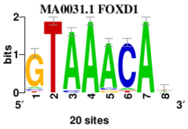	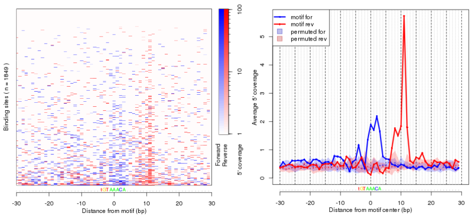		1849	4.49121080362848e-46
**MA0148.3**<br>**FOXA1**	Winged Helix-Turn-Helix	Forkhead	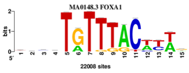	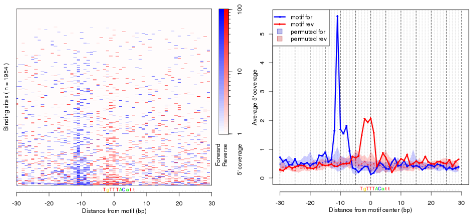		1954	4.23150393132508e-44
**MA0047.2**<br>**Foxa2**	Winged Helix-Turn-Helix	Forkhead	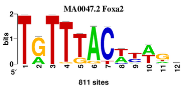	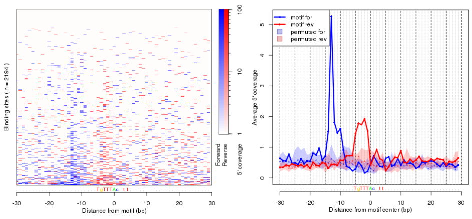		2194	4.59197499137264e-41
**MA0593.1**<br>**FOXP2**	Winged Helix-Turn-Helix	Forkhead	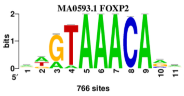	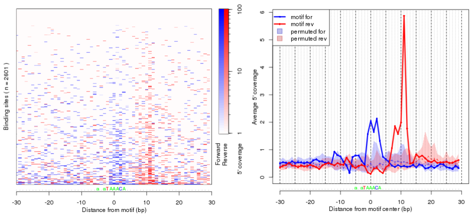		2601	1.0560916695928e-40
				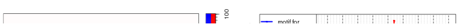			

**MA0481.1**<br><br>**FOXP1**	Winged Helix-Turn-Helix	Forkhead	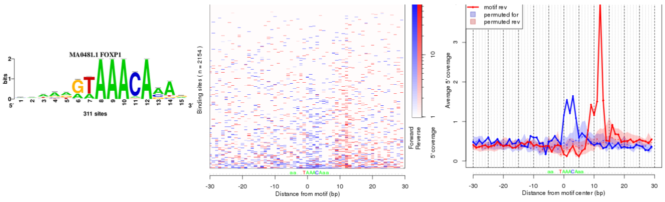	2154	3.99171738733038e-38
**MA0480.1**<br><br>**Foxo1**	Winged Helix-Turn-Helix	Forkhead	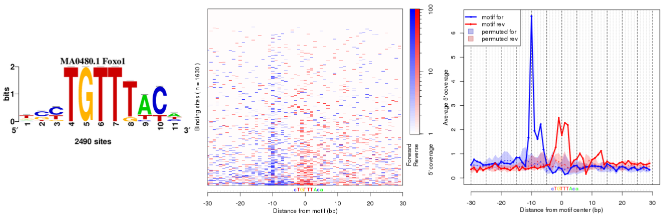	1630	2.57865464984525e-37
peaks-motifs de novo discovery<br>**oligos_6nt_mkv4_m2**<br><br>Most similar motif in JASPAR:<br><br>**FOXP1**	-	-	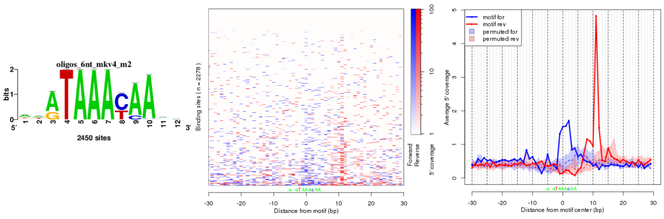	2278	1.22386621222197e-33
**MA0040.1**<br><br>**Foxq1**	Winged Helix-Turn-Helix	Forkhead	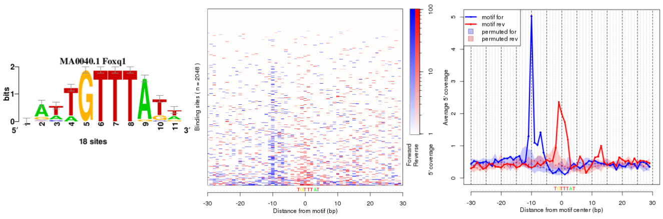	2048	2.43935496405333e-32
**MA0030.1**<br><br>**FOXF2**	Winged Helix-Turn-Helix	Forkhead	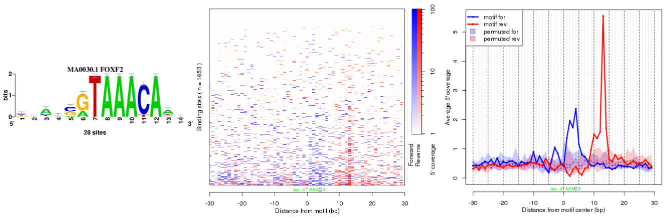	1653	4.63705298388167e-29
peaks-motifs de novo discovery					

**positions_7nt_m4**<br>Most similar motif in JASPAR:<br>**no match**	-	-	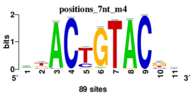	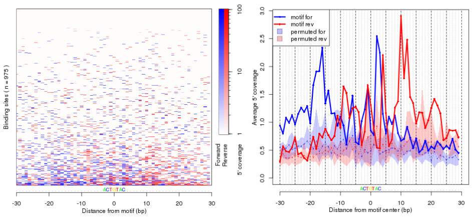	975	7.78453602586915e-28
peaks-motifs de novo discovery<br>**dyads_m2**<br>Most similar motif in JASPAR:<br>**FOXP1**	-	-	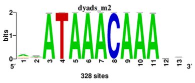	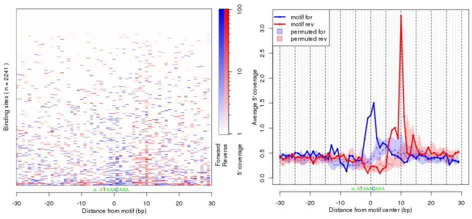	2241	3.4958711561983e-27
peaks-motifs de novo discovery<br>**dyads_m5**<br>Most similar motif in JASPAR:<br>**FOXO3**	-	-	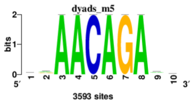	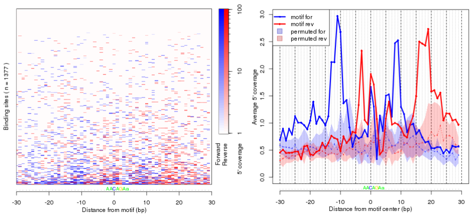	1377	1.07937586662635e-23
**MA0112.2**<br>**ESR1**	Zinc-coordinating	Hormone-nuclear Receptor	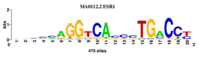	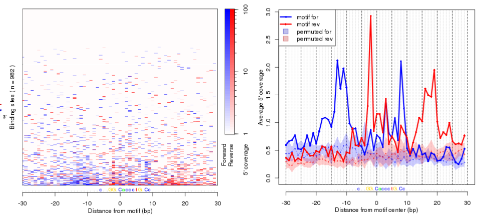	995	1.58989155855649e-21
**MA0157.1**<br>**FOXO3**	Winged Helix-Turn-Helix	Forkhead	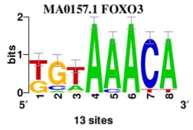	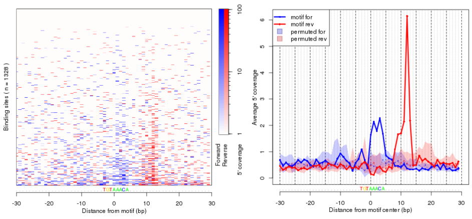	1328	1.18056872694808e-19
**MA0476.1**<br>**FOS**	Zipper-Type	Leucine-Zipper	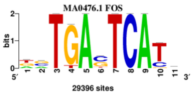	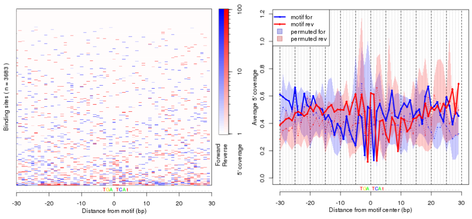	4781	7.48361482735651e-17
**MA0042.1**<br>**FOXI1**	Winged Helix-Turn-Helix	Forkhead	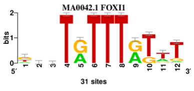	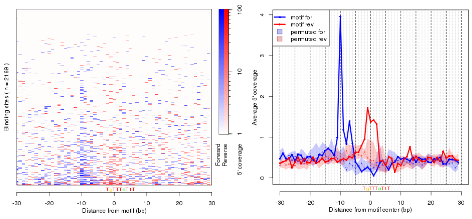	2169	1.32378885419659e-16
				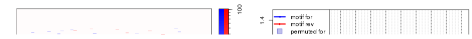		

**MA0102.3**<br>**CEBPA**	Zipper-Type	Leucine Zipper	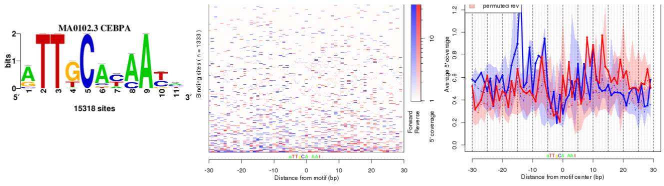	1333	1.82047788387476e-16
peaks-motifs de novo discovery<br>**oligos_6nt_mkv4_m5**<br>Most similar motif in JASPAR:<br>**TEAD1**	-	-	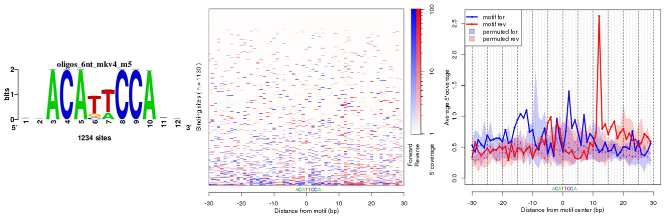	1130	1.09549433426823e-15
**MA0098.2**<br>**Ets1**	Winged Helix-Turn-Helix	Ets	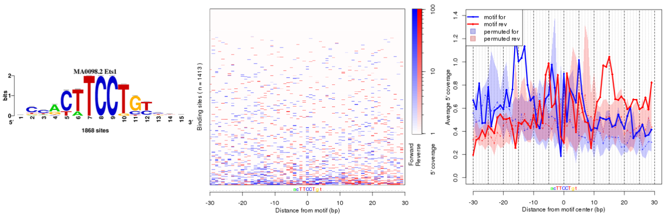	1413	2.41392734793751e-14
**MA0466.1**<br>**CEBPB**	Zipper-Type	Leucine-Zipper	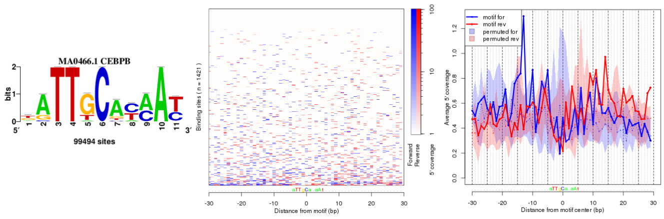	1421	1.69831565425716e-13
**MA0002.2**<br>**RUNX1**	Ig-fold	Runt	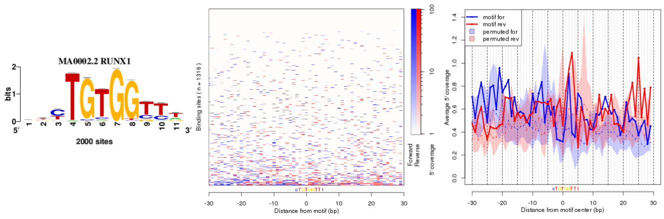	1316	8.93179154000296e-13
**MA0074.1**<br>**RXRA::VDR**	Zinc-coordinating	Hormone-nuclear Receptor	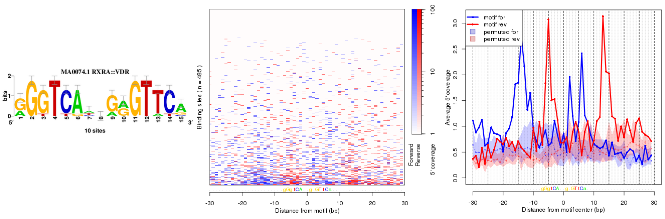	485	2.8222672462489e-11

**MA0486.1** **HSF1**	Winged Helix-Turn-Helix	HSF	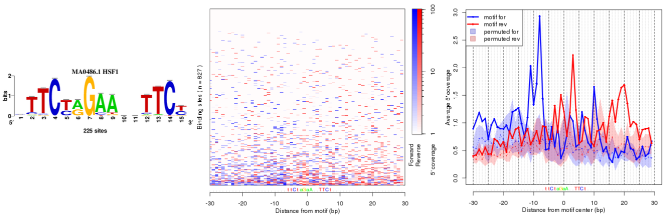	827	4.19949736616783e-11
peaks-motifs de novo discovery **positions_6nt_m3** Most similar motif in JASPAR: **FOXO3**	-	-	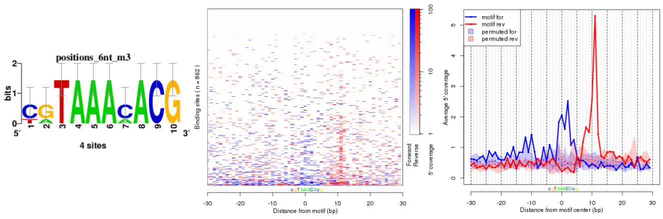	864	6.00817485313302e-11
**MA0090.1** **TEAD1**	Helix-Turn-Helix	Homeo	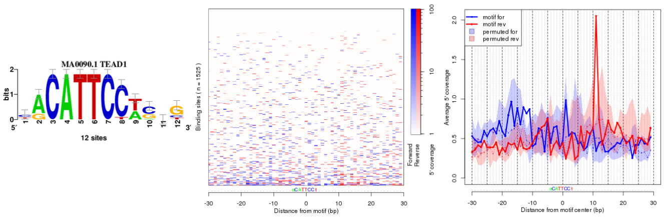	1525	4.42370836416079e-10
peaks-motifs de novo discovery **oligos_7nt_mkv5_m3** Most similar motif in JASPAR: **CEBPA**	-	-	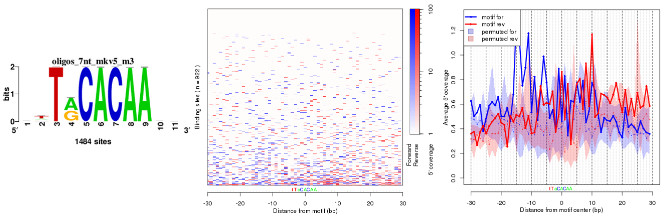	922	9.13676637209637e-10
**MA0511.1** **RUNX2**	Other	Runt	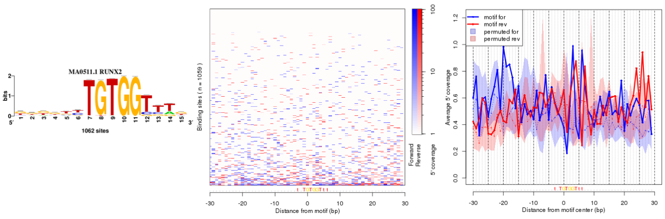	1059	1.34664982128199e-09
**MA0519.1** **Stat5a::Stat5b**	Other	STAT	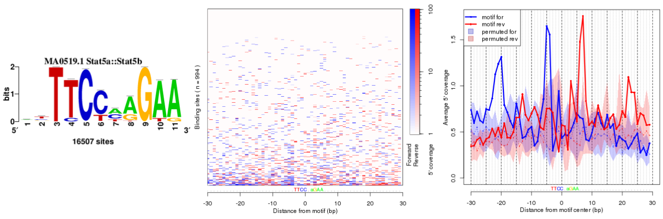	994	1.18243949876918e-08

**MA0084.1**<br>**SRY**	Other Alpha-Helix	High Mobility Group box (HMG)	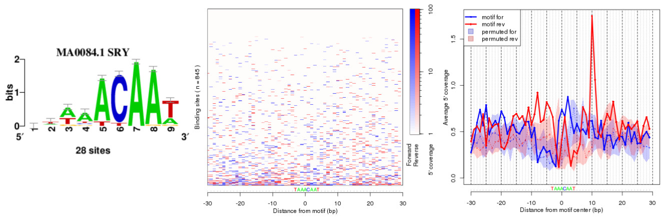	845	1.38914048905346e-08
peaks-motifs de novo discovery<br>**dyads_m4**<br>Most similar motif in JASPAR:<br>**RUNX1**	-	-	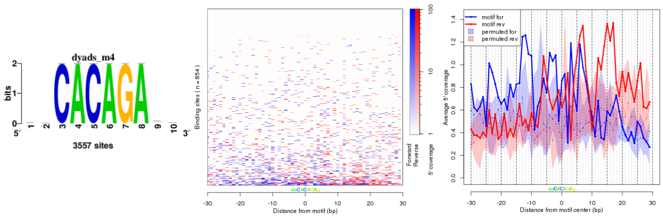	854	2.32932876433485e-07
peaks-motifs de novo discovery<br>**oligos_7nt_mkv5_m5**<br>Most similar motif in JASPAR:<br>**FOXD1**	-	-	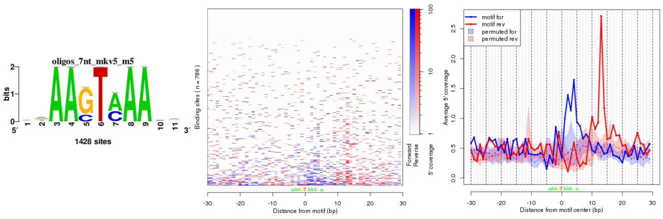	786	2.50831244075935e-07
**MA0490.1**<br>**JUNB**	Zipper-Type	Leucine-Zipper	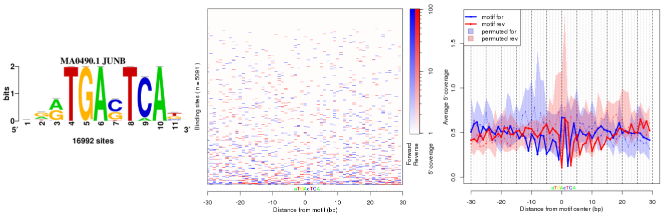	5091	3.6665521542343e-07
**MA0144.2**<br>**STAT3**	Ig-fold	Stat	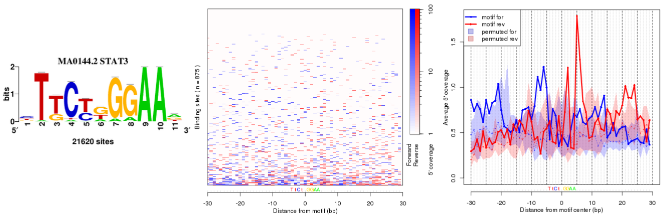	971	4.23241334622616e-07
**MA0041.1**	Winged Helix-Turn-	Forkhead	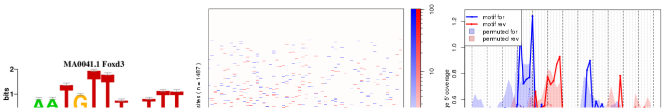	1487	8.09679418205736e-07

Foxd3	Helix		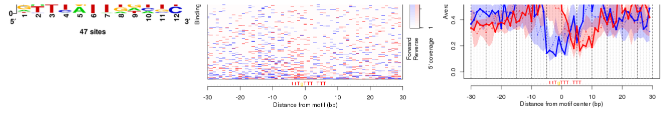		
**MA0491.1** JUND	Zipper-Type	Leucine-Zipper	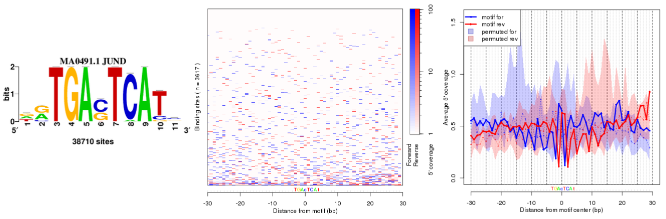	5257	9.45617251321679e-07
**MA0462.1** BATF::JUN	Zipper-Type	Leucine-Zipper	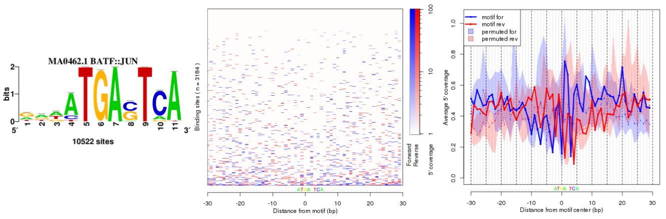	3184	4.56203850255607e-06
**MA0504.1** NR2C2	Zinc-coordinating	Hormone-nuclear Receptor	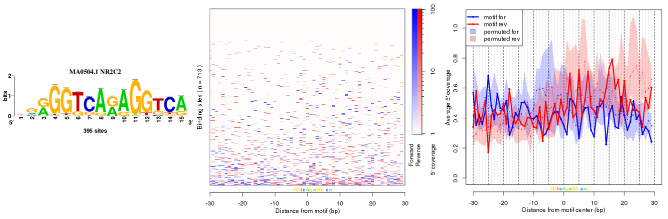	713	5.78695776146218e-06
**MA0137.3** STAT1	Ig-fold	Stat	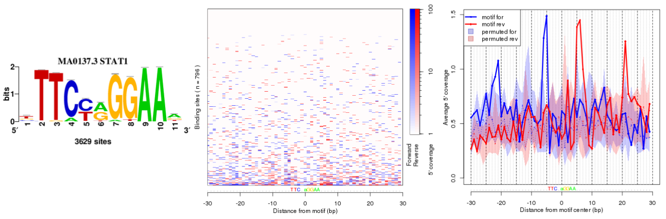	990	6.39253273924696e-06
**MA0025.1** NFIL3	Zipper-Type	Leucine Zipper	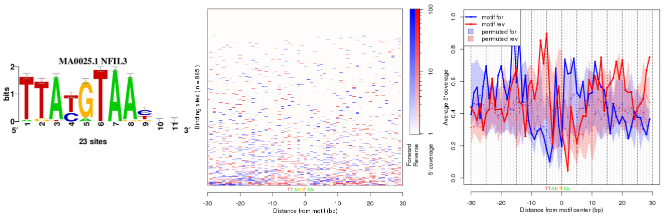	865	1.19282211535992e-05
**MA0518.1** Stat4	Other	STAT	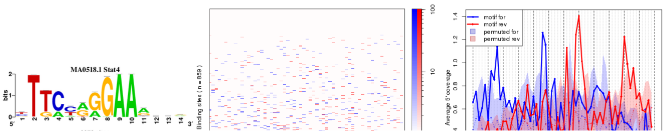	859	3.9121430981811e-05

			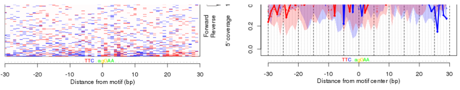		
**MA0091.1**<br><br>**TAL1::TCF3**	Zipper-Type	Helix-Loop-Helix	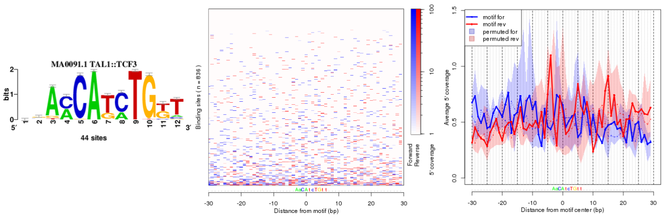	836	8.61319069330976e-05
**MA0065.2**<br><br>**PPARG::RXRA**	Zinc-coordinating	Hormone-nuclear Receptor	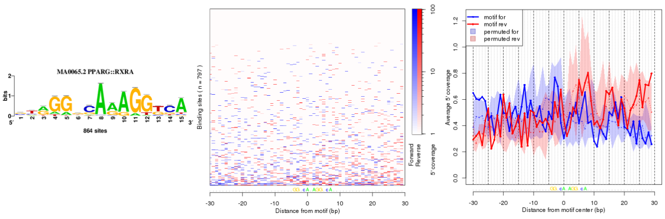	797	0.00018832778358369
**MA0159.1**<br><br>**RXR::RAR_DR5**	Zinc-coordinating	Hormone-nuclear Receptor	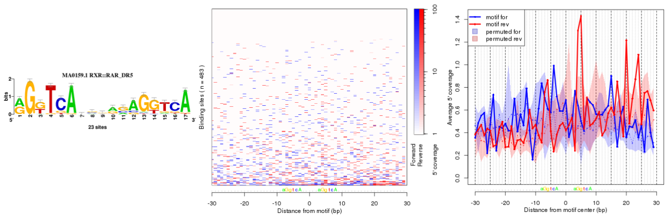	483	0.000208374262875992
**MA0474.1**<br><br>**Erg**	Winged Helix-Turn-Helix	ETS	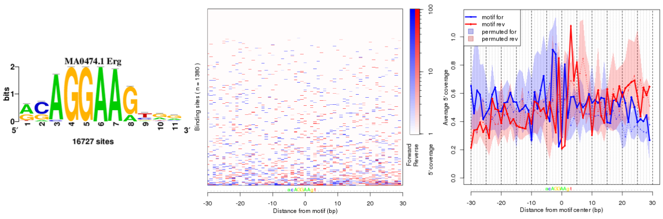	1380	0.000230007148981826
**MA0143.3**<br><br>**Sox2**	Other Alpha-Helix	High Mobility Group box (HMG)	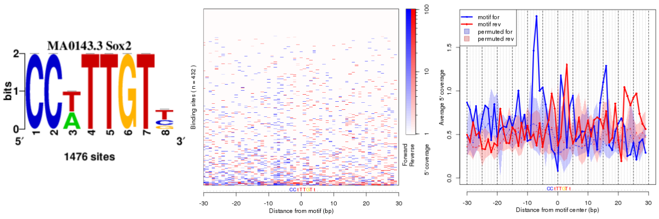	432	0.000416155067541784

**MA0598.1**<br>**EHF**	Winged Helix-Turn-Helix	ETS	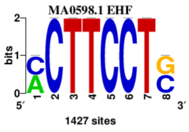	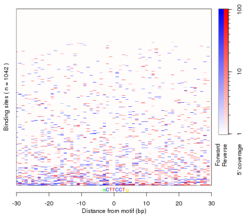	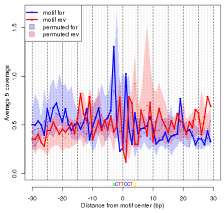	1042	0.000480306553380232
**MA0488.1**<br>**JUN**	Zipper-Type	Leucine-Zipper	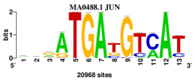	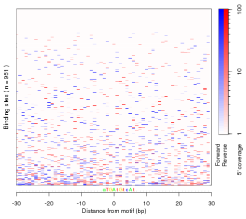	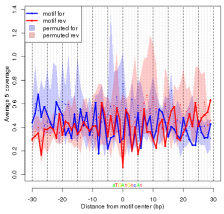	951	0.000624662576451088
**MA0510.1**<br>**RFX5**	Winged Helix-Turn-Helix	RFX	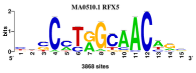	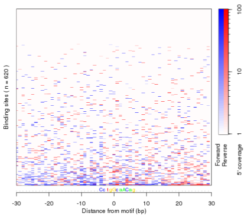	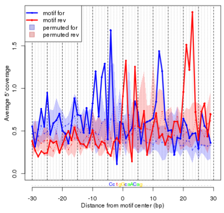	620	0.00067970247027751
**MA0500.1**<br>**Myog**	Zipper-Type	Helix-Loop-Helix	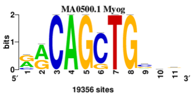	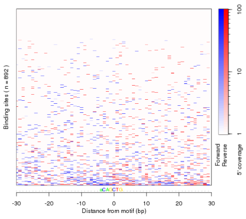	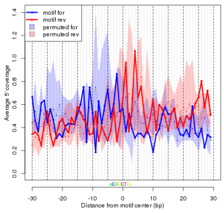	892	0.000767168494416453
**MA0043.1**<br>**HLF**	Zipper-Type	Leucine Zipper	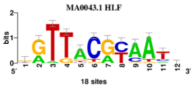	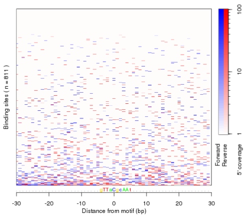	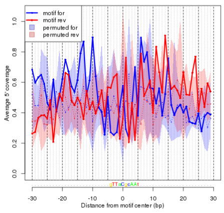	952	0.000823400659650455
peaks-motifs de novo discovery<br>**oligos_6nt_mkv4_m4**<br>Most similar motif in JASPAR:<br>**no match**	-	-	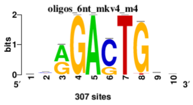	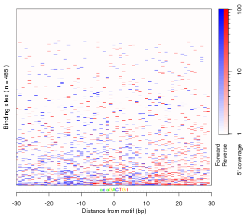	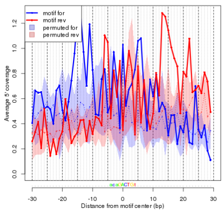	485	0.00101766137890243

**MA0156.1**<br>**FEV**	Winged Helix-Turn-Helix	Ets	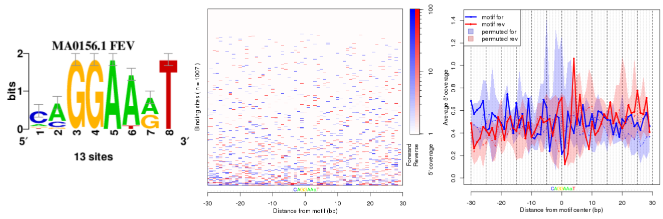	1007	0.00104345410857944
**MA0080.3**<br>**Spi1**	Winged Helix-Turn-Helix	Ets	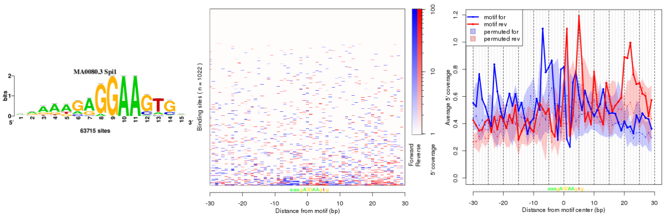	1022	0.00127709428898886
**MA0162.2**<br>**EGR1**	Zinc-coordinating	BetaBetaAlpha-zinc finger	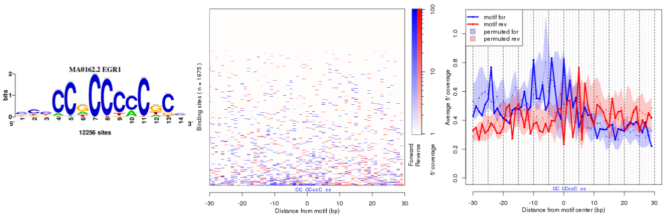	1675	0.00155680570056084
**MA0484.1**<br>**HNF4G**	Zinc-coordinating	Hormone-nuclear Receptor	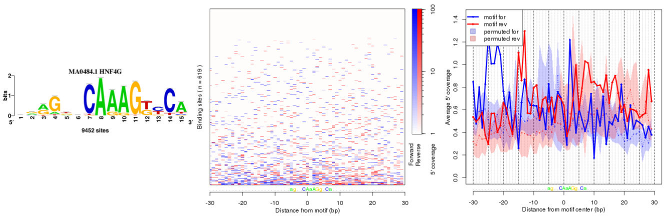	619	0.00214489153997349
			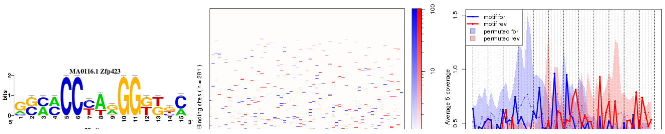		

**MA0116.1** **Zfp423**	Zinc-coordinating	BetaBetaAlpha-zinc finger	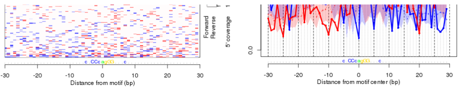	327	0.00231344393798243
**MA0027.1** **En1**	Helix-Turn-Helix	Homeo	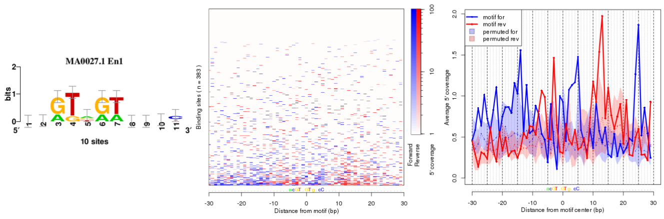	383	0.00248195909745266
**MA0258.2** **ESR2**	Zinc-coordinating	Hormone-nuclear Receptor	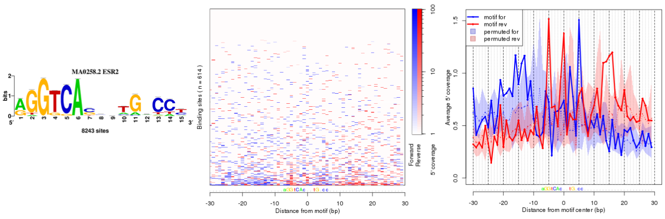	682	0.00250579272770723
**MA0520.1** **Stat6**	Other	STAT	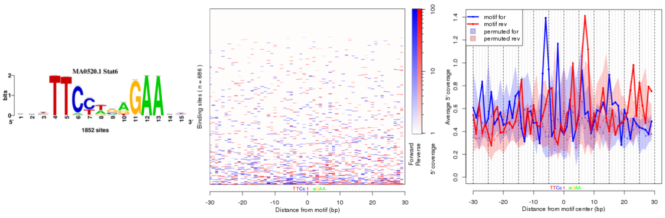	686	0.00252942991239785
**MA0521.1** **Tcf12**	Zipper-Type	Helix-Loop-Helix	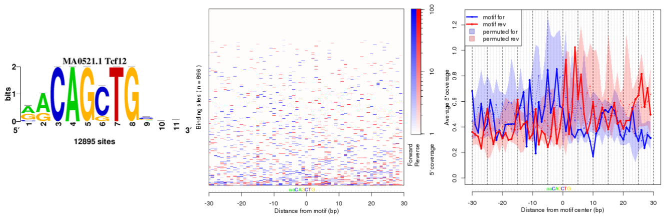	896	0.00319389811577332
**MA0106.2** **TP53**	Zinc-coordinating	Loop-Sheet-Helix	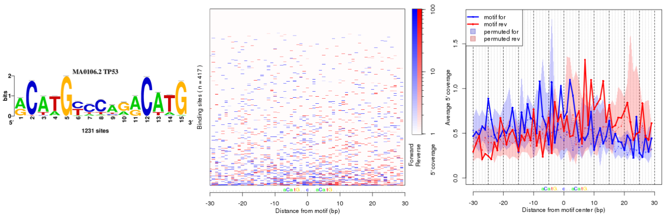	417	0.00352369089012948

**MA0088.1**<br>**znf143**	Zinc-coordinating	BetaBetaAlpha-zinc finger	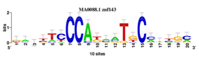	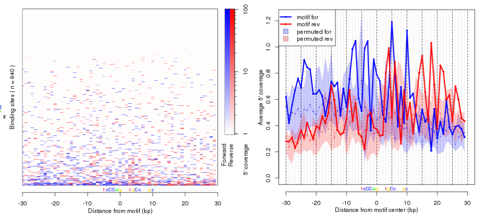	640	0.003984932888394
**MA0524.1**<br>**TFAP2C**	Zipper-Type	Helix-Loop-Helix	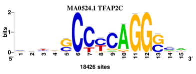	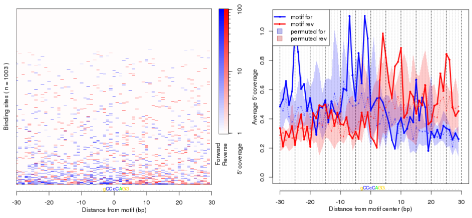	1003	0.00438767891428064
**MA0114.2**<br>**HNF4A**	Zinc-coordinating	Hormone-nuclear Receptor	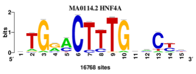	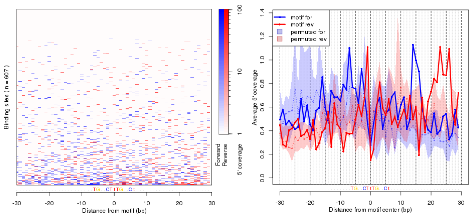	607	0.00565504518682653
**MA0591.1**<br>**Bach1::Mafk**	Zipper-Type	Leucine-Zipper	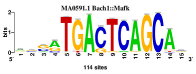	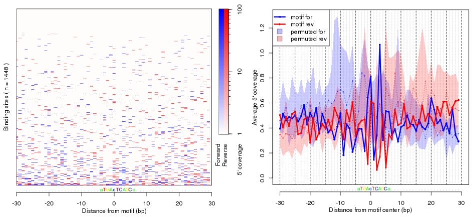	1525	0.00570333950599349
**MA0062.2**<br>**GABPA**	Winged Helix-Turn-Helix	Ets	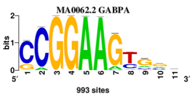	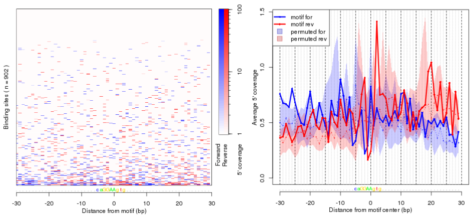	902	0.00685419043629559
**MA0136.1**<br>**ELF5**	Winged Helix-Turn-Helix	Ets	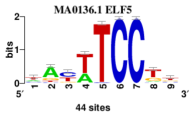	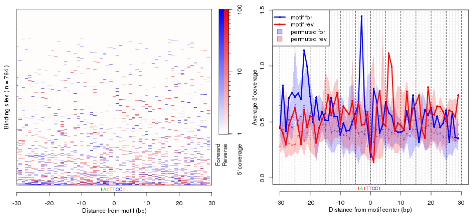	764	0.00803287309888239
peaks-motifs de novo discovery<br>**oligos_7nt_mkv5_m1**<br>Most similar motif in JASPAR:<br>**FOS**	-	-	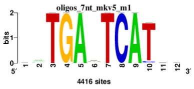	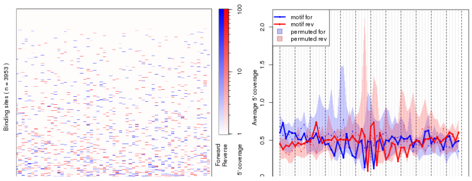	3953	0.0161926102625433

			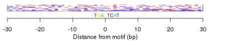 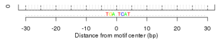		
**MA0048.1** **NHLH1**	Zipper-Type	Helix-Loop-Helix	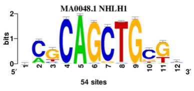 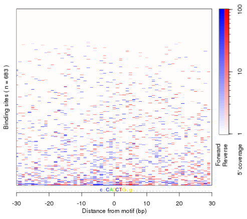 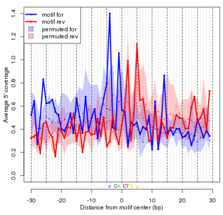	1010	0.0204372839692601
**MA0081.1** **SPIB**	Winged Helix-Turn-Helix	Ets	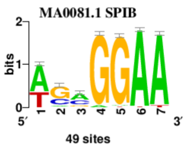 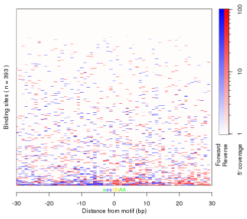 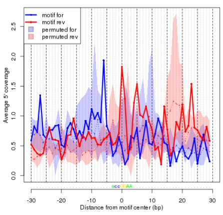	393	0.0234816883117741
**MA0108.2** **TBP**	Beta-sheet	TATA-binding	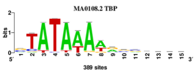 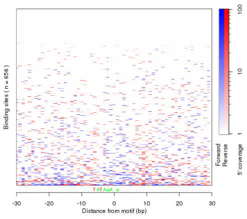 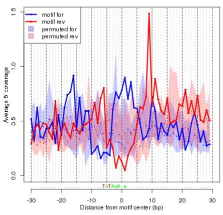	656	0.025177112849857
**MA0145.2** **Tcfcp2l1**	Other	CP2	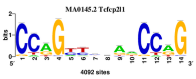 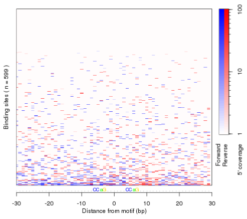 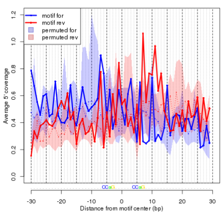	710	0.0256010752634432
**MA0071.1** **RORA_1**	Zinc-coordinating	Hormone-nuclear Receptor	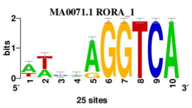 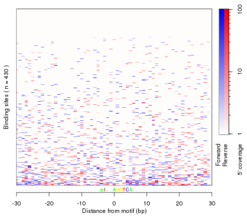 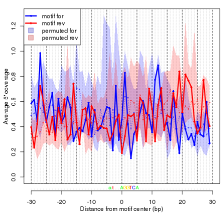	430	0.0326530735291033
**MA0066.1** **PPARG**	Zinc-coordinating	Hormone-nuclear Receptor	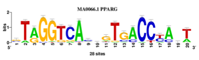 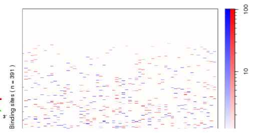 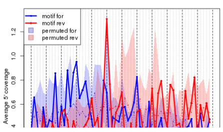	391	0.0366902896092156

			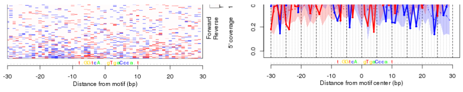		
**MA0109.1** **Hltf**	Zinc-coordinating	GATA	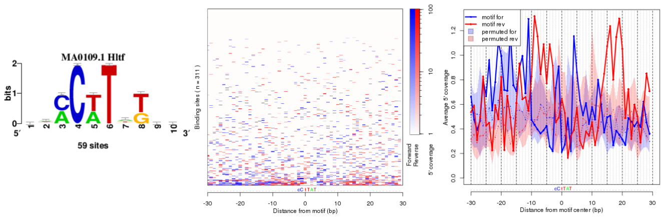	312	0.0416190328012254
**MA0058.2** **MAX**	Zipper-Type	Helix-Loop-Helix	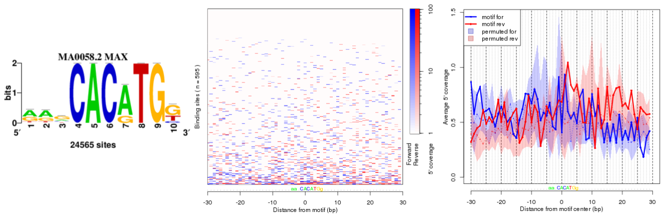	595	0.0462753676723501
**MA0473.1** **ELF1**	Winged Helix-Turn-Helix	ETS	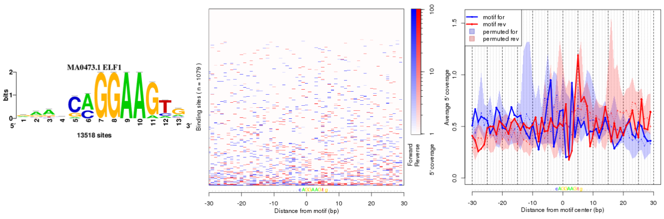	1079	0.0506398977427855
**MA0004.1** **Arnt**	Zipper-Type	Helix-Loop-Helix	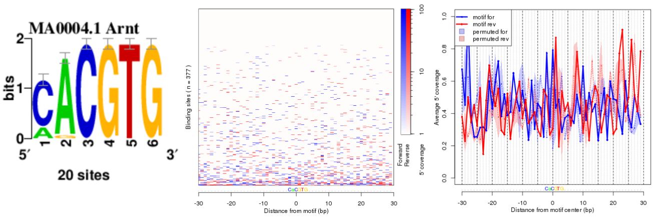	647	0.0507508061519212
**MA0522.1** **Tcf3**	Other Alpha-Helix	High Mobility Group (Box)	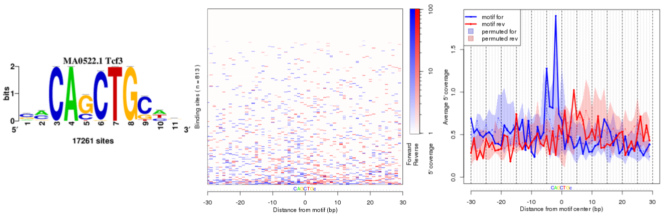	813	0.057721714706671
**MA0147.2** **Myc**	Zipper-Type	Helix-Loop-Helix	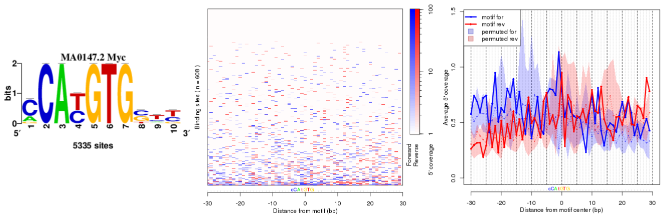	608	0.0580931669032525

**MA0056.1**<br>**MZF1_1-4**	Zinc-coordinating	BetaBetaAlpha-zinc finger	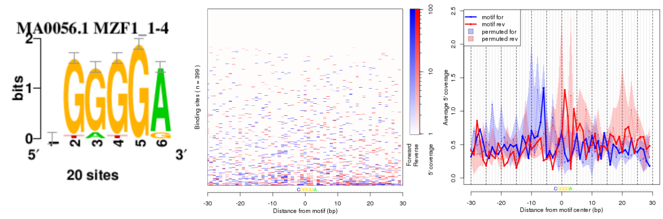	399	0.0615687876335193
peaks-motifs de novo discovery<br>**dyads_m3**<br>Most similar motif in JASPAR:<br>**FOSL2**	-	-	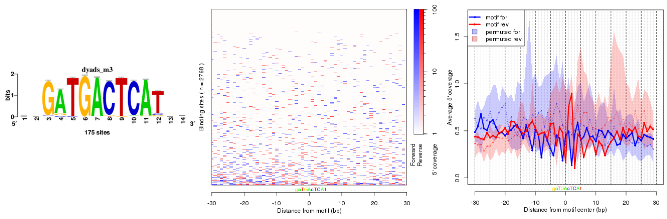	2768	0.062156700192963
**MA0052.2**<br>**MEF2A**	Other Alpha-Helix	MADS	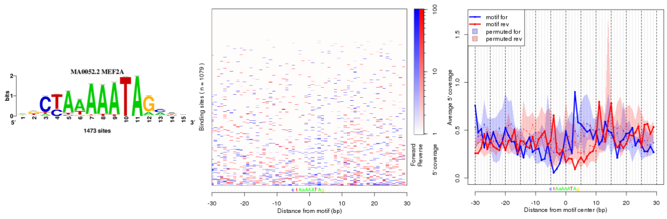	1079	0.065122604733139
**MA0039.2**<br>**Klf4**	Zinc-coordinating	BetaBetaAlpha-zinc finger	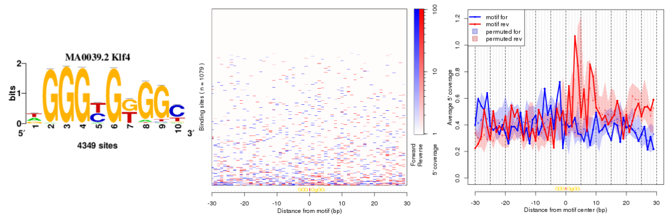	1079	0.0651612670726308
**MA0032.1**<br>**FOXC1**	Winged Helix-Turn-Helix	Forkhead	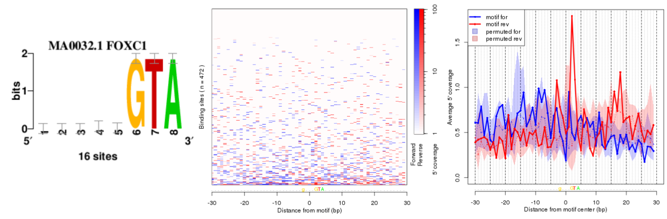	473	0.068536444559146
**MA0525.1**<br>**TP63**	Zinc-coordinating	Loop-Sheet-Helix	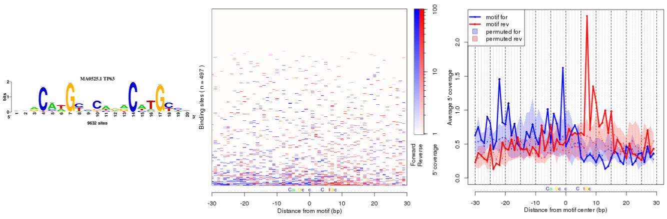	557	0.0729148841020679
			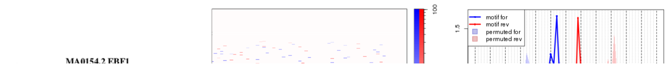		

**MA0154.2**<br>**EBF1**	Zipper-Type	Helix-Loop-Helix	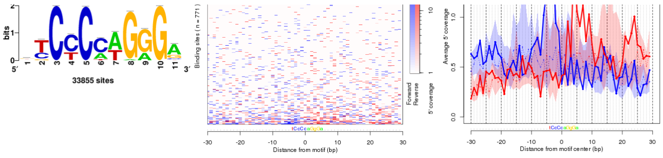	771	0.0831758701283423
**MA0596.1**<br>**SREBF2**	Zipper-Type	Helix-Loop-Helix	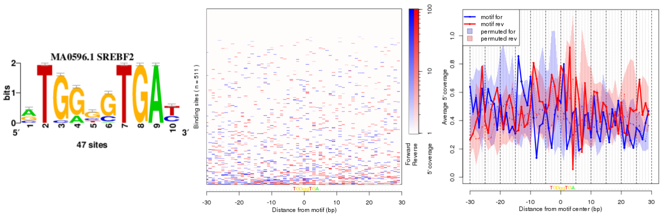	513	0.0990809960496176
**MA0499.1**<br>**Myod1**	Zipper-Type	Helix-Loop-Helix	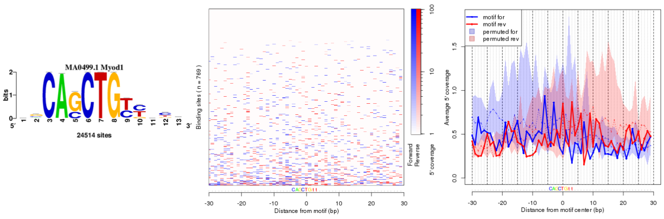	769	0.10218294215897
**MA0059.1**<br>**MYC::MAX**	Zipper-Type	Helix-Loop-Helix	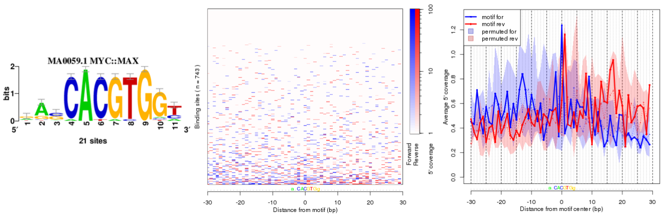	743	0.102585063812316
**MA0037.2**<br>**GATA3**	Zinc-coordinating	GATA	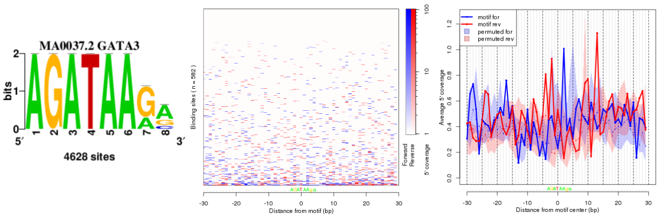	582	0.103546557390364
**MA0471.1**<br>**E2F6**	Winged Helix-Turn-Helix	E2F	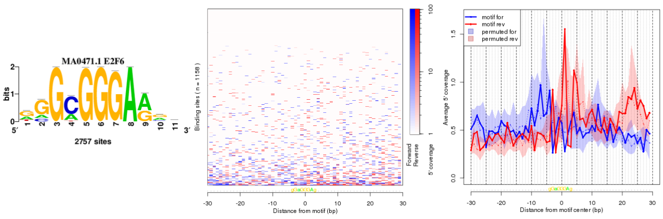	1158	0.105741025542785
			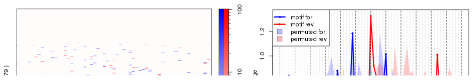		

**MA0494.1**<br>**Nr1h3::Rxra**	Zinc-coordinating	Hormone-nuclear Receptor	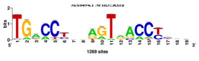	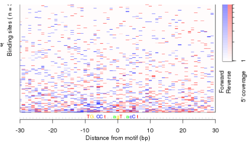	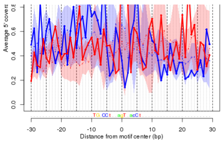	379	0.105876899193544
**MA0068.1**<br>**Pax4**	Helix-Turn-Helix	Homeo	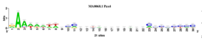	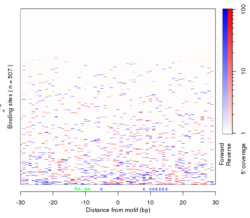	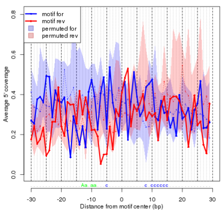	509	0.110004954169175
**MA0006.1**<br>**Arnt::Ahr**	Zipper-Type	Helix-Loop-Helix	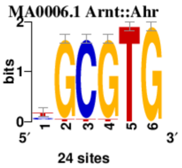	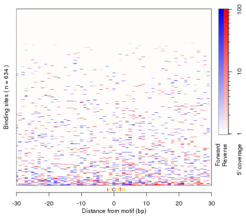	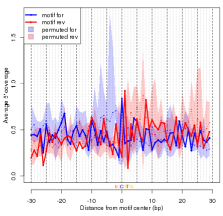	704	0.114762150810417
**MA0019.1**<br>**Ddit3::Cebpa**	Zipper-Type	Leucine Zipper	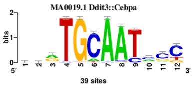	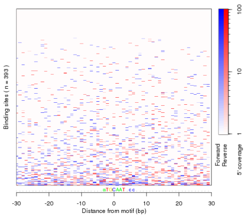	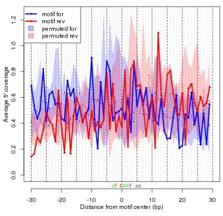	393	0.121760463472309
**MA0067.1**<br>**Pax2**	Helix-Turn-Helix	Homeo	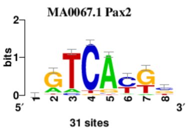	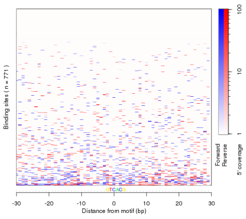	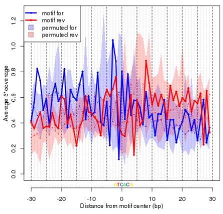	771	0.124076205527151
**MA0259.1**<br>**HIF1A::ARNT**	Zipper-Type	Helix-Loop-Helix				941	0.127166263922089

			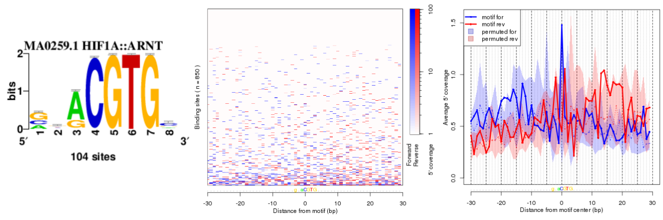		
**MA0505.1** **Nr5a2**	Zinc-coordinating	Hormone-nuclear Receptor	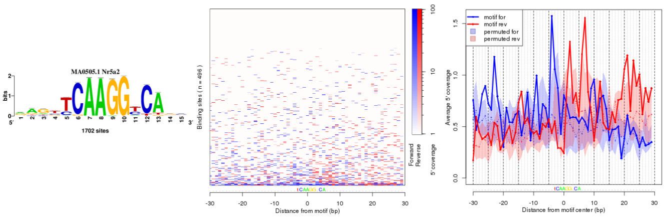	496	0.129250925262028
**MA0003.2** **TFAP2A**	Zipper-Type	Helix-Loop-Helix	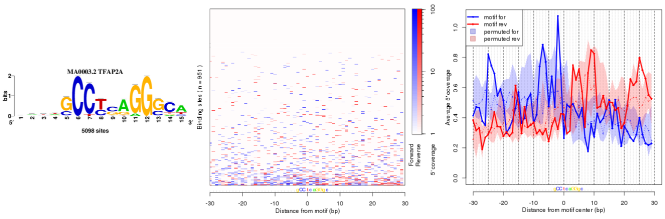	951	0.139076289226506
**MA0475.1** **FLI1**	Winged Helix-Turn-Helix	ETS	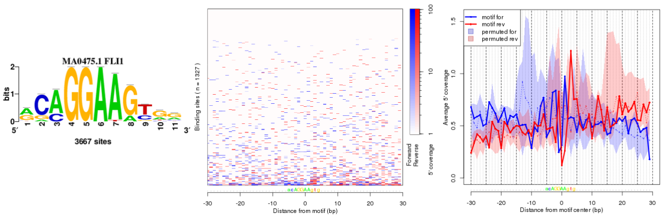	1327	0.140805488622663
**MA0107.1** **RELA**	Ig-fold	Rel	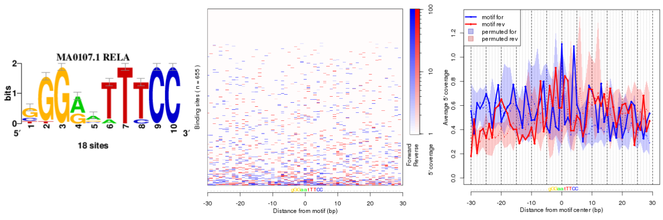	671	0.141943934209372
			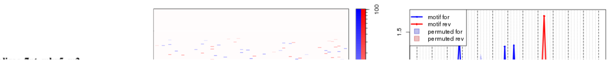		

peaks-motifs de novo discovery<br>**oligos_7nt_mkv5_m2**<br>Most similar motif in JASPAR:<br>**no match**	-	-	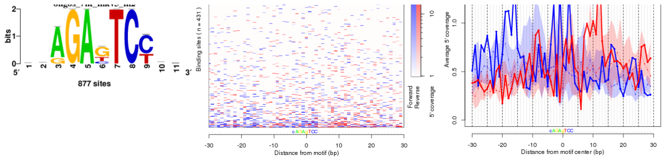	438	0.156453743912521
**MA0138.2**<br>**REST**	Zinc-coordinating	BetaBetaAlpha-zinc finger	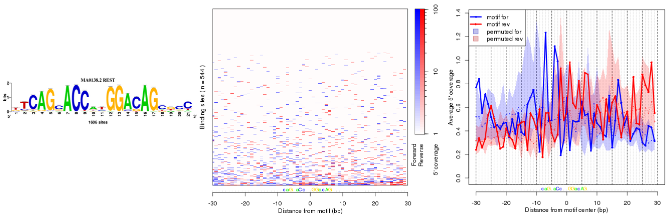	544	0.156723736354918
**MA0155.1**<br>**INSM1**	Zinc-coordinating	BetaBetaAlpha-zinc finger	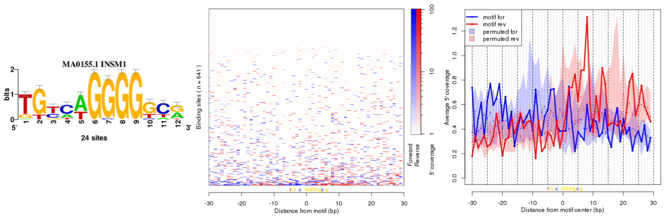	641	0.168203056821478
**MA0119.1**<br>**TLX1::NFIC**	Helix-Turn-Helix::Other	Homeo::Nuclear Factor I-CCAAT-binding	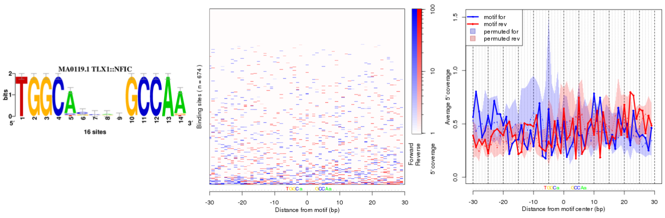	674	0.168915130035081
**MA0035.3**<br>**Gata1**	Zinc-coordinating	GATA	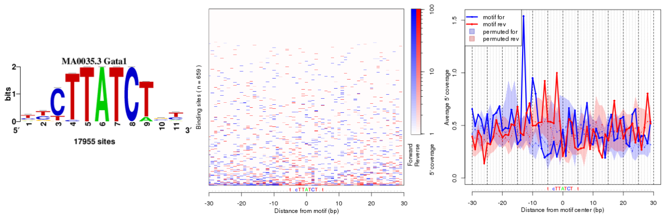	659	0.170799762695913
**MA0469.1**<br>**E2F3**	Winged Helix-Turn-Helix	E2F	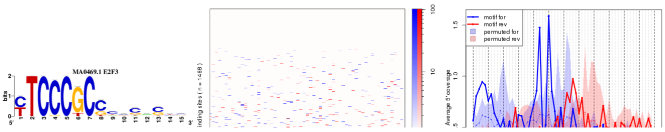	1468	0.173666628461744

				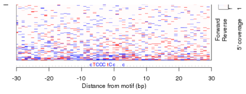	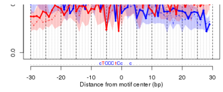		
**MA0461.1**<br>**Atoh1**	Zipper-Type	Helix-Loop-Helix	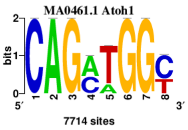	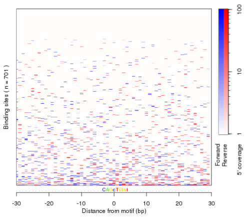	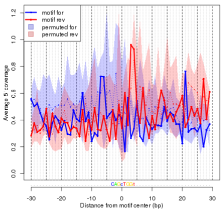	701	0.176198390608434
**MA0141.2**<br>**Esrrb**	Zinc-coordinating	Hormone-nuclear Receptor	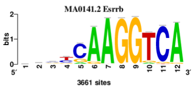	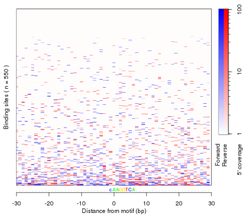	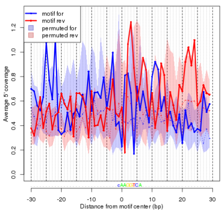	550	0.177250282145084
**MA0516.1**<br>**SP2**	Zinc-coordinating	BetaBetaAlpha-zinc Finger	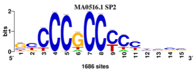	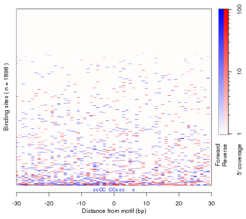	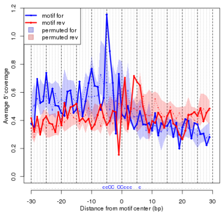	1898	0.181517447418176
**MA0479.1**<br>**FOXH1**	Winged Helix-Turn-Helix	Forkhead	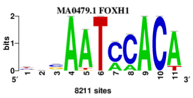	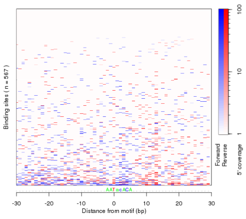	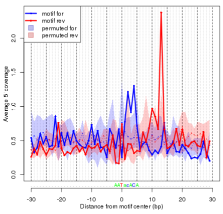	567	0.183120706866782
**MA0036.2**<br>**GATA2**	Zinc-coordinating	GATA	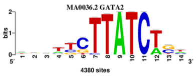	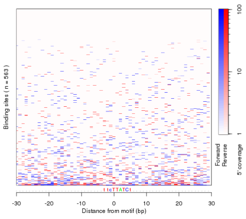	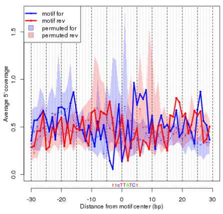	563	0.188137663212389
**MA0131.1**	Zinc-	BetaBetaAlpha-				[illegible]	[illegible]

**HINFP**	coordinating	zinc finger	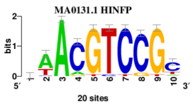	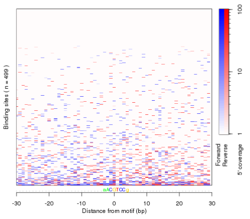	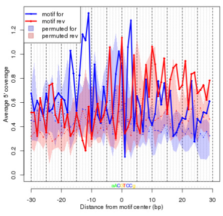	[illegible]	[illegible]
**MA0528.1** **ZNF263**	Zinc-coordinating	BetaBetaAlpha-zinc Finger	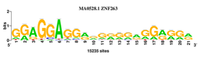	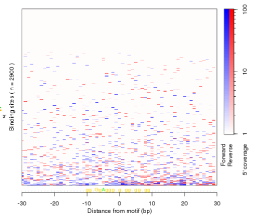	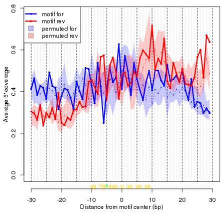	2900	0.211052292796158
**MA0592.1** **ESRRA**	Zinc-coordinating	Hormone-nuclear Receptor	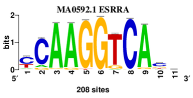	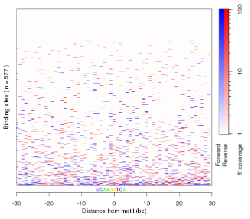	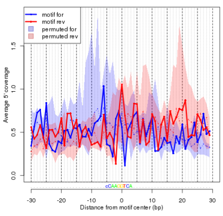	577	0.238691775089503
peaks-motifs de novo discovery **oligos_7nt_mkv5_m4** Most similar motif in JASPAR: **MEF2C**	-	-	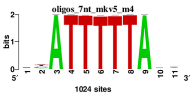	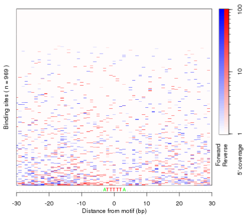	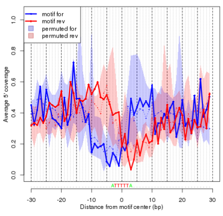	969	0.243590722448478
**MA0017.1** **NR2F1**	Zinc-coordinating	Hormone-nuclear Receptor	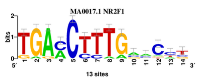	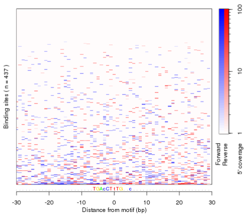	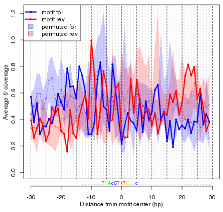	437	0.281681725787941
**MA0515.1** **Sox6**	Other Alpha-Helix	High Mobility Group (Box)	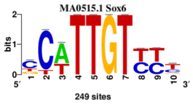	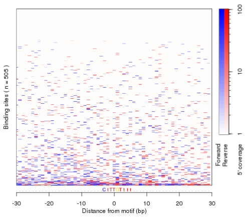	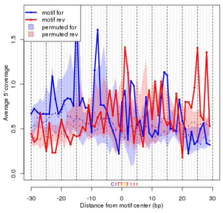	505	0.305547002673673
**MA0057.1** **MZF1_5-13**	Zinc-coordinating	BetaBetaAlpha-zinc finger	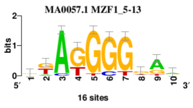	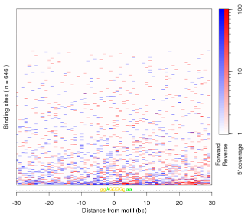	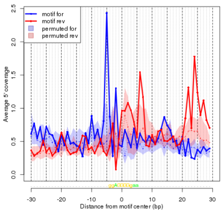	646	0.306799342388098

**MA0028.1**<br>**ELK1**	Winged Helix-Turn-Helix	Ets	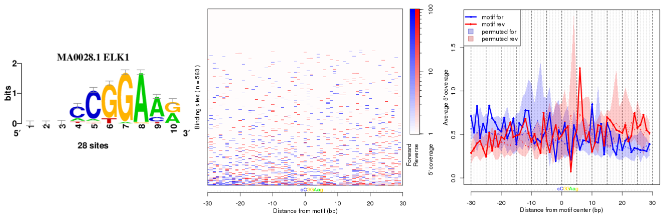	571	0.310441986171705
**MA0463.1**<br>**Bcl6**	Zinc-coordinating	BetaBetaAlpha-zinc Finger	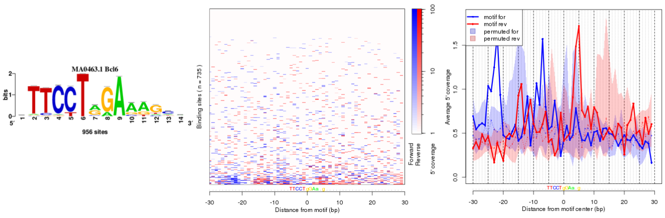	735	0.31666364287263
**MA0477.1**<br>**FOSL1**	Zipper-Type	Leucine-Zipper	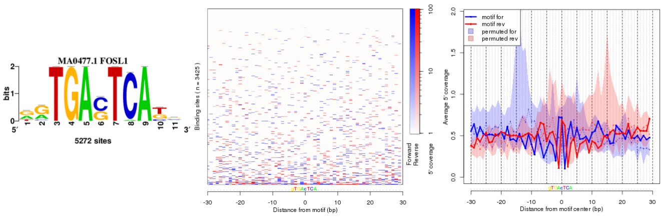	5015	0.324636258019575
**MA0597.1**<br>**THAP1**	Zinc-coordinating	THAP	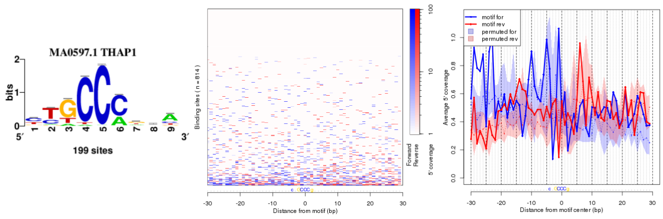	814	0.325377890601038
**MA0101.1**<br>**REL**	Ig-fold	Rel	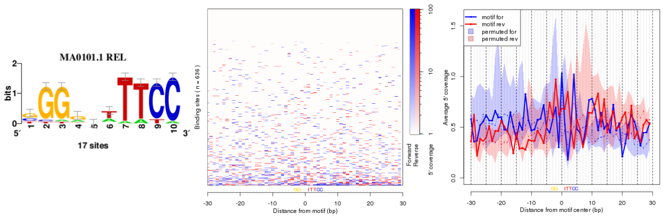	636	0.385105637457213

**MA0493.1**<br>**Klf1**	Zinc-coordinating	BetaBetaAlpha-zinc Finger	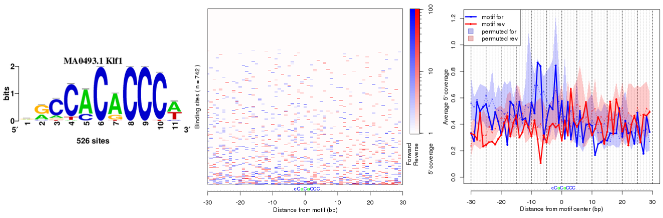	742	0.399090451754216
**MA0523.1**<br>**TCF7L2**	Other Alpha-Helix	High Mobility Group (Box)	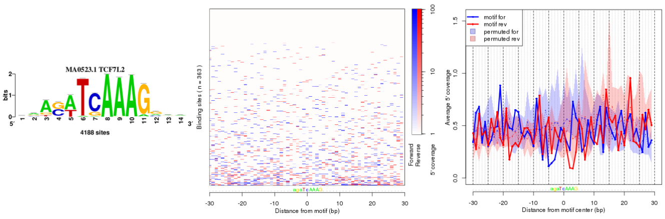	363	0.412150640780457
**MA0442.1**<br>**SOX10**	Other Alpha-Helix	High Mobility Group box (HMG)	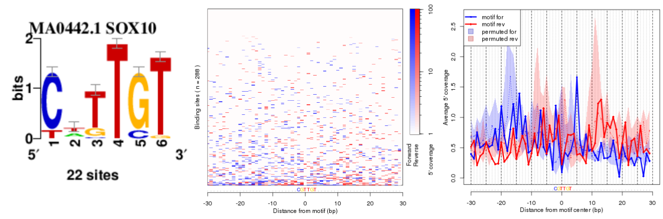	288	0.417102347174032
**MA0150.2**<br>**Nfe2l2**	Zipper-Type	Leucine Zipper	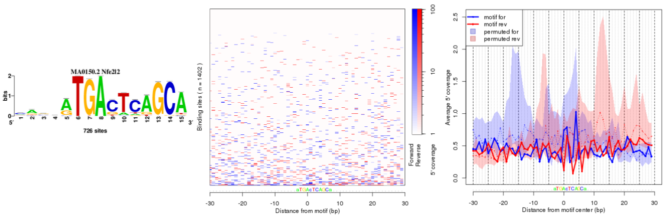	1402	0.443480347729068
**MA0513.1**<br>**SMAD2::SMAD3::SMAD4**	Zinc-coordinating	MH1	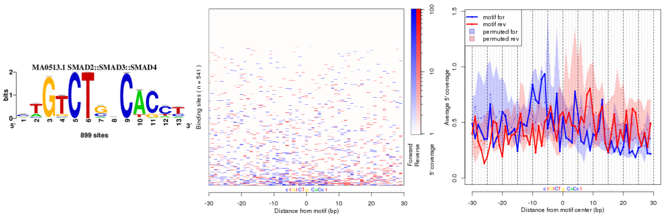	541	0.485095916035726

peaks-motifs de novo discovery **positions_7nt_m2** Most similar motif in JASPAR: **no match**	-	-	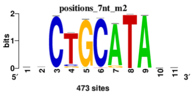 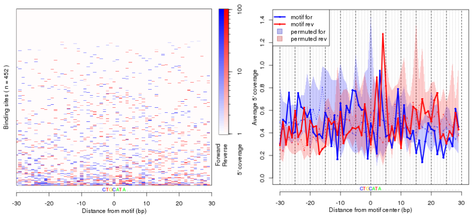	452	0.515927179563035
**MA0079.3** **SP1**	Zinc-coordinating	BetaBetaAlpha-zinc finger	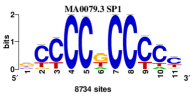 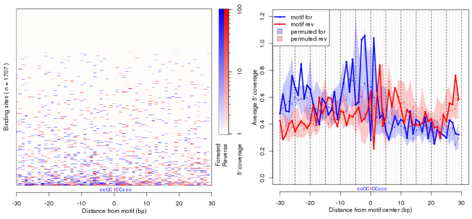	1707	0.517149504189253
**MA0105.3** **NFKB1**	Ig-fold	Rel	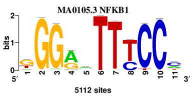 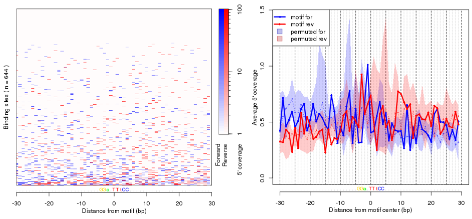	644	0.527108287271448
**MA0464.1** **Bhlhe40**	Zipper-Type	Helix-Loop-Helix	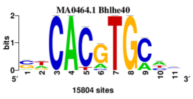 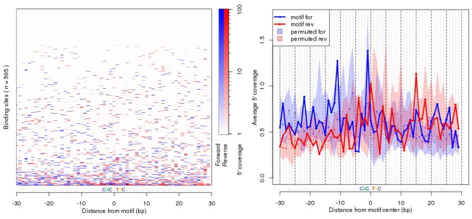	595	0.532700199868295
**MA0512.1** **Rxra**	Zinc-coordinating	Hormone-nuclear Receptor	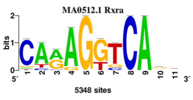 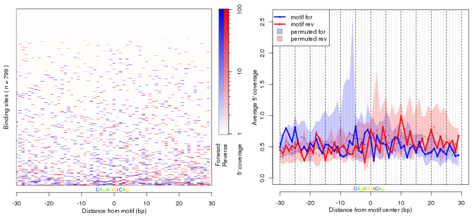	799	0.541750035712442
**MA0115.1** **NR1H2::RXRA**	Zinc-coordinating	Hormone-nuclear Receptor	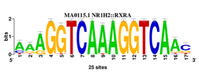 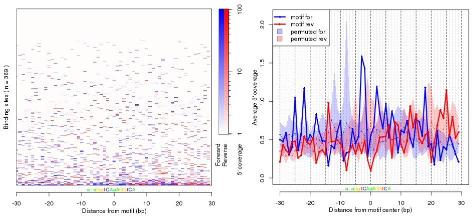	369	0.552248512190603

**MA0482.1** **Gata4**	Zinc-coordinating	GATA	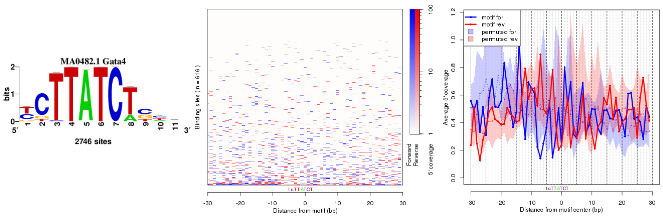	616	0.56010750038511
**MA0483.1** **Gfi1b**	Zinc-coordinating	BetaBetaAlpha-zinc Finger	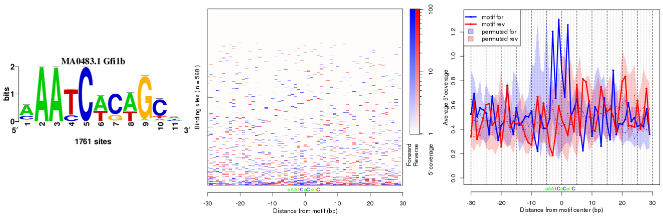	568	0.564330780472825
**MA0104.3** **Mycn**	Zipper-Type	Helix-Loop-Helix	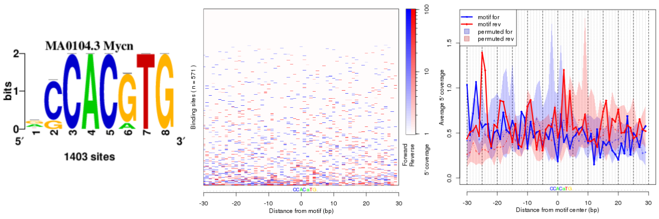	572	0.576523457820495
**MA0038.1** **Gfi1**	Zinc-coordinating	BetaBetaAlpha-zinc finger	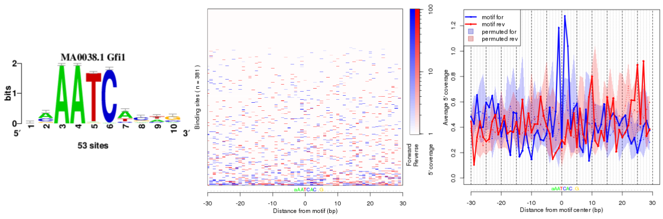	381	0.583659757961658
**MA0507.1** **POU2F2**	Helix-Turn-Helix	Homeodomain	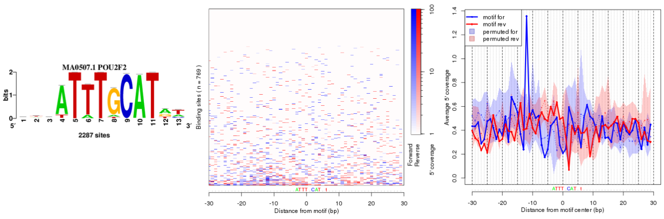	769	0.583777081645438
**MA0468.1** **DUX4**	Helix-Turn-Helix	Homeodomain	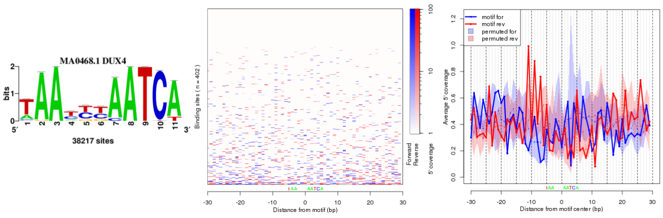	402	0.600835931416878

**MA0153.1** **HNF1B**	Helix-Turn-Helix	Homeo	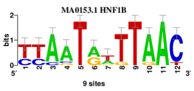	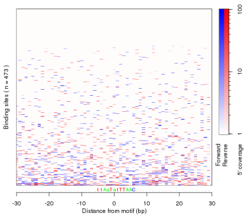	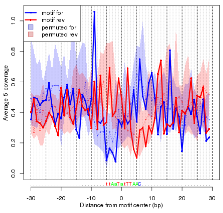	473	0.643142201245918
**MA0033.1** **FOXL1**	Winged Helix-Turn-Helix	Forkhead	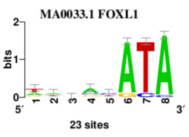	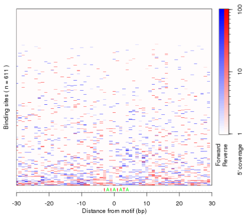	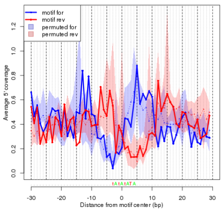	730	0.647988477296055
**MA0465.1** **CDX2**	Helix-Turn-Helix	Homeodomain	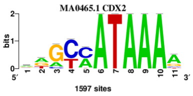	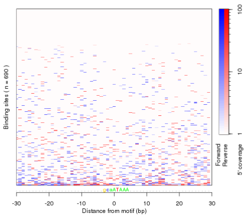	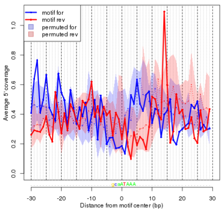	690	0.657356753274894
**MA0594.1** **Hoxa9**	Helix-Turn-Helix	Homeodomain	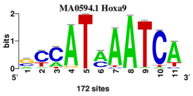	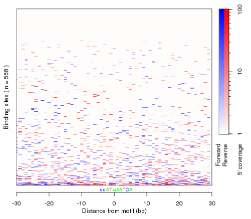	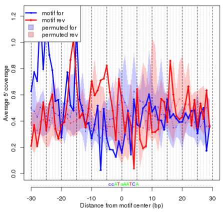	558	0.666764427087013
**MA0599.1** **KLF5**	Zinc-coordinating	BetaBetaAlpha-zinc Finger	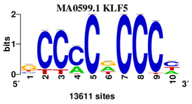	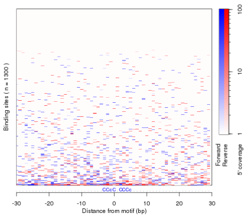	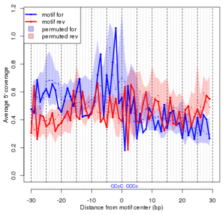	1300	0.666912198289041
**MA0595.1** **SREBF1**	Zipper-Type	Helix-Loop-Helix	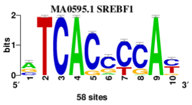	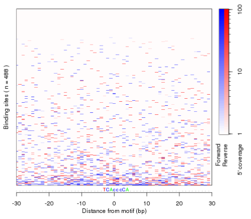	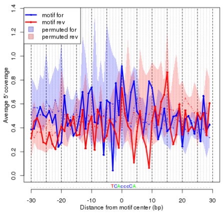	495	0.674104203643864

**MA0072.1**<br>**RORA_2**	Zinc-coordinating	Hormone-nuclear Receptor	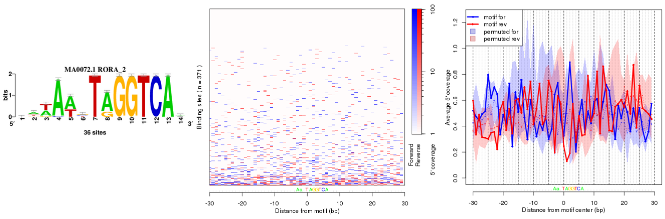	371	0.703708175046292
**MA0160.1**<br>**NR4A2**	Zinc-coordinating	Hormone-nuclear Receptor	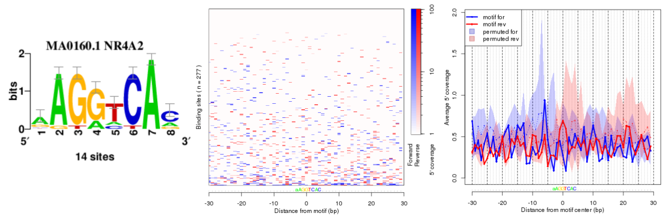	277	0.709508491544734
**MA0467.1**<br>**Crx**	Helix-Turn-Helix	Homeodomain	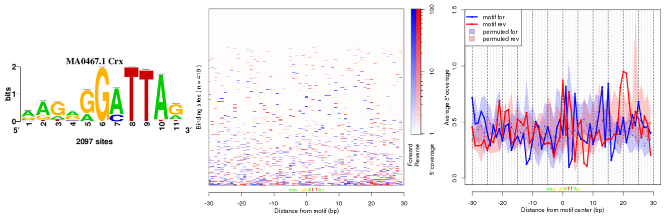	419	0.711395097620089
**MA0092.1**<br>**Hand1::Tcfe2a**	Zipper-Type	Helix-Loop-Helix	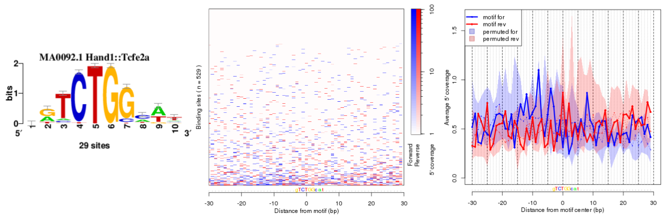	529	0.713697888023799
**MA0076.2**<br>**ELK4**	Winged Helix-Turn-Helix	Ets	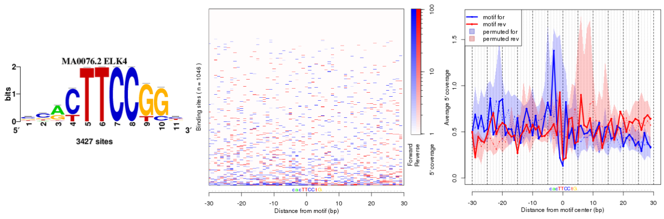	1046	0.716261043709482
**MA0514.1**<br>**Sox3**	Other Alpha-Helix	High Mobility Group (Box)	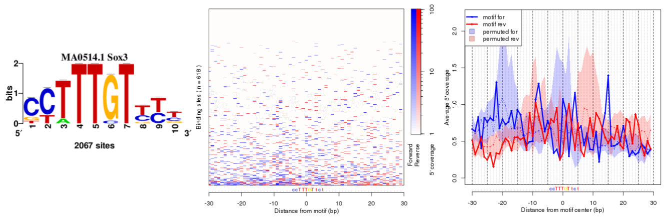	618	0.730206699366209

<table>
<tr><td></td><td></td><td></td><td></td><td></td><td></td></tr>
<tr><td>**MA0111.1**
Spz1</td><td>Other</td><td>Other</td><td>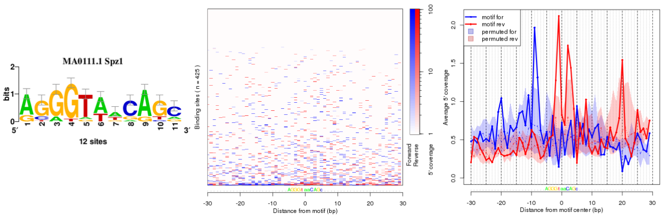
</td><td>425</td><td>0.741502397349324</td></tr>
<tr><td>**MA0485.1**
Hoxc9</td><td>Helix-Turn-Helix</td><td>Homeodomain</td><td>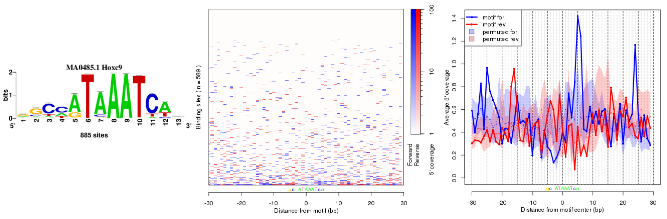
</td><td>589</td><td>0.748664759205412</td></tr>
<tr><td>peaks-motifs de novo discovery
oligos_6nt_mkv4_m3
Most similar motif in JASPAR:
ARID3A</td><td>-</td><td>-</td><td>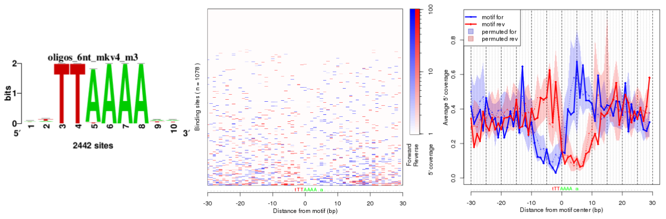
</td><td>1078</td><td>0.761944111509578</td></tr>
<tr><td>**MA0509.1**
Rfx1</td><td>Winged Helix-Turn-Helix</td><td>RFX</td><td>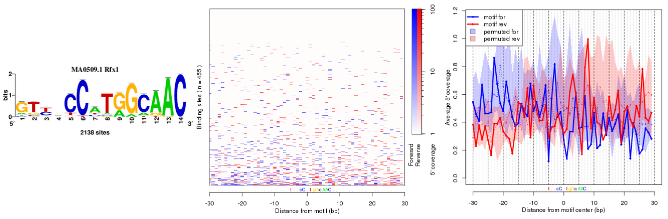
</td><td>489</td><td>0.789250961419959</td></tr>
<tr><td>**MA0014.2**
PAX5</td><td>Helix-Turn-Helix</td><td>Homeo</td><td>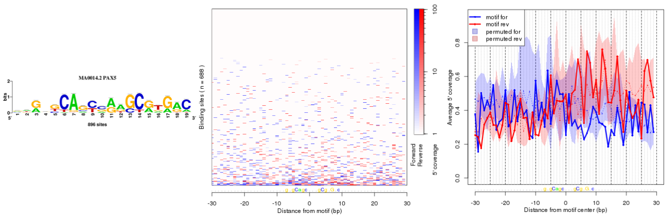
</td><td>688</td><td>0.791743042733607</td></tr>
<tr><td>**MA0146.2**
Zfx</td><td>Zinc-coordinating</td><td>BetaBetaAlpha-zinc finger</td><td>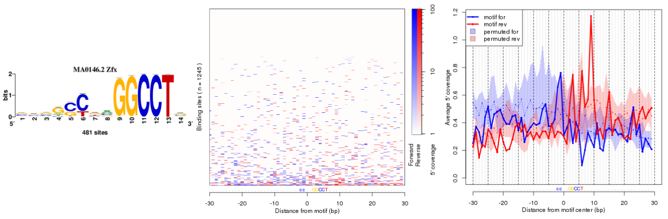
</td><td>1262</td><td>0.794886627817549</td></tr>
</table>

**MA0149.1**<br>**EWSR1-FLI1**	Winged Helix-Turn-Helix	Ets	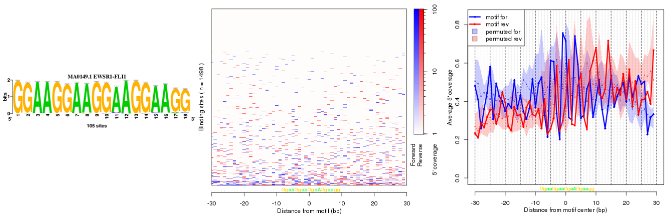	1498	0.808125456811774
**MA0122.1**<br>**Nkx3-2**	Helix-Turn-Helix	Homeo	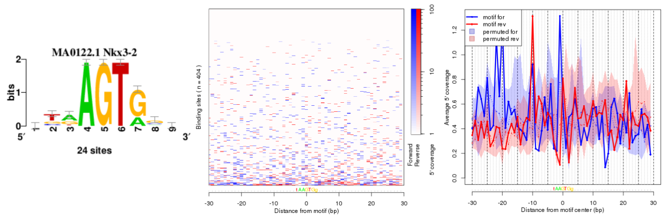	405	0.80930947246343
**MA0078.1**<br>**Sox17**	Other Alpha-Helix	High Mobility Group box (HMG)	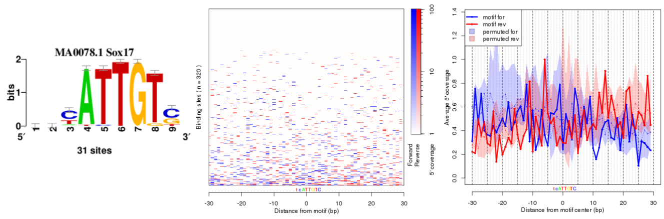	320	0.823459148473264
**MA0073.1**<br>**RREB1**	Zinc-coordinating	BetaBetaAlpha-zinc finger	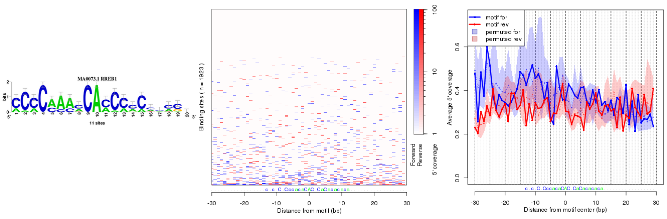	1923	0.845438635994866
**MA0083.2**<br>**SRF**	Other Alpha-Helix	MADS	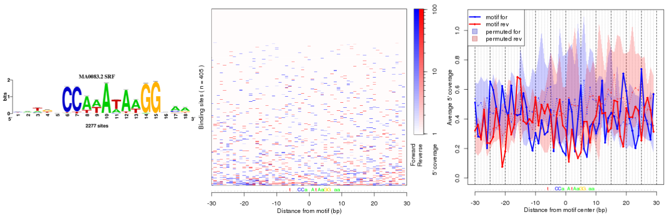	405	0.848626077821076
**MA0070.1**<br>**PBX1**	Helix-Turn-Helix	Homeo	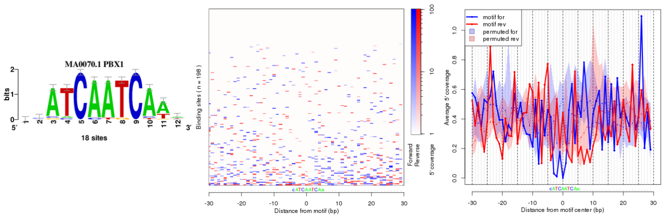	198	0.855691153261962

**MA0158.1** **HOXA5**	Helix-Turn-Helix	Homeo	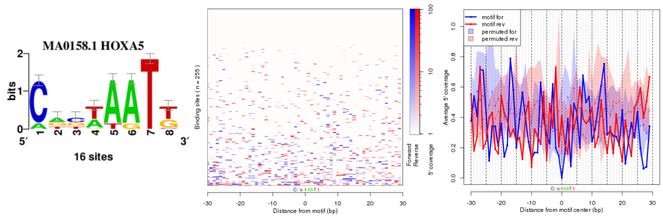	255	0.8581210250052
**MA0139.1** **CTCF**	Zinc-coordinating	BetaBetaAlpha-zinc finger	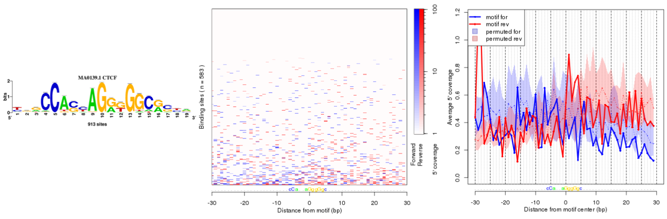	583	0.859133091691821
peaks-motifs de novo discovery **oligos_6nt_mkv4_m1** Most similar motif in JASPAR: **JUND**	-	-	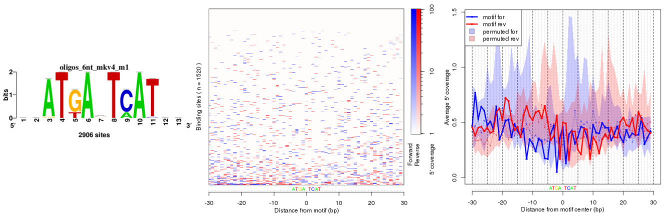	3040	0.863117394968998
**MA0470.1** **E2F4**	Winged Helix-Turn-Helix	E2F	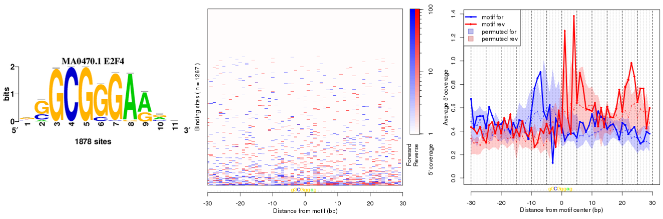	1267	0.863299438959019
**MA0508.1** **PRDM1**	Zinc-coordinating	BetaBetaAlpha-zinc Finger	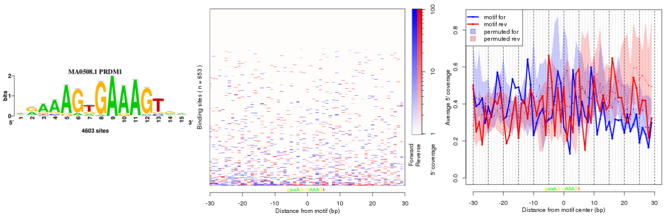	653	0.863480964826342
**MA0161.1** **NFIC**	Other	NFI CCAAT-binding	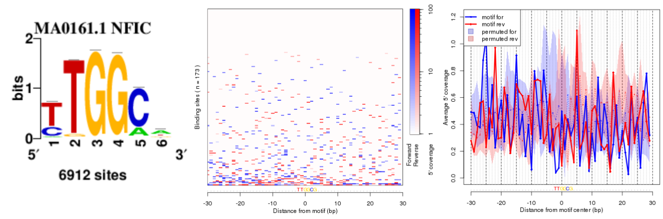	173	0.877789793949493

**MA0495.1**<br>**MAFF**	Zipper-Type	Leucine-Zipper	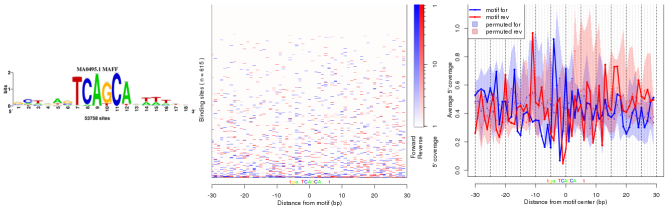	615	0.882580996641004
**MA0029.1**<br>**Mecom**	Zinc-coordinating	BetaBetaAlpha-zinc finger	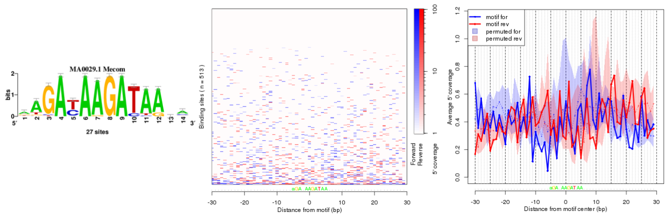	513	0.883338852617938
**MA0501.1**<br>**NFE2::MAF**	Zipper-Type	Leucine-Zipper	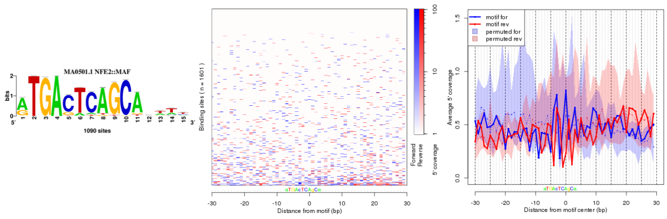	1601	0.883953638914765
**MA0051.1**<br>**IRF2**	Winged Helix-Turn-Helix	IRF	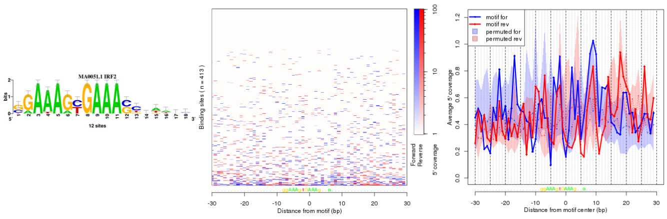	413	0.889533529472367
**MA0163.1**<br>**PLAG1**	Zinc-coordinating	BetaBetaAlpha-zinc finger	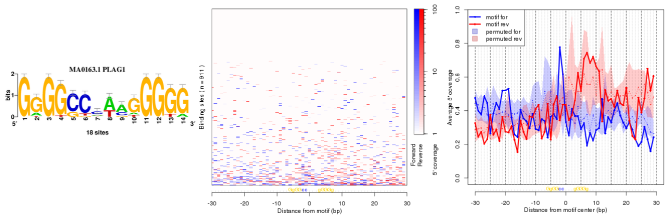	911	0.896877658723152
**MA0140.2**<br>**TAL1::GATA1**	Zipper-Type	Helix-Loop-Helix	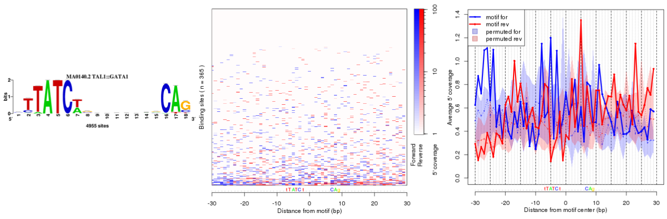	365	0.904121967561927
**MA0497.1**<br>**MEF2C**	Other Alpha-Helix	MADS	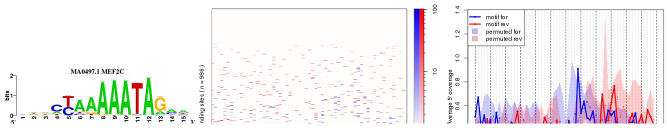	986	0.904286910638692

			2209 sites 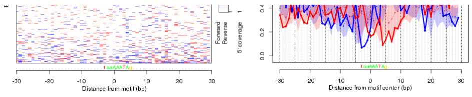		
**MA0046.1** **HNF1A**	Helix-Turn-Helix	Homeo	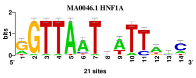 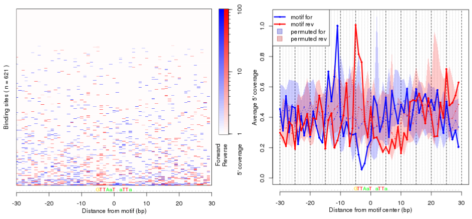	632	0.921889193228842
**MA0527.1** **ZBTB33**	Zinc-coordinating	BetaBetaAlpha-zinc Finger	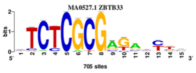 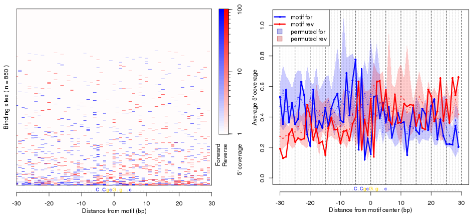	850	0.92427942278078
**MA0133.1** **BRCA1**	Other	Other	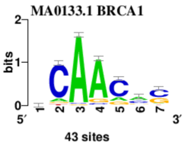 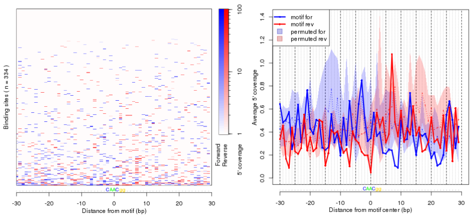	334	0.924398422853223
**MA0142.1** **Pou5f1::Sox2**	Helix-Turn-Helix	Homeo	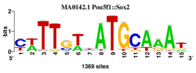 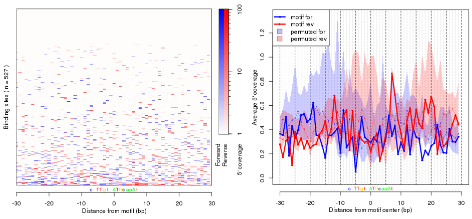	527	0.93661035863463
**MA0506.1** **NRF1**	Other	NRF	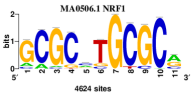 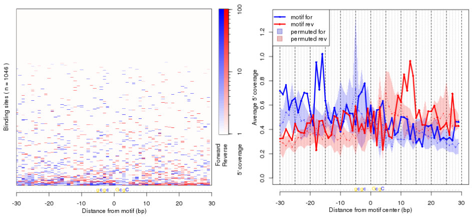	1046	0.941292516855448
**MA0093.2** **USF1**	Zipper-Type	Helix-Loop-Helix		563	0.9419500865533

			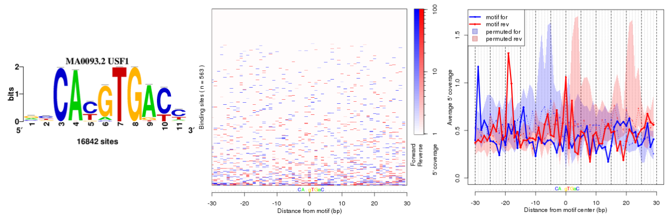		
**MA0600.1**<br>**RFX2**	Winged Helix-Turn-Helix	RFX	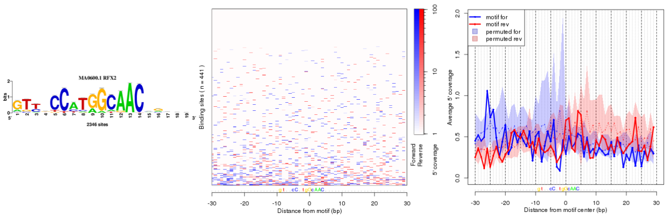	441	0.945975258870561
**MA0050.2**<br>**IRF1**	Winged Helix-Turn-Helix	IRF	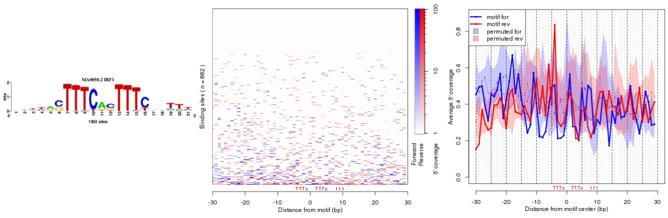	882	0.949553842666775
**MA0018.2**<br>**CREB1**	Zipper-Type	Leucine Zipper	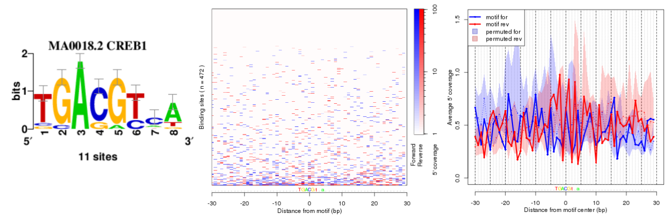	532	0.95830203977225
**MA0517.1**<br>**STAT2::STAT1**	Other	STAT	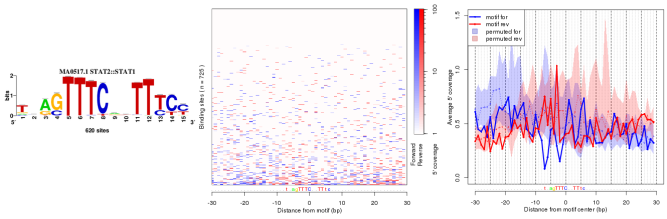	725	0.964477590507233
**MA0077.1**<br>**SOX9**	Other Alpha-Helix	High Mobility Group box (HMG)	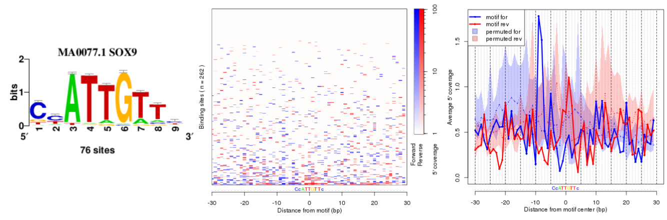	262	0.964662644063331
**MA0024.2**	Winged Helix-Turn-	E2F	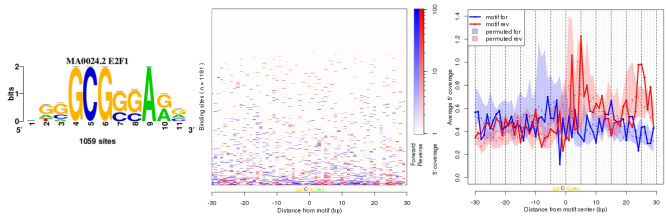	1181	0.974001383288818

**E2F1**	[illegible]				
**MA0117.1** **Mafb**	Zipper-Type	Leucine Zipper	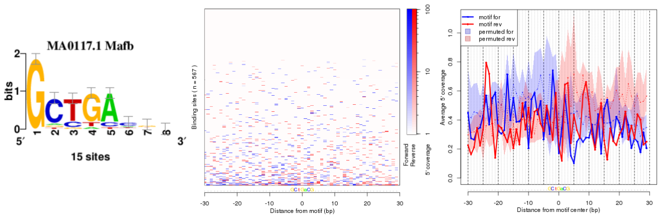	583	0.982444028708434
**MA0100.2** **Myb**	Helix-Turn-Helix	Myb	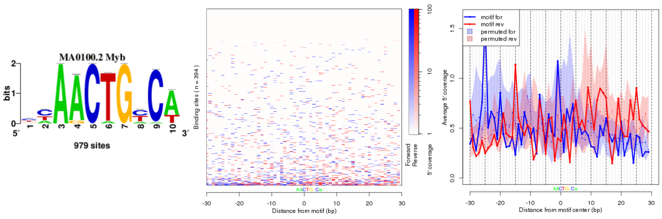	394	0.985738012519309
**MA0095.2** **YY1**	Zinc-coordinating	BetaBetaAlpha-zinc finger	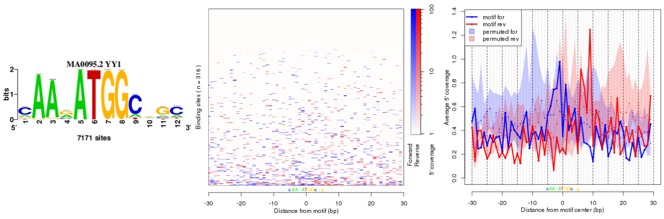	316	0.987289917782374
**MA0478.1** **FOSL2**	Zipper-Type	Leucine-Zipper	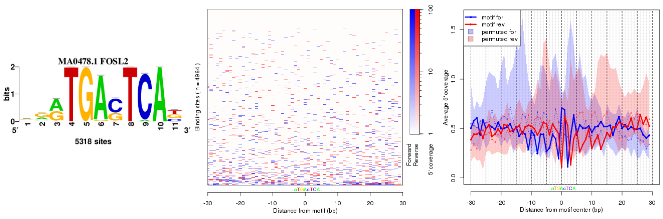	4964	0.98742546672963
**MA0125.1** **Nobox**	Helix-Turn-Helix	Homeo	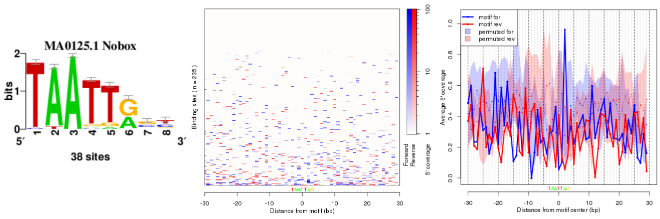	235	0.994028227250405
**MA0498.1** [illegible]	Helix-Turn-Helix	Homeodomain	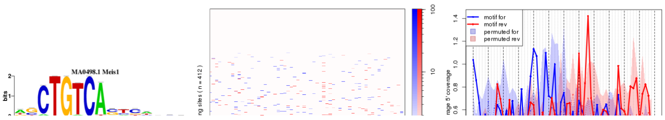	412	0.996640526982052

[illegible]				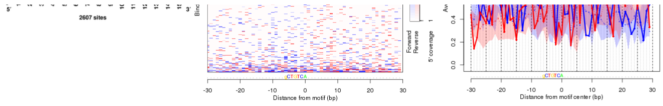		
**MA0526.1**<br>**USF2**	Zipper-Type	Helix-Loop-Helix	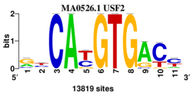	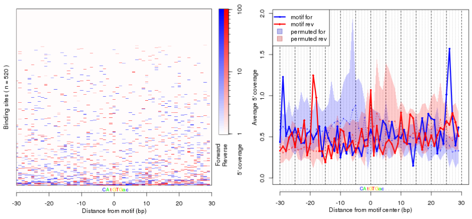	520	0.996998228915274
**MA0135.1**<br>**Lhx3**	Helix-Turn-Helix	Homeo	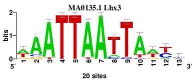	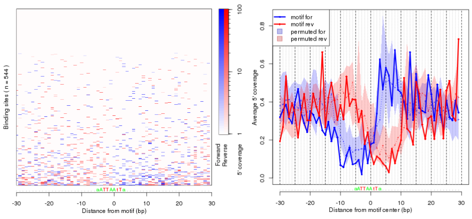	544	0.997476971052255
peaks-motifs de novo discovery<br>**positions_6nt_m4**<br>Most similar motif in JASPAR:<br>**NRF1**	-	-	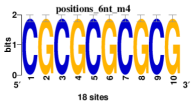	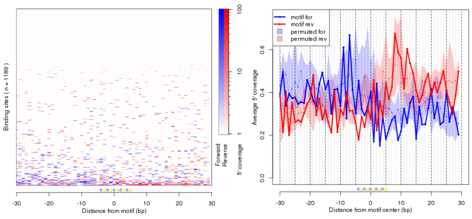	2378	0.9975543969116
**MA0069.1**<br>**Pax6**	Helix-Turn-Helix	Homeo	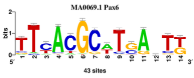	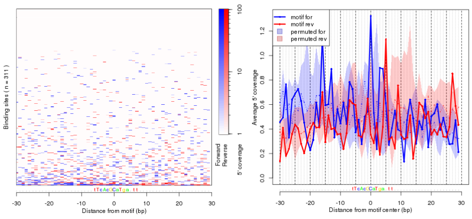	311	0.998430762197329
**MA0472.1**<br>**EGR2**	Zinc-coordinating	BetaBetaAlpha-zinc Finger	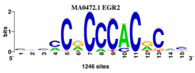	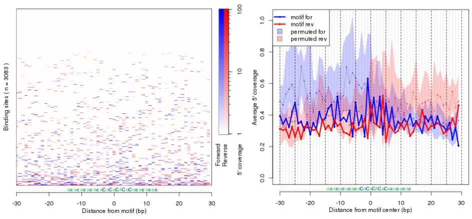	3083	0.998605015219158
**MA0502.1**<br>**NFYB**	Other Alpha-Helix	NF-Y CCAAT-Binding	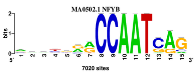	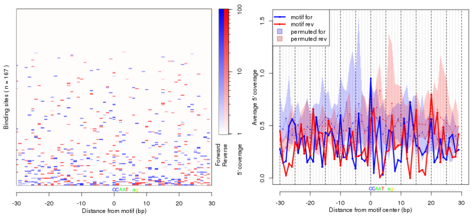	167	0.999493101828354

**MA0124.1**<br>**NKX3-1**	Helix-Turn-Helix	Homeo	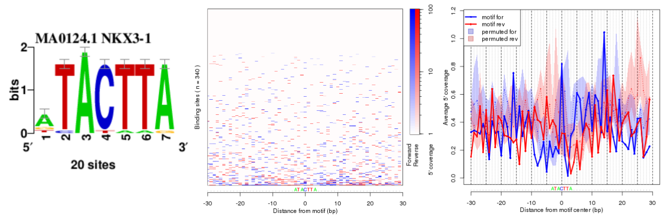	340	0.999726770693167
**MA0009.1**<br>**T**	Beta-Hairpin-Ribbon	T	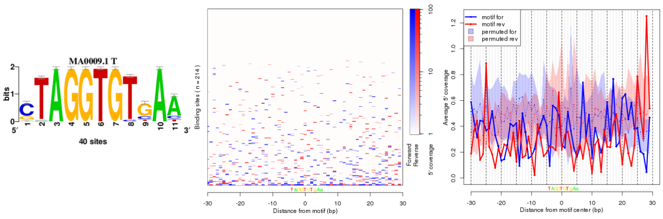	214	0.999940710134937
**MA0087.1**<br>**Sox5**	Other Alpha-Helix	High Mobility Group box (HMG)	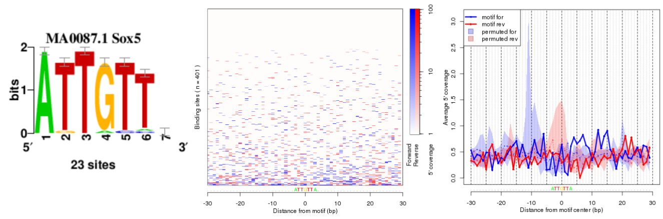	401	0.999993250772955
**MA0060.2**<br>**NFYA**	Other Alpha-Helix	NFY CCAAT-binding	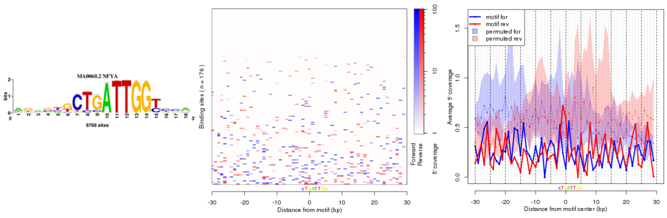	176	0.999996203486472
**MA0103.2**<br>**ZEB1**	Zinc-coordinating	BetaBetaAlpha-zinc finger	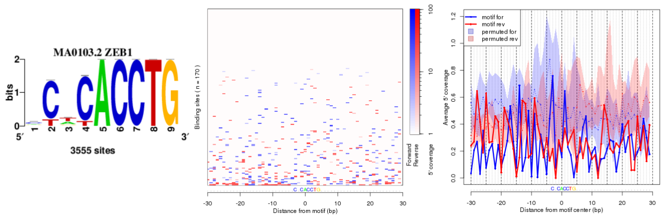	170	0.999999977614683
**MA0063.1**<br>**Nkx2-5**	Helix-Turn-Helix	Homeo	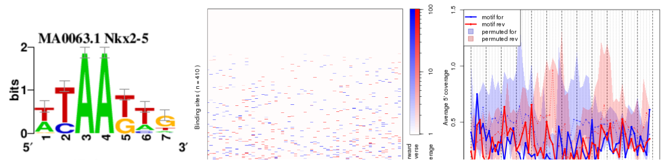	410	0.999999979579607

			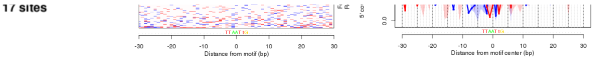		
**MA0075.1** **Prrx2**	Helix-Turn-Helix	Homeo	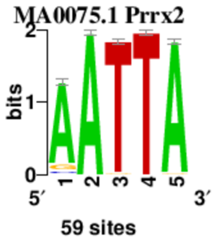	0	1
**MA0089.1** **NFE2L1::MafG**	Zipper-Type	Leucine Zipper	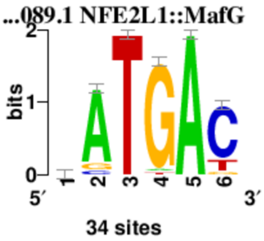	0	1
**MA0130.1** **ZNF354C**	Zinc-coordinating	BetaBetaAlpha-zinc finger	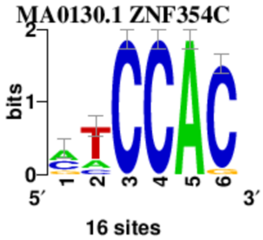	0	1
**MA0132.1** **Pdx1**	Helix-Turn-Helix	Homeo	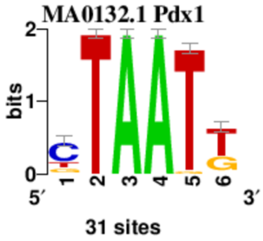	0	1
**MA0151.1** **ARID3A**	Helix-Turn-Helix	Arid	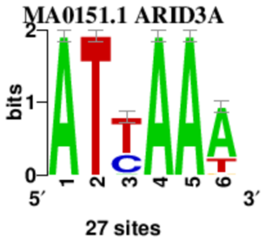	0	1
**MA0152.1** **NFATC2**	Ig-fold	Rel	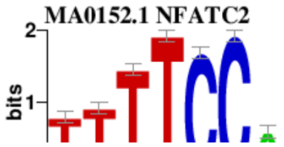	0	1

			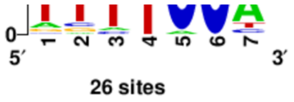		
**MA0164.1** **Nr2e3**	Zinc-coordinating	Hormone-nuclear Receptor	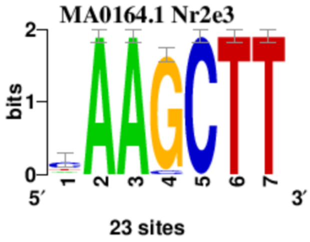	0	1
**MA0099.2** **JUN::FOS**	Zipper-Type	Leucine Zipper	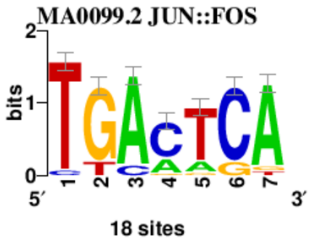	0	1
**MA0489.1** **JUN (var.2)**	Zipper-Type	Leucine-Zipper	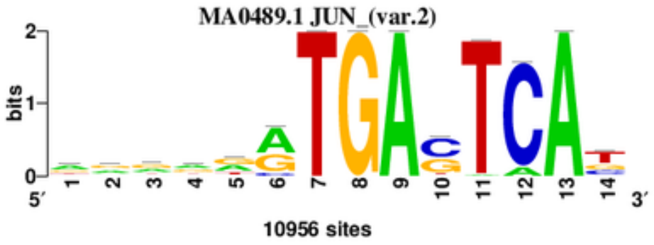	0	1
**MA0492.1** **JUND (var.2)**	Zipper-Type	Leucine-Zipper	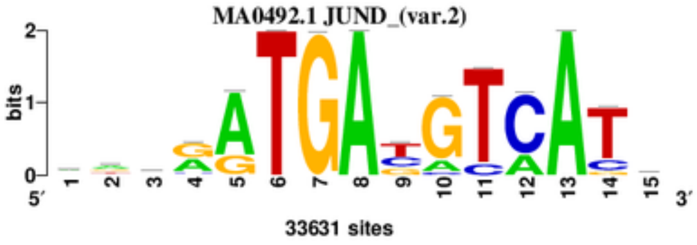	0	1
**MA0496.1** **MAFK**	Zipper-Type	Leucine-Zipper	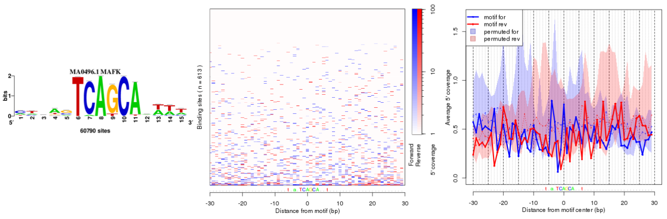	613	1
**MA0503.1** **Nkx2-5 (var.2)**	Helix-Turn-Helix	Homeodomain	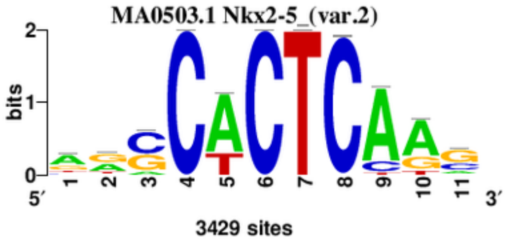	0	1
