# Supplementary material for: ChIP-exo signal associated with DNA-binding motifs provides insight into the genomic binding of the glucocorticoid receptor and cooperating transcription factors
Source: Genome Res. 2015 Jun;25(6):825–35. doi: 10.1101/gr.185157.114 (PMC4448679; doi:10.1101/gr.185157.114)
Supplement: Supplemental Material [file supp_gr.185157.114_Supplemental_Data_3.pdf]

Supp Data 3 : TTCC Motif comparison and structural alignment

Starick, Ibn-Salem, Jurk et al *ChIP-exo signal associated with DNA-binding motifs provide insights into the genomic binding of the glucocorticoid receptor and cooperating transcription factors.*

One-to-n motif alignment with RSAT compare-matrices (www.rsat.eu); reference motif: TTCC ; Database: Jaspar Vertebrates ; 25 matrices
4 classes: Zinc-coordinating,Winged Helix-Turn-Helix,Helix-Turn-Helix,Ig-fold
6 families : BetaBetaAlpha-zinc finger, E2f, Ets,homeo,Rel,Stat

Matrix name	Class	Family	Aligned logos	PDB structure	Structural alignment result
TTCC motif query	-	-	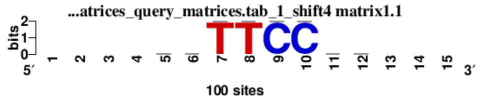		
MA0056.1_rc_shift7 (MZF1_1-4_rc)	Zinc-coordinating	BetaBetaAlpha-zinc finger	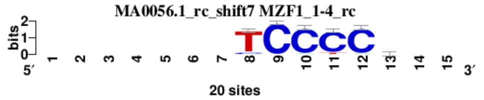	no structure	
MA0471.1_rc_shift4 (E2F6_rc)	Winged Helix-Turn-Helix	E2F	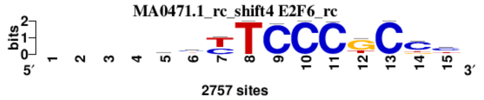		
MA0470.1_rc_shift4 (E2F4_rc)	Winged Helix-Turn-Helix	E2F	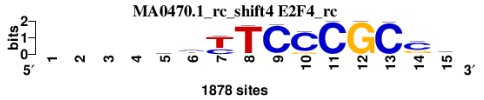		
MA0081.1_rc_shift6 (SPIB_rc)	Winged Helix-Turn-Helix	Ets	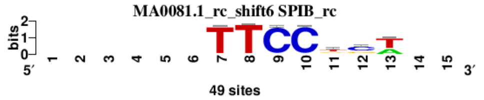		
MA0136.1_shift3 (ELF5)	Winged Helix-Turn-Helix	Ets	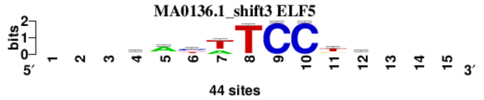		
MA0076.2_shift2 (ELK4)	Winged Helix-Turn-Helix	Ets	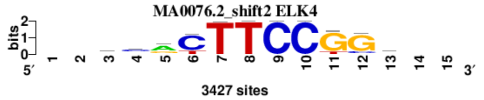		
MA0028.1_rc_shift5 (ELK1_rc)	Winged Helix-Turn-Helix	Ets	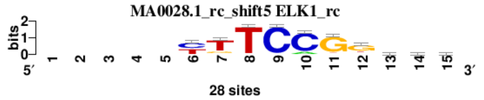	1DUX	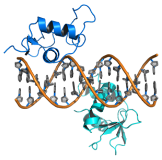
MA0598.1_shift4 (EHF)	Winged Helix-Turn-Helix	Ets	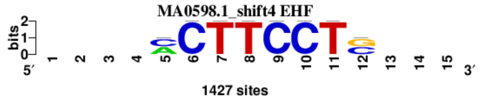		
MA0475.1_rc_shift2 (FLI1_rc)	Winged Helix-Turn-Helix	Ets	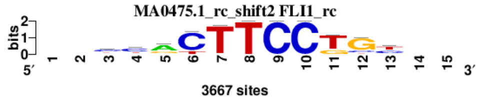		
MA0474.1_rc_shift2 (Erg_rc)	Winged Helix-Turn-Helix	Ets	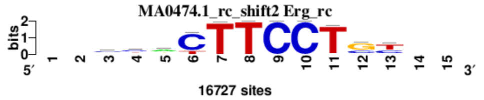		
MA0080.3_rc_shift2 (Spi1_rc)	Winged Helix-Turn-Helix	Ets	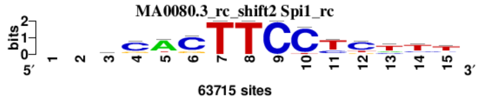		
MA0156.1_rc_shift4 (FEV_rc)	Winged Helix-Turn-Helix	Ets	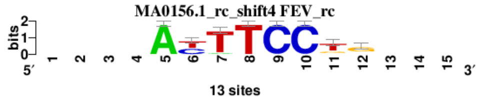		
MA0473.1_rc_shift3 (ELF1_rc)	Winged Helix-Turn-Helix	Ets	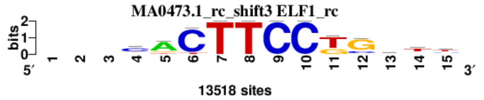		
MA0062.2_rc_shift1 (GABPA_rc)	Winged Helix-Turn-Helix	Ets	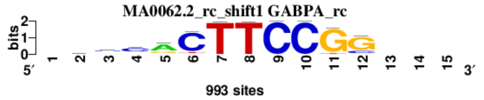		
MA0098.2_shift1 (Ets1)	Winged Helix-Turn-Helix	Ets	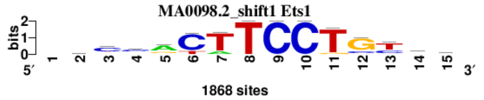	1K79	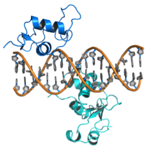

MA0090.1_shift2 (TEAD1)	Helix-Turn-Helix	Homeo	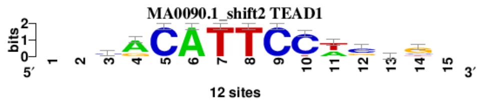	2HZD	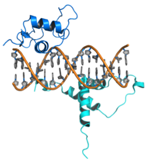
MA0101.1_shift0 (REL)	Ig-fold	Rel	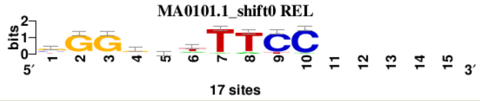		
MA0152.1_shift4 (NFATC2)	Ig-fold	Rel	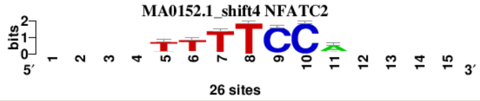		
MA0107.1_shift0 (RELA)	Ig-fold	Rel	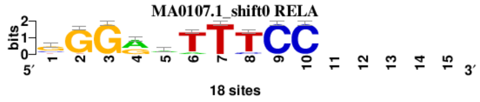		
MA0105.3_shift0 (NFKB1)	Ig-fold	Rel	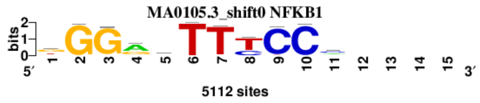	1SVC (p50)<br>2O61 (p65)	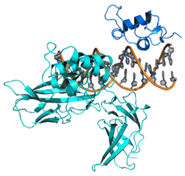 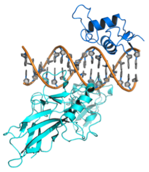
MA0519.1_shift4 (Stat5a::Stat5b)	Ig-fold (other)	STAT	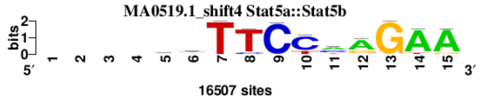		
MA0520.1_shift3 (Stat6)	Ig-fold (other)	STAT	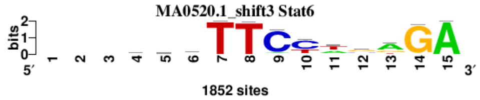		
MA0518.1_rc_shift2 (Stat4_rc)	Ig-fold (other)	STAT	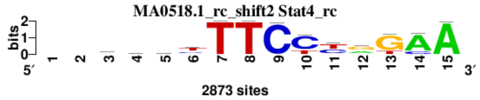		
MA0137.3_rc_shift5 (STAT1_rc)	Ig-fold	Stat	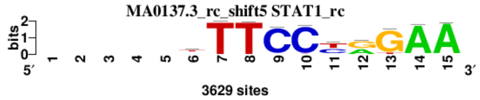	1BF5	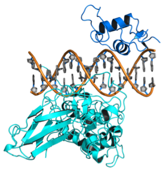
MA0144.2_rc_shift5 (STAT3_rc)	Ig-fold	Stat	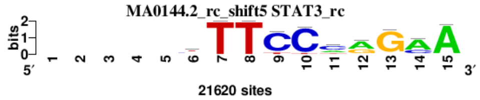		
