# Supplementary material for: ChIP-exo signal associated with DNA-binding motifs provides insight into the genomic binding of the glucocorticoid receptor and cooperating transcription factors
Source: Genome Res. 2015 Jun;25(6):825–35. doi: 10.1101/gr.185157.114 (PMC4448679; doi:10.1101/gr.185157.114)
Supplement: Supplemental Material [file supp_gr.185157.114_Supplemental_Material.pdf]

Supplemental Material

ChIP-exo signal associated with DNA-binding motifs provide insights into the genomic binding of the glucocorticoid receptor and cooperating transcription factors

Stephan R. Starick[1]*, Jonas Ibn-Salem[1]*, Marcel Jurk[1]*, Céline Hernandez[2], Michael I. Love[1], Ho-Ryun Chung[1], Martin Vingron[1], Morgane Thomas-Chollier[2#], Sebastiaan H. Meijsing[1#]

[1] Max Planck Institute for Molecular Genetics, Ihnestrasse 63-73 14195, Berlin, Germany.
[2] Institut de Biologie de l'Ecole Normale Supérieure, Institut National de la Santé et de la Recherche Médicale, U1024, Centre National de la Recherche Scientifique, Unité Mixte de Recherche 8197, F-75005 Paris, France.

Contact information:

Morgane Thomas-Chollier
Institut de Biologie de l'Ecole Normale Supérieure
46 rue d'Ulm
75230, Paris, France
Email: mthomas@biologie.ens.fr

Sebastiaan H. Meijsing
Max Planck Institute for Molecular Genetics,
Ihnestrasse 63-73
14195, Berlin, Germany
Tel: +49-30-84131176
Email: meijsing@molgen.mpg.de

Additional footnotes:
* co-first author
[#] co-corresponding author

This PDF includes:
- Supplemental Figures
- Supplemental Experimental Procedures
- Supplemental References

Supplemental figures:

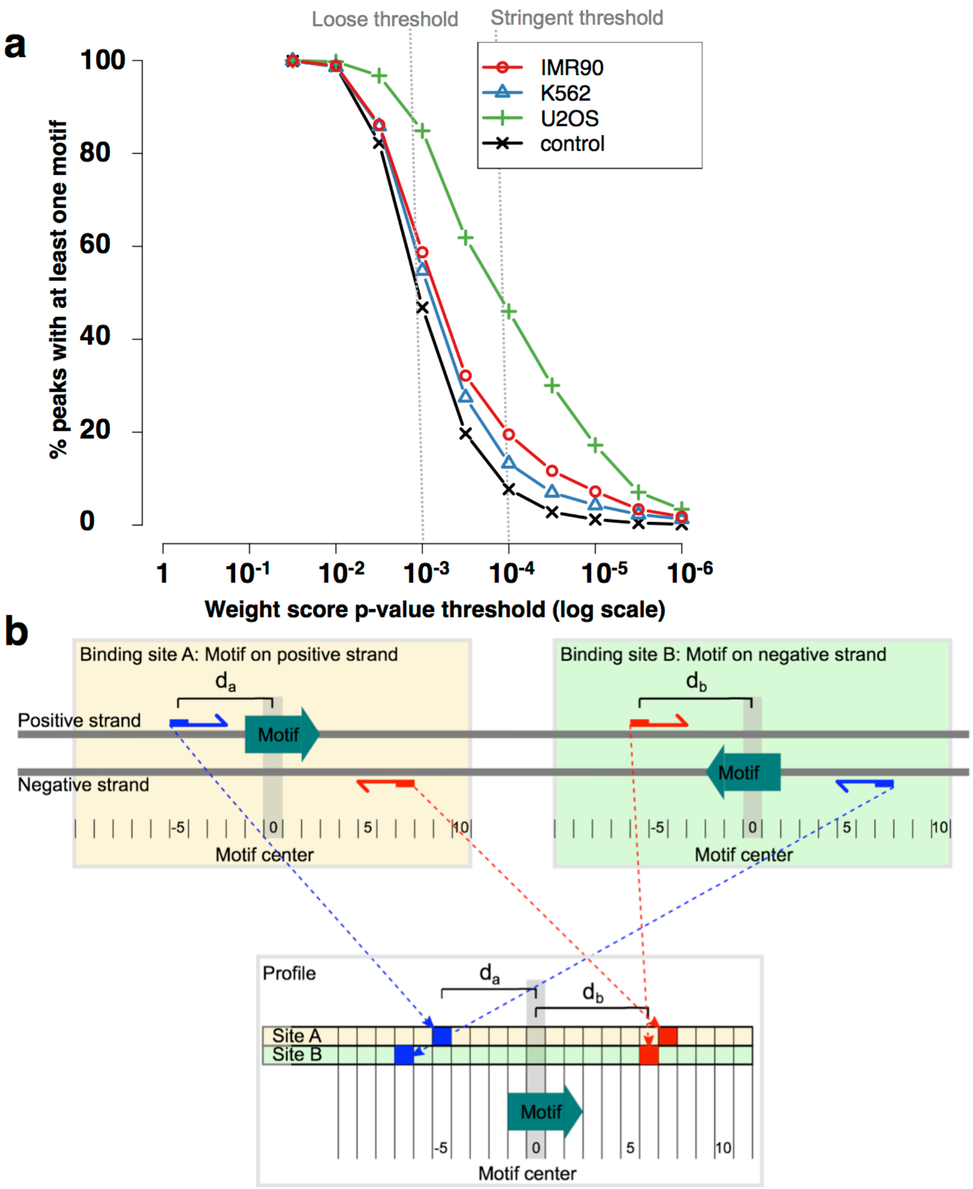

Figure S1. Fraction of GR-bound regions containing a GBS in different cell types. (a) Percentage of ChIP-seq peaks with at least one GBS motif (JASPAR MA0113.2) match is plotted against the p-value threshold used to scan for motif hits, for three cell lines. (b) Schematic diagram illustrating ExoProfiler's strand-sensitivity. For motifs matching on the negative strand (green shaded box), the whole region is reverse-complemented and the motif's center is aligned to the forward motifs. Reads initially mapped to the positive strand are thus treated as negative strand (here colored in red).

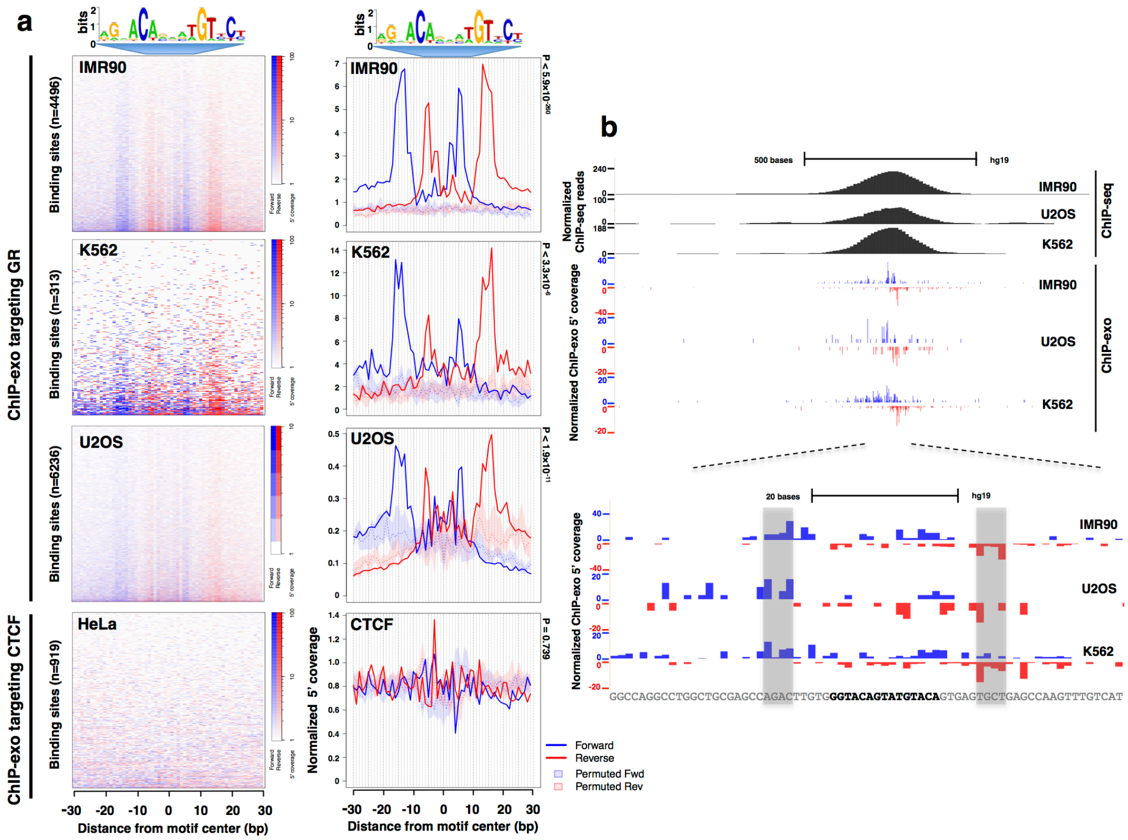

Figure S2. Comparison of the GBS ChIP-exo footprint between cell lines. (a) ChIP-exo coverage heatmaps for sequences matching the GBS motif (JASPAR MA0113.2) and footprint profiles for GR ChIP-exo in three cell lines and for CTCF ChIP-exo in HeLA cells are shown. (b) Example of ChIP-seq and ChIP-exo coverage at the *ZBTB16* locus, at a region bound by GR in all three cell lines examined, shows similar coverage around a sequence resembling the GBS motif (highlighted in bold).

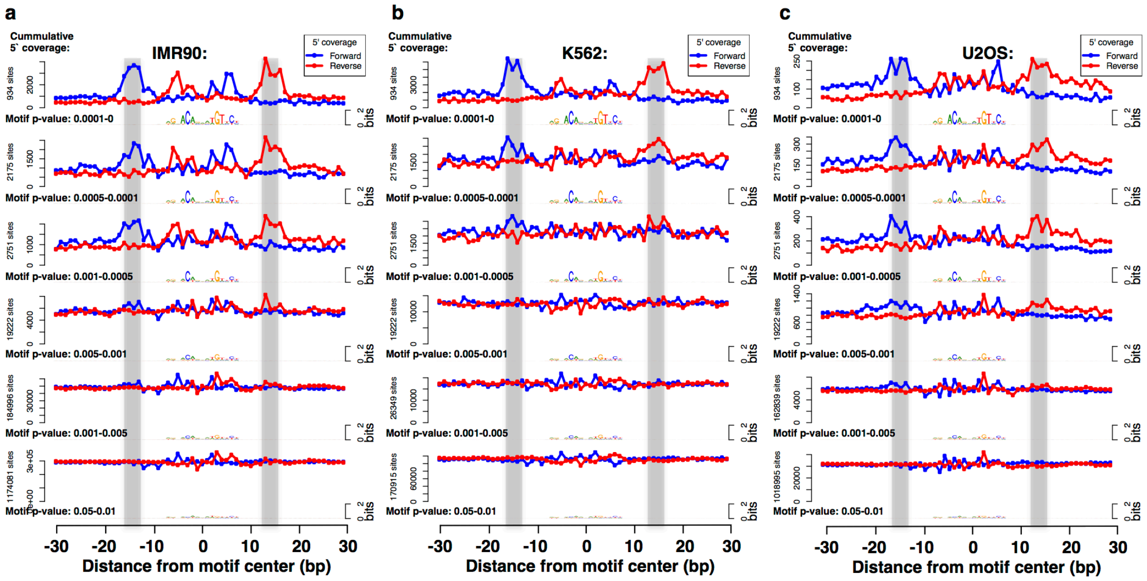

Figure S3. GR ChIP-exo footprint profiles for sequences matching the GBS consensus motif (JASPAR MA0113.2) at different motif p-value thresholds. Footprint profiles for a random sampling of equal numbers of motif-matches within ChIP-seq peaks for each cell line and p-value bin for (a) IMR90 (b) K562 and (c) U2OS cells. For each p-value threshold, the lowest number of sites (K562) is chosen as the number of motif matches to sample in the other cell lines.

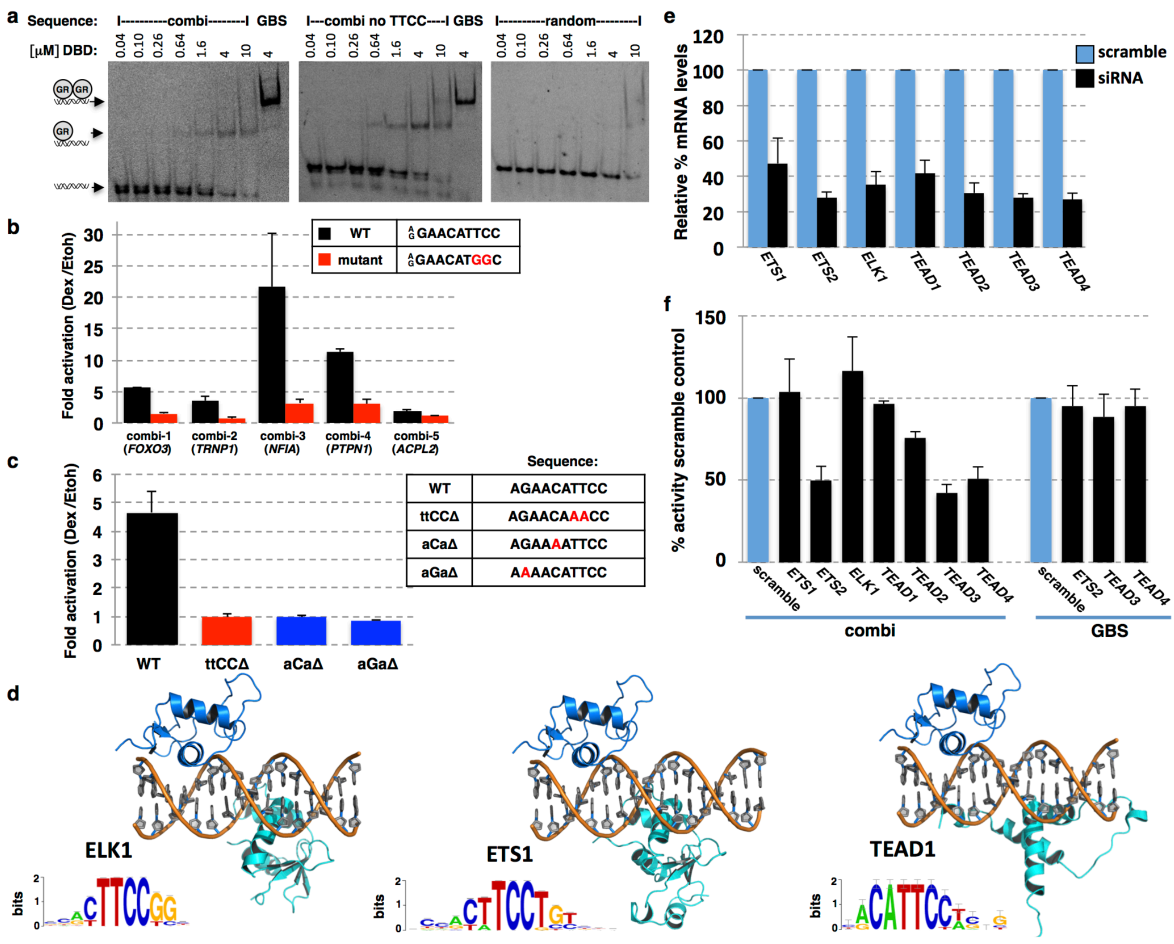

Figure S4. Functional and structural characterization of the combi motif. (a) EMSA showing left: GR DBD binding to combi sequence; middle: combi sequence with mutated TTCC and right: randomized sequence. Compared to the combi motif, the GBS shows a higher shifted band indicative of dimeric GR binding. (b) Genomic fragments near GR-target genes with sequences matching the combi motif, or a mutated version, were cloned upstream of a minimal promoter driving luciferase expression. Fold induction ± SEM upon treatment with 1 μM dexamethasone (dex) for wild-type and mutated reporters in U2OS cells is shown (n=3). (c) Transcriptional activity of luciferase reporters containing a minimal promoter together with three copies of the combi motif or mutant versions as indicated. Fold induction ± SEM (n≥3) in IMR90 cells upon treatment with 1 μM dex is shown. (d) Structural alignment of combined binding of a GR monomer and candidate "partnering" proteins ELK1 (left: PDB 1DUX), ETS1 (middle: PDB 1K79) and TEAD1 (right: PDB 2HZD). (e) Efficacy of siRNA knockdown of candidate partnering factors in U2OS cells. RNA levels two days after transfection with dsiRNAs as indicated were quantified by qPCR. Percentage relative to scramble control ± SEM (n≥3) is shown. (f) Effect of siRNA knockdown of genes as indicated on the activity of the combi motif. U2OS cells were transfected with dsiRNAs prior to transfection with the combi or the GBS reporter CGT (Meijsing et al. 2009), which contains three copies of a GBS motif driving expression of a luciferase reporter. Activities upon treatment with 1 μM dex are shown as percentage of that observed for the scramble control ± SEM (n≥3).

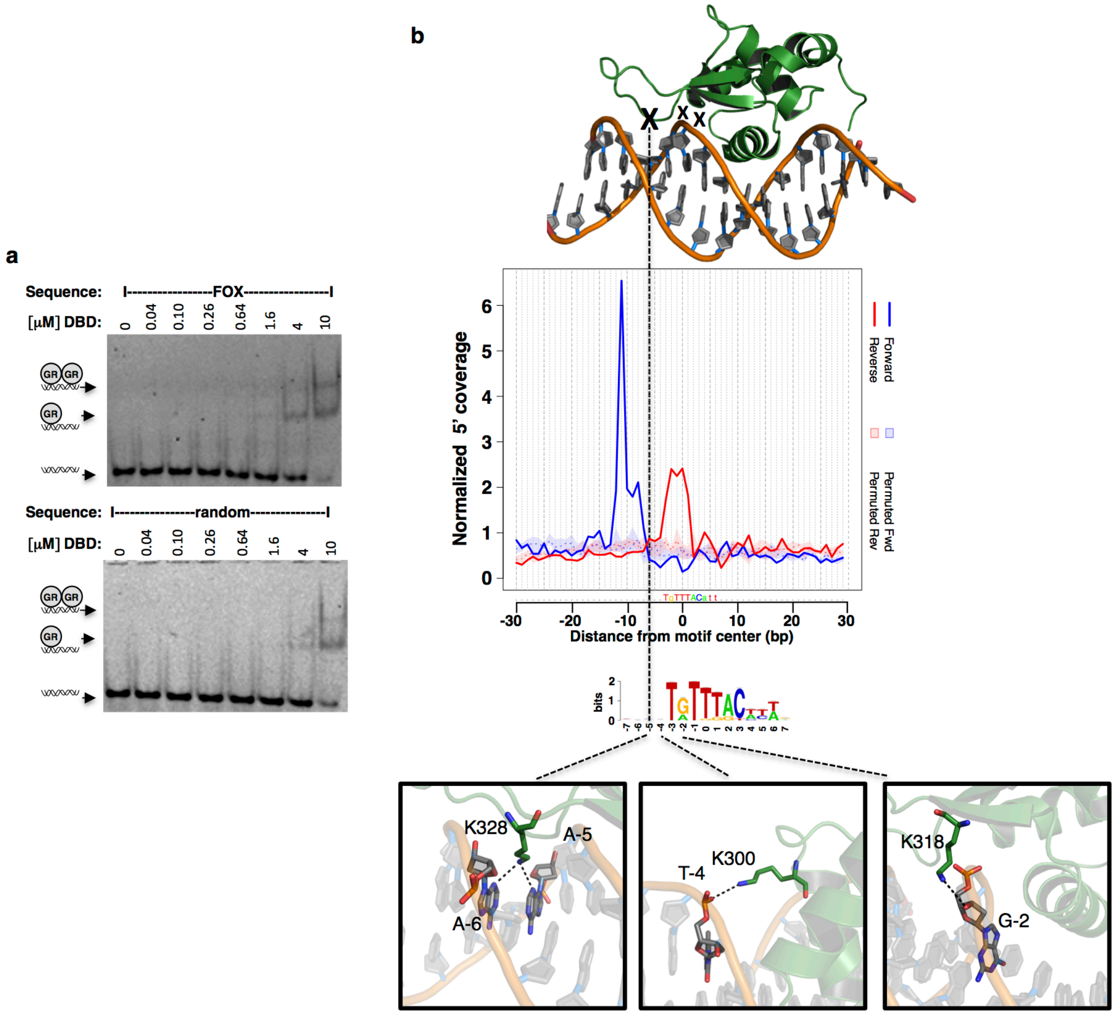

Figure S5. Structural and functional analysis of the FOX footprint profile. (a) EMSA comparing GR-DBD binding to a sequence matching (top) the FOXA1 motif and (bottom) a randomized control sequence. (b) Several potential lysine residues of FOXK1 (PDB 2C6Y) map to the proposed DNA:protein cross-link region, based of the footprint profile for FOXA1. The main proposed cross-linking point, K328, is widely conserved across members of the FOX family of transcription factors, including FOXA1.

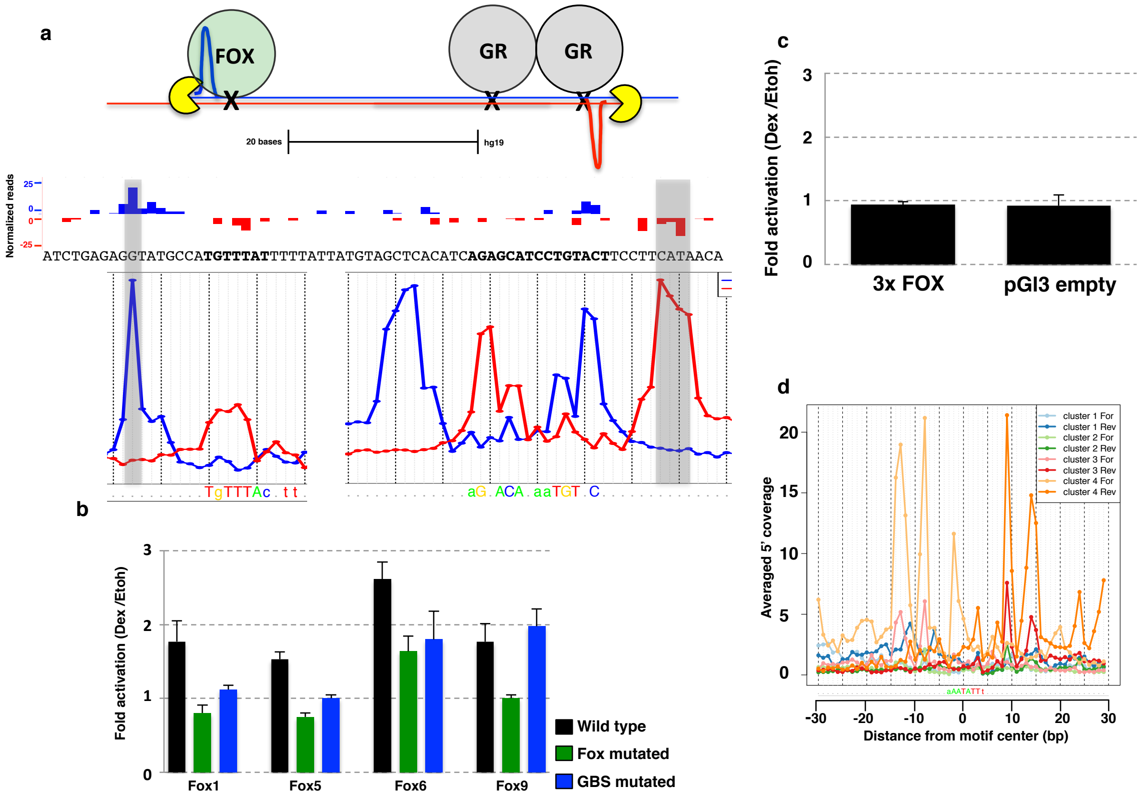

Figure S6. Functional analysis of the FOX footprint profile.(a) Correspondence between footprint profiles for FOXA1 and GBS and GR ChIP-exo reads indicate combinatorial binding of FOXA1 and GR at the *FOX6* locus (hg19: Chr15:35600909-35601308). (b) Genomic fragments near GR-target genes with sequences matching FOX motifs were cloned upstream of a minimal SV40 promoter driving luciferase expression. Fox mutated: FOX motif, TGTTTAT changed to AGCCTAT. Putative GBSs were mutated as described in the materials and methods section. Fold induction ± SEM upon treatment with 1 μM dexamethasone (dex) for wild-type and mutated reporters as indicated are shown (n≥3). (c) Transcriptional activity of empty pGL3 luciferase reporter compared to a reporter containing 3 copies of the FOX motif. Fold induction ± SEM (n≥3) in IMR90 cells upon treatment with 1 μM dexamethasone (dex) over EtOH as vehicle control is shown. (d) Average 5' coverage for each cluster as identified by K-means clustering of the 500 most occupied palindromic FOXA1 binding sites (Fig 6e).

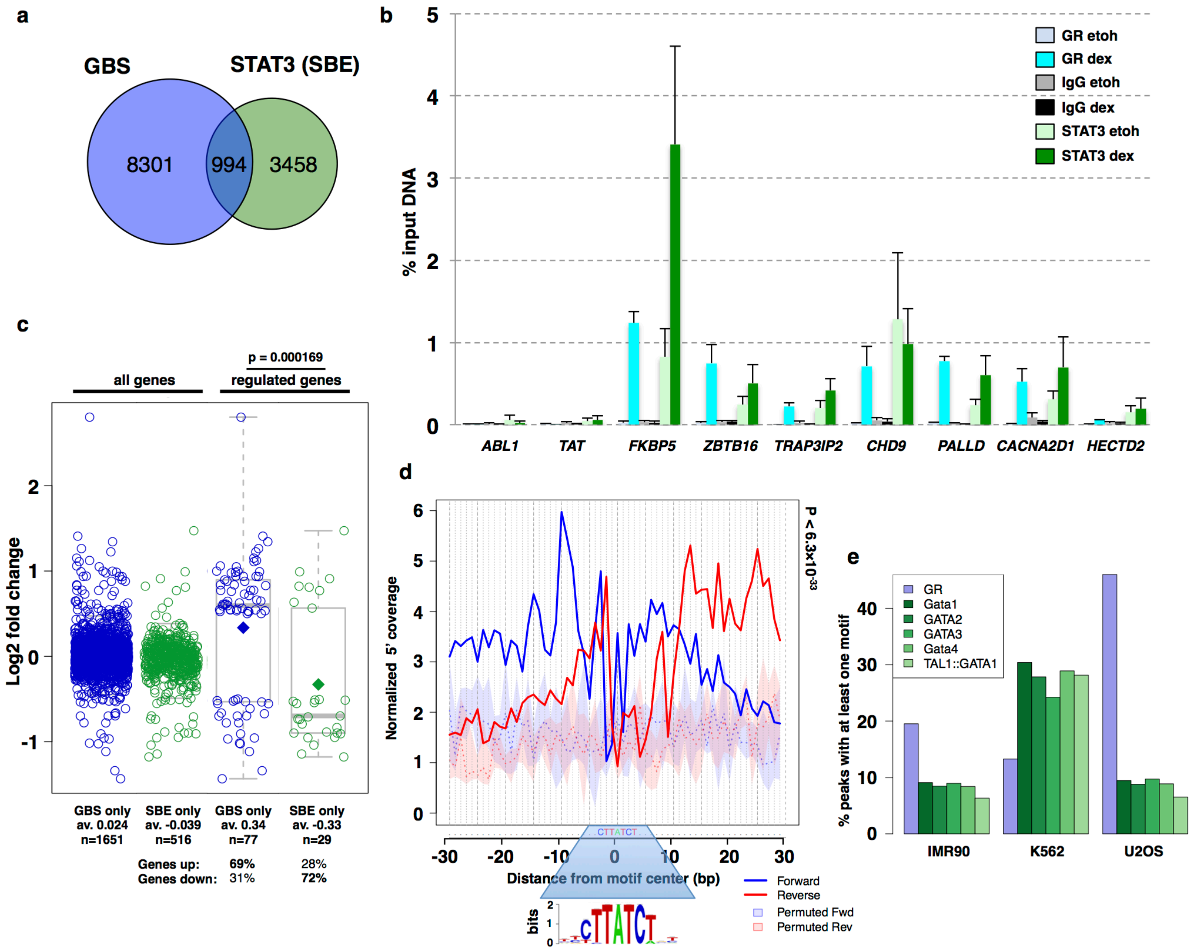

Figure S7. Analysis of non-GBS footprint profiles in GR ChIP-exo data. (a) Venn diagram showing the number of GR ChIP-seq peaks matching a STAT3 (JASPAR MA0144.2) and/or GBS motif (JASPAR MA0113.2) in IMR90 cells. (b) GR and STAT bind at the same genomic loci in IMR90 cells. ChIP experiments were performed to monitor GR, STAT3 and non-specific binding (IgG) at GR-bound and unbound control regions (*ABL1* and *TAT*) in IMR90 cells treated with EtOH as vehicle control or dexamethasone (dex). Percentage of input immunoprecipitated ± SEM (n=3) is shown. (c) Boxplot of log fold change for (left) all genes upon treatment for 4 hours with 1 µM dexamethasone and (right) for genes that are differentially expressed with a log fold change ≤ -0.5 or ≥ 0.5. Center lines show the medians; diamonds show the mean; box limits indicate the 25th and 75th percentiles as determined by R software; whiskers extend 1.5 times the interquartile range from the 25th and 75th percentiles. Genes with ChIP-seq peaks in windows ± 20 kb around the TSS, harboring a specific motif are indicated by circles (Genes with peaks with STAT3 (p<0.0001) but not GBS are marked in green, GBS (p<0.0001) but not STAT3 in blue). (d) GATA1 footprint profile (JASPAR MA0035.3) in GR ChIP-exo data from K562 cells. (e) Percentage of ChIP-seq peaks with at least one motif match (JASPAR MA0113.2, MA0035.3, MA0036.2, MA0037.2, MA0482.1, MA0140.2; p-value < 10^{-4}) is plotted for each cell line

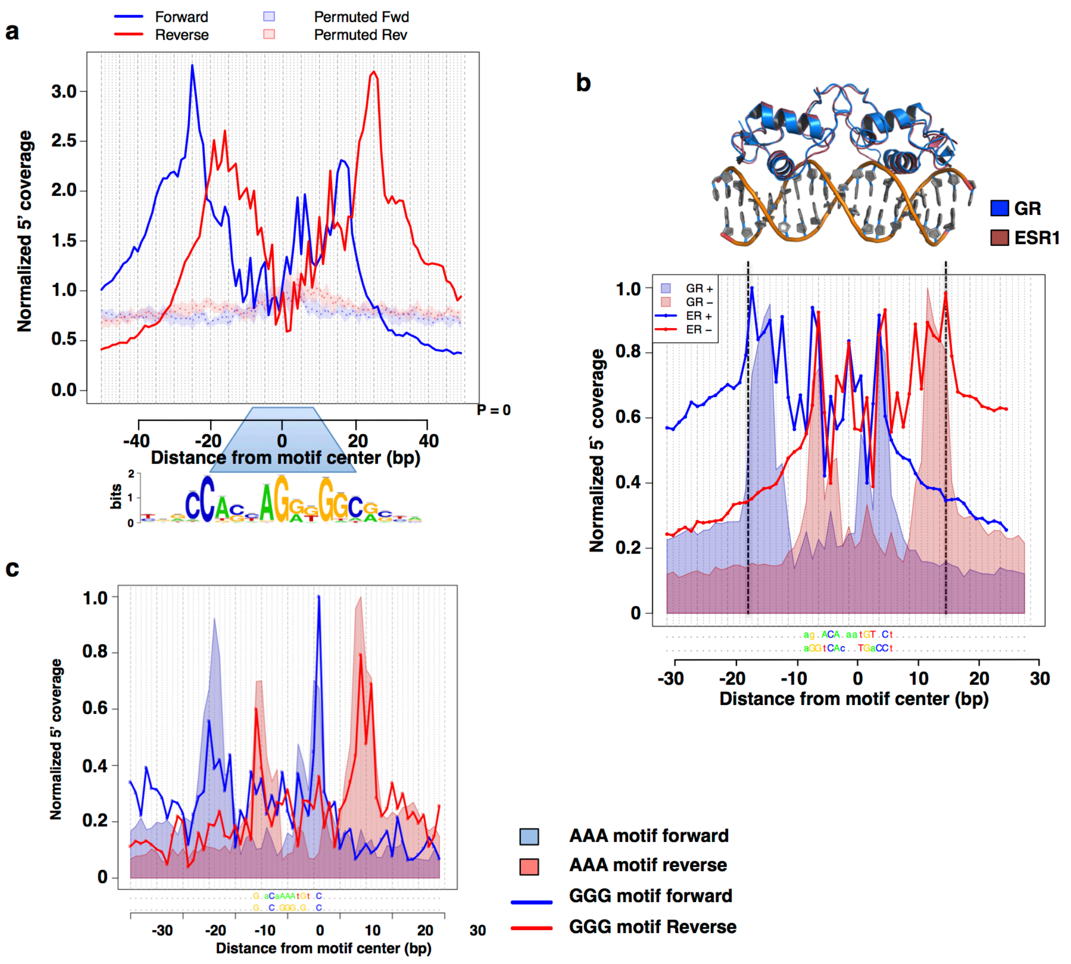

Figure S8. ExoProfiler applied to CTCF, ESR1 and GR ChIP-exo data. (a) Footprint profile for CTCF motif (MA0139.1) in CTCF ChIP-exo data. (b) top: Overlay of structures for GR:DNA (PDB 3G6U) and ESR1:DNA (PDB 1HCQ) complexes. bottom: Footprint profile overlay GBS (JASPAR MA0113.2) and ESR1 binding motif (JASPAR MA0112.2) for GR and ESR1 ChIP-exo data respectively. Dashed lines highlight comparable boundaries of protection. (c) Overlay of the footprint profile for GBSs with either AAA (matching nGnnCnAAAnGnnCn) or GGG (matching nGnnCnGGGnGnnCn) as spacer.

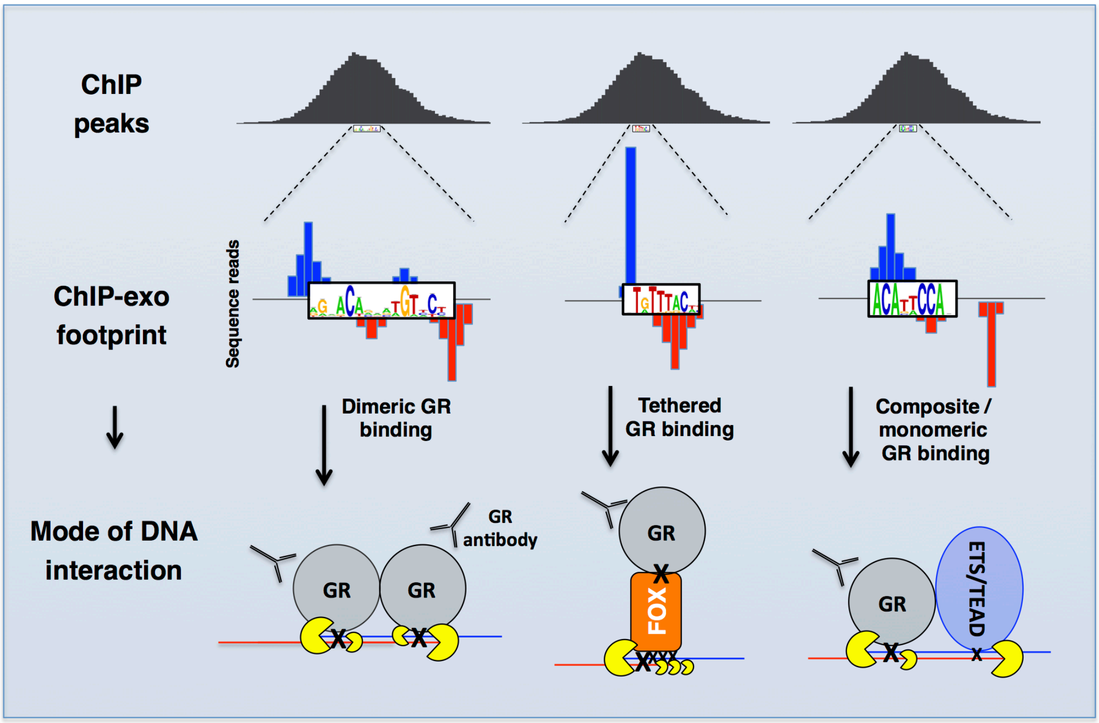

Figure S9. Cartoon depicting the increased resolution offered by the ChIP-exo procedure. ChIP-exo compared to ChIP-seq and illustration of how the shape of the ChIP-exo signal can yield clues about the diverse modes of genomic association of GR.

Supplemental Experimental Procedures

Chromatin Immunoprecipitation (ChIP), ChIP-sequencing (ChIP-seq) and ChIP-exo

ChIP and ChIP-seq assays were essentially done as described (Meijsing et al. 2009). In short: Cells were treated with 0.1% ethanol vehicle or 1 μM dexamethasone for 1 hour. Cells were cross-linked by adding formaldehyde to a final concentration of 1% and subsequent incubation 3 minutes at room temperature before quenching the reaction by adding glycine to a final concentration of 125 mM. Chromatin was sheared with a Bioruptor water bath sonicator (Diagenode) to produce fragments of ~200-500 bp. Protein G-coupled magnetic beads (dynabeads, Invitrogen) were preincubated for one hour with GR-antibody (N499) before chromatin was added and incubated for an additional 2-4 h while rotating at 4°C. Subsequently, beads were washed 4 times with 10 mM Tris-HCl pH 8.0, 1 mM EDTA, 500 mM NaCl, 5% Glycerol, 0.1% Sodium deoxycholate, 0.1% SDS, 1% Triton X-100, 0.5 mg/μl BSA followed by 4 additional washes with 20 mM Tris, pH 8.0, 1 mM EDTA, 250 mM LiCl, 0.5% NP-40, 0.5% sodium deoxycholate. ChIP-seq libraries were prepared from 10 ng of ChIP DNA. For ChIPs with STAT3 antibodies (combination of SC-482X and SC-7993X from Santa Cruz Biotechnologies) or IgG (kch-504-250, Diagenode) the ChIP procedure was identical, except that beads were washed only 4 times with RIPA buffer. For ChIPs with ETS2 (Abcam ab103478), TEAD3 (Abcam ab75192), TEAD4 (Abcam ab50945) or IgG (kch-504-250, Diagenode) as control, ChIP procedures were identical except for the washing step which was done according to (Braunstein et al. 1993). In short: 2 washes with Low Salt Immune complex wash buffer (20mM Tris-HCl pH 8.1, 2 mM EDTA, 1% Triton X-100, 0.1% SDS, 150mM NaCl) followed by 2 washes with High Salt Immune Complex Wash Buffer (same as Low Salt except for having 500mM NaCl) and 2 final washes with LiCl Immune Complex Wash Buffer (10mM Tris-HCl pH 8.1, 1 mM EDTA, 1% deoxycholic acid, 1% IGEPAL CA-630, 0.25M LiCl).

For ChIP-exo experiments, approximately 15 million cells were treated with dexamethasone and chromatin was sheared essentially as described for ChIP-seq experiment. The resulting sheared and cross-linked chromatin along with GR-antibody (N499) was sent to the Peconic company (PA, USA) for further processing.

Quantitative Real Time PCR (qPCR)

RNA isolation, reverse transcription, qPCR and data analysis were performed as described previously (Meijsing et al. 2009). Primer pairs used are listed in Table 1.

Table 1: qPCR primers used in this study

Gene/Locus:	Primer fw:	Primer rev:
ELK1 (cDNA)	TGGCCAAGGAAGAATCACAC	TTGGCAGACAAAGGAATGGC
ETS1 (cDNA)	TGCAGGTGCCTTAATGAAGC	TCACACACACACCTTTTGCC
ETS2 (cDNA)	AAAGTGGCCAAGAAGCAGTG	AATTAGCTGTGCCGTTGCTG
TEAD1 (cDNA)	TCCACCAAAGTTTGCTCCTT	GCCATTCTCAAACCTTGCAT
TEAD2 (cDNA)	TTTTGGTCTGGAGGATCTGG	ATGGGGGAGTCAGTGACAAG

TEAD3 (cDNA)	CATCCACAAGCTGAAGCAC	AGCAATGACAAGCAGGGTCT
TEAD4 (cDNA)	TCATCCACAAGCTCAAGCAC	AATGCACAGCAAGGTCTCCT
FOXO3 (cDNA)	TGCTAAGCAGGCCTCATCTC	AGAGCAGATTTGGCAAAGGG
TRNP1 (cDNA)	GCTGGAAGGACTACGGATCC	AGAAAGCCGAATCCAGAGGTC
NFIA (cDNA)	TCCAACTTGTCTGCCCTGATG	ACATTGGGGTGGGAAGGAATG
PTPN1 (cDNA)	TTTGGAGTCCCTGAATCACCAG	ATCAGCCAGACAGAAGGTTCC
ACPL2 (cDNA)	ACAGACCCCGTTTATGAAGCTC	TGGCGAATGAACACATGCAC
FKBP5 (cDNA)	AGGCTGCAAGACTGCAGATC	CTTGCCCATTGCTTTATTGG
HECTD2 (ChIP)	ACATAAGCCTGAGCAGACTCTG	AAGAAGAAGGGAGAGGTTGCAG
CHD9 (ChIP)	AGAGTGTTCTTGGAAGGAAGCC	TTGGCAGCTCAGTTCTATGC
RPL19 (ChIP & CDNA)	ATGTATCACAGCCTGTACCTG	TTCTTGGTCTCTTCCTCCTTG
FOXO3 (ChIP)	TTCAAGCTCTTCCACAGCTG	AGGTTTGGCTGTGAGGAATG
TRNP1(ChIP)	ATCCCAGCCAAGGACAAAGG	TTGTGGAGAGAAGATGCAGGAG
NFIA (ChIP)	TCTTGACCTTTCTGTCCACCAG	ATGTTCTGTTCCCAGCTGTG
PTPN1 (ChIP)	TGGTGGTGATGTTTGAGCTG	GCCCTTTGCACAAACTGTTC
ACPL2 (ChIP)	GGGATAGAACATTCCACAGTAGGG	TGCCCACGCACAAAAATGTG

Electrophoretic Mobility Shift Assays (EMSAs) with and without formaldehyde cross-linking

EMSAs were performed as described (Thomas-Chollier et al. 2013). Sequences of the 5' Cy-5 end-labeled oligos are as listed below (recognition sequence underlined):

combi	Cy5-GATCTCGAAAAGAACATTCCAGTACCTAT
combi no TTCC	Cy5-GATCTCGAAAAGAACAAACCAGTACCTAT
GBS (pal)	Cy5-TCGAAGAACAAAATGTTCTTCGA
FOX	Cy5-GATCTCGAAATAAACAAAATA
random	Cy5-TCGATACCAAAATATTTGAGTAC

A modified version of the EMSA assay described above was used to determine the efficiency of *in vitro* cross-linking of wild type and mutant versions of the GR DBD. Modifications were as follows: No BSA was present in the reaction mixes and NaCl concentration was 100 mM. Further, after the reaction mixtures reached equilibrium, formaldehyde was added to a final concentration of 0.1%, before samples were incubated for an additional 10 minutes after which formaldehyde was quenched by adding glycine to a final concentration of 125 mM. Following a 5 minute incubation, samples were loaded onto pre-run denaturing gels containing 0.1% SDS. Purification of hGR-DBD (385-540) and mutant versions R510A and K514A was done essentially as described (Meijsing et al. 2009). Oligos used for theses assays were as follows (GBS underlined):

GBS (8 bp flank)	Cy5-GATCTCGAAGAACAAAATGTTCTGTACCTAT
Random (8 bp flank)	Cy5-GATCTCGATACCAAAATATTTGAGTACCTAT

Plasmids

Reporter plasmids with genomic fragments (approx. 400 bp centered around the summit of the ChIP-seq peak) containing a sequence matching the recognition sequence for motifs of interest that are near the TSS of GR-target genes in IMR90 were amplified by PCR and cloned into the pGL3-promoter plasmid (Promega). Genomic coordinates (hg19) *Fox1:* Chr14:71323855-71324254; *Fox5*: Chr10:228340-228739; *Fox6:* Chr15:35600909-35601308; *Fox9*: Chr17:67588999-67589398; *combi-1*: Chr6:108975209-108975621; *combi-2*:

Chr1:27325909-27326321; *combi-3*: Chr1:61647749-61648161; *combi-4*: Chr20:49039019-49039431; *combi-5*: Chr3:140995189-140995601. Candidate GBS, combi or Fox sequences of these reporters were disrupted by site directed mutagenesis. Fox sequence TGTTTAT was mutated to AGCCTAT for each of the reporters. To disrupt putative GBSs of the reporters, the underlined bases of candidate GBSs were changed to an A: *Fox1*: CAGACGTACTGTTCC , *Fox5*: AGAGCATCCTGTACT, *Fox6*: AGATAAGGAAGTACT, *Fox9*: TGCTCAAAATGTTCT. Reporter plasmids containing three copies of either the FOX consensus sequence or the combi sequence were constructed using the following oligonucleotides (FOX: CCGGGAAATAAACAAAcgcgAAATAAACAAAcgcgAAATAAACAAAA combi: CCGGGAAAGAACATTCCAgcgAAAGAACATTCCAgcgAAAGAACATTCCAA recognition sequence underlined) with overhangs to facilitate direct cloning into the Xma1 and BglII sites of pGL3-promoter. Using the same approach, we constructed mutant versions of the combi reporter by using oligonucleotides with changes in the combi sequences as indicated in Fig. 5b and Fig S4c.

Transient transfections

To analyze GR-dependent regulation of luciferase reporter plasmids, U2OS cells were transiently transfected and treated overnight with 1 μM dexamethasone, harvested and luciferase activity was measured as described (Meijsing et al. 2009). For IMR90, approximately 50.000 cells in 500 μl EMEM/10% FBS were seeded per well of a 24-well plate. The following day, cells were transfected using 2 μl Lipofectamine 2000 (Invitrogen) per well according to the manufacturer instructions. Cells were transfected with 720 ng reporter plasmid and 8 ng pCMV-Renilla. After transfection (6 h), cells were re-fed with EMEM/10% FBS containing 1 μM dexamethasone or EtOH. 16-18 hours later, cells were lysed in 100 μl lysis buffer and luciferase activity was measured as described above for U2OS cells. For all experiments, at least three biological replicates were done.

dsiRNA knockdown

For dsiRNA knockdown experiments, approximately 20.000 U2OS cells were seeded per well of a 48-well plate and transfected the following day with 25 nM dsiRNAs (IDT, sequence listed in Table 1) using Lipofectamine 2000 (Invitrogen). After transfection (6 h), cells were washed once and re-fed with DMEM/5% FBS. To analyze knock-down efficiency, RNA was isolated 48 h past dsiRNA transfection using an RNeasy kit (Qiagen) and analyzed by Quantitative Real Time PCR. To measure the effect of the knockdown on luciferase reporter activity, 24 h after dsiRNA-treatment, cells were transiently transfected with luciferase reporter plasmids, treated with hormone and luciferase activity was measured as described (Meijsing et al. 2009). To measure the effect on GR-dependent regulation of endogenous target genes, U2OS cells were treated overnight with 1 μM dexamethasone or ethanol as vehicle control 48 h after dsiRNA transfection. RNA was isolated and analyzed by Quantitative Real Time PCR.

Table 2: dsiRNAs used in this study

Gene:	Duplex Name (IDT):	Target Sequence:
ELK1	HSC.RNAI.N001114123.12.1	AGGAGAAACAUAGUUCAACUGAAAG
ETS1	HSC.RNAI.N001143820.12.1	AGCAUAGAGAGCUACGAUAGUUGUG
ETS2	HSC.RNAI.N005239.12.7	CCAUGUCUUUCAAGGAUUACAUCCA
TEAD1	HSC.RNAI.N021961.12.3	ACCAGAGAAAUAUAUGAUGAACAGU
TEAD2	HSC.RNAI.N003598.12.1	CCGGCAGAUCUACGACAAAUUCCCU
TEAD3	HSC.RNAI.N003214.12.1	GGUCCUCACUGUUUGCAUAUCGCUC
TEAD4	HSC.RNAI.N003213.12.1	GCCGUGGACAUCCGCCAAAUCUAUG
Scramble	NC1 negative control duplex	-

BeadChip gene expression analysis.

Total RNA of vehicle control or hormone-treated (dexamethasone, 1 μM for 4h) IMR90 cells was purified using an RNeasy kit (Qiagen) or a NucleoSpin RNA kit (Macherey-Nagel). Biotin-labeled cRNA was synthesized from 500 ng total RNA for 4 biological replicates for each condition using the TotalPrep RNA amplification Kit (Ambion) according to the manufacturer's instructions. The labeled cDNA was hybridized to HumanHT-12 v3 BeadChip (Illumina). Following washing and staining, the BeadChip were scanned using the Illumina BeadStation 500. Pre-processing and differential expression analysis were done in R using the beadarray package (Dunning et al. 2007), using the "summarize" and "normaliseIllumina" functions and the quantile normalization method. Differentially expressed genes among different samples were identified based on the moderated t-test implemented in the limma package (Smyth 2004). The data were deposited in ArrayExpress (accession number E-MTAB-2954).

Computational analyses

ChIP-seq processing

The quality of the obtained reads was checked with FASTQC (http://www.bioinformatics.babraham.ac.uk/projects/fastqc/). They were then mapped with Bowtie 1 (-v 2 -m 1) (Langmead et al. 2009) on the human GRCh37/hg19 assembly. The peak-calling step was performed with MACS 1.4 (--bw 300, --mfold 10,30 –pvalue 1e-5) (Zhang et al. 2008) using as control the input DNA from each respective cell line. MACS was also run on the input DNA alone; the resulting peaks served as filters to remove artifactual peaks. A stringent cutoff of FDR 0.2 was applied before processing the peak list with PeakSplitter (Salmon-Divon et al. 2010) to subdivide the peak regions into individual enriched regions. The NSC and RSC scores were all above the thresholds defined by ENCODE consortium (NSC > 1.1 and RSC > 0.8). ChIP-seq data were deposited in ArrayExpress and ENA (accession number E-MTAB-2955). Peaks of the publicly available datasets were directly downloaded from GEO (*www.ncbi.nlm.nih.gov/**geo**/)* (CTCF: GSM325897 (Cuddapah et al. 2009), ESR1: GSM365926 (Welboren et al. 2009), FOXA1: GSM798437 (Ross-Innes et al. 2012)), or treated as above-mentioned after downloading from ArrayExpress (GR in U2OS: E-MTAB-2731). hg18 assembly peak coordinates were converted to hg19 using UCSC liftOver.

ChIP-exo processing
The uniquely mapped reads with BWA were directly obtained from the Peconic company as BAM files. Reads for the publicly available datasets were downloaded from ENA (*www.ebi.ac.uk/**ena/**)* (CTCF: SRA accession SRA044886, replicate 3 SRR346403 (Rhee and Pugh 2011), ESR1: ERP003828 (Serandour et al. 2013), FOXA1: ERR336963 (Serandour et al. 2013). ESR1 and FOXA1 reads were aligned with Bowtie 1 retaining only uniquely mapped reads (-m 1 –v 2). The CTCF dataset was processed similarly to the original study (Rhee and Pugh 2011): the reads were mapped with Bowtie 1 in colorspace, retaining uniquely mapped reads (-v 3 –m 1). Unmapped reads were trimmed by 6 bp from 3' end and were mapped again. ChIP-seq and ChIP-exo data were deposited in ArrayExpress and ENA (accession number E-MTAB-2956).

Fraction of ChIP-seq peaks with GBS
GR ChIP-seq peak sequences (+/-50 bp around the peak summit) were scanned with the JASPAR motif MA0113.2 (Mathelier et al. 2014) for GR, using the program RSAT *matrix-scan* (Turatsinze et al. 2008; Thomas-Chollier et al. 2011). The background model trained for each cell line on the corresponding peak sequences is a Markov chain of order 1, which accounts for the CpG depletion of vertebrate genomes. To determine which sequence segments are considered as match, we set the threshold on the p-value associated to the weight score. This threshold ranged from 10^{-6} (very stringent) to 10^{-1} (very loose). As control sequences, the coordinates of GR peaks from all cell lines were randomly shifted into the regions flanking the actual peaks. The flanking regions were defined as 2 kb on each side of the peak after extending the peaks by 200 bp on both sides. This was achieved with slop, flank and shuffle from the BEDTools suite (Quinlan and Hall 2010). As above, the background model was trained on this dataset.

ExoProfiler pipeline
To analyze the local 5' coverage distribution centered on TFBSs, we developed a computational pipeline called ExoProfiler (Fig. 1b), implemented in Python and R. This pipeline is composed of three tools aiming at scanning sequences for TFBS (matrixScanWS.py), performing profile computation (fivePrimeCounter), and finally plotting the computed footprint profiles (exoPlotter.R).

TFBS predictions (matrixScanWS.py):
First, a TFBS coordinates BED file must be obtained from a motif-scanning program. The motifs used for scanning (using *matrix-scan*) were obtained from a collection of reference motifs (JASPAR November 2013 (Mathelier et al. 2014), vertebrates only, 205 motifs) and from *de novo* motifs discovered on ChIP-seq peaks sequences with RSAT *peak-motifs* (Thomas-Chollier et al. 2012a; Thomas-Chollier et al. 2012b) (default parameters, using the four algorithms, 5 motifs per algorithm), both on the complete peak length or on ±30 bp around the peak summit to better benefit from the two algorithms based on positional bias. The results shown are for ± 30 bp around the peak summit. Each motif was given as input to RSAT *matrix-scan* (Turatsinze et al. 2008; Thomas-Chollier et al. 2011), as described above, with a stringent threshold set on the weight score p-value 10^{-4}. For palindromic motifs reported at the same position on both strands, the match associated to the lowest p-value was retained. As control, each motif had

its columns randomly permuted ten times independently using RSAT *convert-matrix* (Thomas-Chollier et al. 2011), which maintain the statistical properties of the original matrix, but not its biological significance. RSAT *compare-matrices* (Thomas-Chollier et al. 2011) was finally run to ensure that on the one hand the permuted matrices are distinct (-lth Ncor 0.99), and on the other hand not too similar to the original matrix (-lth Ncor 0.4).
For the *in silico* mutated GBS consensus analysis, this TFBS prediction step was replaced by a pattern-matching approach using RSAT *dna-pattern* (Thomas-Chollier et al. 2011). The patterns were expressed with IUPAC code ; the "mutation" is achieved replacing a chosen letter (e.g. A) by "not this letter" (e.g. B coding for C or G or T).

*ChIP-exo 5' coverage (*fivePrimeCounter.py*):*
It takes as input the mapped reads from a ChIP-exo experiment (BAM format) and TFBS coordinates for a motif of interest (BED format). For each TFBS from the BED file, fivePrimeCounter defines a short region (e.g. +/-30 bp) centred on this TFBS. Within this region, the ChIP-exo coverage is computed as follows: starting from the observation that only the most 5' position of the reads is informative in ChIP-exo data, as it marks the boundary of protection from lambda exonuclease digestion provided by cross-linked proteins, fivePrimeCounter reduces all mapped ChIP-exo mapped reads to their 5'-most base position, generating a count profile for the selected region. The program is fully strand-sensitive, ensuring that forward and reverse read coverages are calculated with respect to the motif orientation: if the TFBS is located on the reverse strand, reads on the direct strand are counted as reverse and reads on the reverse strand as forward, and all counts are adjusted to the correct distance from the motif center (Fig. S1b). Optionally, the program also calculates the consensus sequence of all regions aligned by the motif midpoint, which necessitates as additional input the reference genome in FASTA format. Thanks to the python package HTSeq (Anders et al. 2014), fivePrimeCounter is computationally efficient, processing a typical dataset in a few minutes on a common desktop computer.

Plotting footprint profiles (ExoPlotter.R):
ExoPlotter first discards regions not covered by at least 5 ChIP-exo reads to offer a better visualisation. The pipeline outputs 4 plots of the short regions centered on motifs, with a companion R script:

- A color chart representation, which mainly serves to control that the motifs are correctly aligned and that the regions are not shifted by one base pair, a relatively common error when working with genomic coordinate files.
- A heatmap of the 5' coverage combining the forward (blue) and reverse (red) strand. The color intensities are log transformed after a pseudo.count of 1 is added to all 5' coverage counts.
- A similar heatmap, ordered after a hierarchical clustering of the ChIP-exo 5'coverage at individual short regions. The distance between individual sites is calculated as follows: After adding a pseudo-count of 1, each 5'coverage count *c* is log normalized by *log(c)/log(c_max))*, where *c_max*

is the maximal count for forward or reverse 5' coverage. For each individual site, the coverage count signal is then smoothed along the genomic positions using the 'smooth' function in R with default parameters. The Euclidean distances on the log-normalized and smoothed count vectors is used for hierarchical clustering.

- A footprint profile, summing the coverage at each position for all regions, for the forward (blue) and reverse (red) strand. The raw sum is plotted unless the user chooses to add the permuted motif control. In this case, the values are normalized by dividing the counts by the number of motifs matches in the assay and in each permutation. A p-value, determining the significance of the enrichment of ChIP-exo reads around the motif, is calculated using a Wilcoxon rank-sum test. It tests if the total coverage on the short region is significantly higher than on the short regions extracted when using permutated motifs.

For all these plots, there is no shift in the position of the reads.

Differences between subsampled profiles
To compare profiles between degenerated motifs (Fig. 3) or between cell lines (Fig S3), new profiles were produced with the same numbers of sites. Multiple random subsets were drawn out of all sites contributing to each profile to confirm similarity of subsampled profiles. For degenerated motifs, the difference between the full 8 constrained profile and the subsampled profile is represented as a plot over a heatmap, separating the forward and reverse strands. To calculate the difference, subsampled and full profiles were first divided by their respective maximum values for normalization, then the full profile is subtracted from the subsampled profile values.

K-means clustering of ChIP-exo footprints
Individual sites were clustered using K-means clustering with k=4 clusters and 100 restarts with the function 'kmeans' from the 'stats' package in R.
The distance between individual sites is calculated as follows: After adding a pseudo-count of 1, each 5'coverage count value *c* at binding site s is log normalized by the total counts of all positions of site s *log(c)/log(C_s)*, where *C_s* is the sum of all counts for each position in s for forward or reverse 5' coverage. For each site, the coverage count signal is then smoothed along the genomic positions using the 'smooth' function in R with default parameters. The Euclidean distances on the log-normalized and smoothed count vectors is used for K-means clustering.

Structural alignment
Structural alignments of protein and DNA complexes were obtained as follows: A structural model of a DNA hybrid sequence was generated using 3D-Dart (van Dijk and Bonvin 2009). The hybrid sequence always consisted of the GR half site AGAACA and the binding motif of the alignment partner. The latter was derived from the corresponding PDB file and comprised the JASPER consensus sequence (Mathelier et al. 2014). For instance, a hybrid sequence for the GR:ETS1-DNA complex consisted of the 5'-CAG ATT TCC GGC ACT-3' motif of the ETS1 structure (PDB entry 1K79) and the 5'-AGA ACA CCC TGT TCT-3' for GR (PBD entry 3G6U),

comprising ETS1 binding site TTCC and GR half site AGAACA. An overview of all hybrid sequences used is given in table 3. GR and potential interaction partner binding motifs were aligned using the CE-align algorithm (Jia et al. 2004) to the 3D-DART DNA model of the hybrid sequence. A complete list of structures used for alignment is provided in table 3.

Table 3

Protein	PDB	sequence of bound DNA[$]	hybrid sequence[$]
**GR**	3G6U	A AGA ACA CCC TGT TCT	-
**TEAD1**	2HZD	AAT GTC GTT T[#]	A AGA ACA TTC CTC TGC
**ETS1**	1K79	CAC ATT TCC GGC ACT	A AGA ACA TTC CGG CAC T
**ELK1**	1DUX	TGA CCG GAA GTG T	A AGA ACA TTC CGG TCA
**FOXK1**	2C6Y	TGT AAA CAA T	AAA TA TTT[§]

[$]all sequences listed are in 5'->3' orientation

[#]not bound to DNA - alignment via Mos1 (PDB 3HOS)

[§]not used for structural alignments with GR; used to show palindromic FOX site

Supplemental References

Anders S, Pyl PT, Huber W. 2014. HTSeq - A Python framework to work with high-throughput sequencing data. *Bioinformatics*.

Braunstein M, Rose AB, Holmes SG, Allis CD, Broach JR. 1993. Transcriptional silencing in yeast is associated with reduced nucleosome acetylation. *Genes & development* **7**(4): 592-604.

Cuddapah S, Jothi R, Schones DE, Roh TY, Cui K, Zhao K. 2009. Global analysis of the insulator binding protein CTCF in chromatin barrier regions reveals demarcation of active and repressive domains. *Genome research* **19**(1): 24-32.

Dunning MJ, Smith ML, Ritchie ME, Tavare S. 2007. beadarray: R classes and methods for Illumina bead-based data. *Bioinformatics* **23**(16): 2183-2184.

Jia Y, Dewey TG, Shindyalov IN, Bourne PE. 2004. A new scoring function and associated statistical significance for structure alignment by CE. *Journal of computational biology : a journal of computational molecular cell biology* **11**(5): 787-799.

Langmead B, Trapnell C, Pop M, Salzberg SL. 2009. Ultrafast and memory-efficient alignment of short DNA sequences to the human genome. *Genome biology* **10**(3): R25.

Mathelier A, Zhao X, Zhang AW, Parcy F, Worsley-Hunt R, Arenillas DJ, Buchman S, Chen CY, Chou A, Ienasescu H et al. 2014. JASPAR 2014: an extensively expanded and updated open-access database of transcription factor binding profiles. *Nucleic acids research* **42**(Database issue): D142-147.

Meijsing SH, Pufall MA, So AY, Bates DL, Chen L, Yamamoto KR. 2009. DNA binding site sequence directs glucocorticoid receptor structure and activity. *Science* **324**(5925): 407-410.

Quinlan AR, Hall IM. 2010. BEDTools: a flexible suite of utilities for comparing genomic features. *Bioinformatics* **26**(6): 841-842.

Rhee HS, Pugh BF. 2011. Comprehensive genome-wide protein-DNA interactions detected at single-nucleotide resolution. *Cell* **147**(6): 1408-1419.

Rogatsky I, Trowbridge JM, Garabedian MJ. 1997. Glucocorticoid receptor-mediated cell cycle arrest is achieved through distinct cell-specific transcriptional regulatory mechanisms. *Molecular and cellular biology* **17**(6): 3181-3193.

Ross-Innes CS, Stark R, Teschendorff AE, Holmes KA, Ali HR, Dunning MJ, Brown GD, Gojis O, Ellis IO, Green AR et al. 2012. Differential oestrogen receptor binding is associated with clinical outcome in breast cancer. *Nature* **481**(7381): 389-393.

Salmon-Divon M, Dvinge H, Tammoja K, Bertone P. 2010. PeakAnalyzer: genome-wide annotation of chromatin binding and modification loci. *BMC bioinformatics* **11**: 415.

Serandour AA, Brown GD, Cohen JD, Carroll JS. 2013. Development of an Illumina-based ChIP-exonuclease method provides insight into FoxA1-DNA binding properties. *Genome biology* **14**(12): R147.

Smyth GK. 2004. Linear models and empirical bayes methods for assessing differential expression in microarray experiments. *Statistical applications in genetics and molecular biology* **3**: Article3.

Thomas-Chollier M, Darbo E, Herrmann C, Defrance M, Thieffry D, van Helden J. 2012a. A complete workflow for the analysis of full-size ChIP-seq (and similar) data sets using peak-motifs. *Nature protocols* **7**(8): 1551-1568.

Thomas-Chollier M, Defrance M, Medina-Rivera A, Sand O, Herrmann C, Thieffry D, van Helden J. 2011. RSAT 2011: regulatory sequence analysis tools. *Nucleic acids research* **39**(Web Server issue): W86-91.

Thomas-Chollier M, Herrmann C, Defrance M, Sand O, Thieffry D, van Helden J. 2012b. RSAT peak-motifs: motif analysis in full-size ChIP-seq datasets. *Nucleic acids research* **40**(4): e31.

Thomas-Chollier M, Watson LC, Cooper SB, Pufall MA, Liu JS, Borzym K, Vingron M, Yamamoto KR, Meijsing SH. 2013. A naturally occurring insertion of a single amino acid rewires transcriptional regulation by glucocorticoid receptor isoforms. *Proceedings of the National Academy of Sciences of the United States of America* **110**(44): 17826-17831.

Turatsinze JV, Thomas-Chollier M, Defrance M, van Helden J. 2008. Using RSAT to scan genome sequences for transcription factor binding sites and cis-regulatory modules. *Nature protocols* **3**(10): 1578-1588.

van Dijk M, Bonvin AM. 2009. 3D-DART: a DNA structure modelling server. *Nucleic acids research* **37**(Web Server issue): W235-239.

Welboren WJ, van Driel MA, Janssen-Megens EM, van Heeringen SJ, Sweep FC, Span PN, Stunnenberg HG. 2009. ChIP-Seq of ERalpha and RNA polymerase II defines genes differentially responding to ligands. *The EMBO journal* **28**(10): 1418-1428.

Zhang Y, Liu T, Meyer CA, Eeckhoute J, Johnson DS, Bernstein BE, Nusbaum C, Myers RM, Brown M, Li W et al. 2008. Model-based analysis of ChIP-Seq (MACS). *Genome biology* **9**(9): R137.
