# Supplementary figures and images for: ChIP-exo signal associated with DNA-binding motifs provides insight into the genomic binding of the glucocorticoid receptor and cooperating transcription factors
Source: Genome Res. 2015 Jun;25(6):825–35. doi: 10.1101/gr.185157.114 (PMC4448679; doi:10.1101/gr.185157.114)

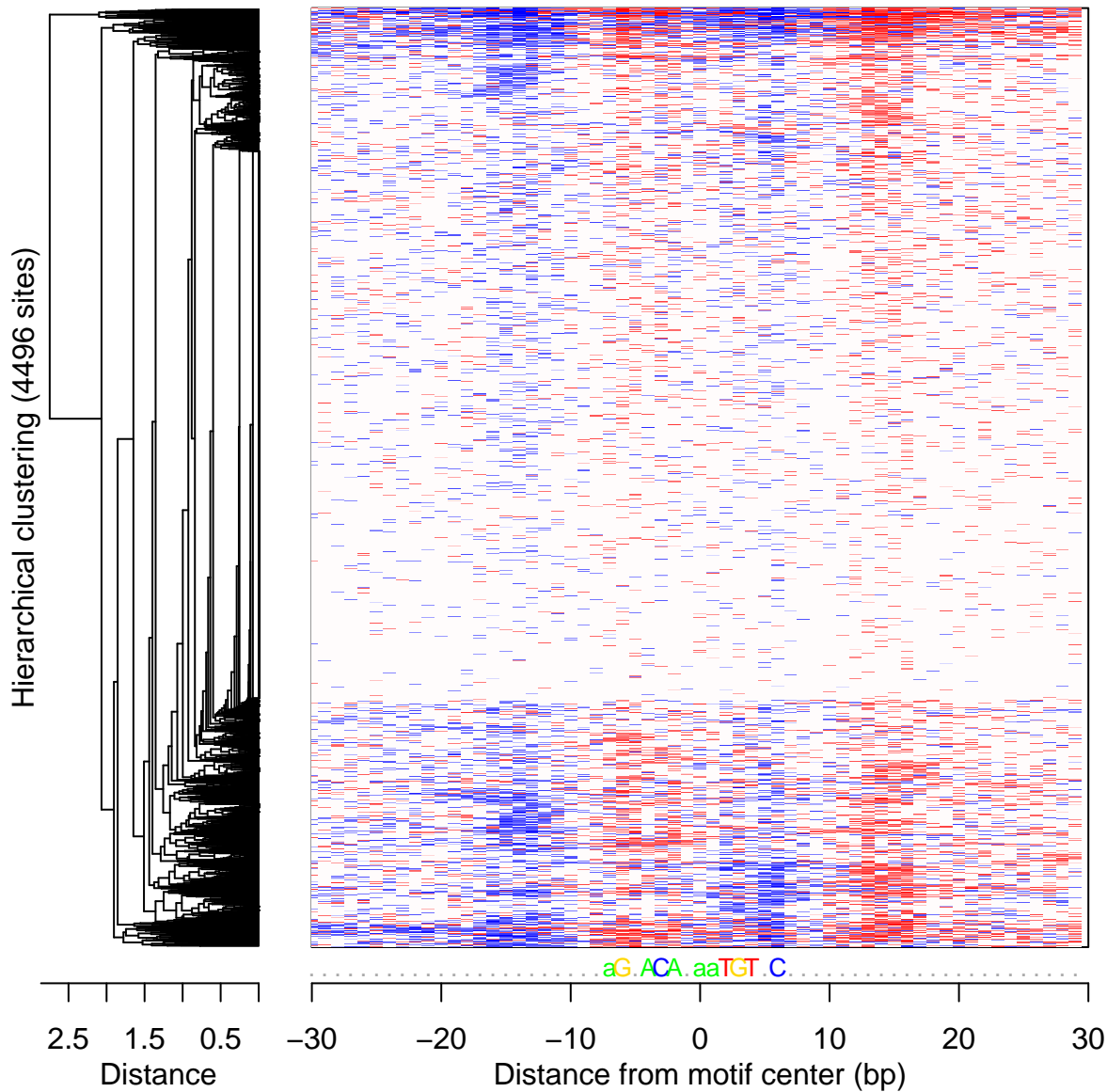

Supplement: Supplemental Material [file supp_gr.185157.114_ExoProfiler-master.zip › ExoProfiler-master/example_result/output_exoprofiler_GR-MA0113-2_0-0001.cluster.pdf]

Binding sites ( n = 4496 )

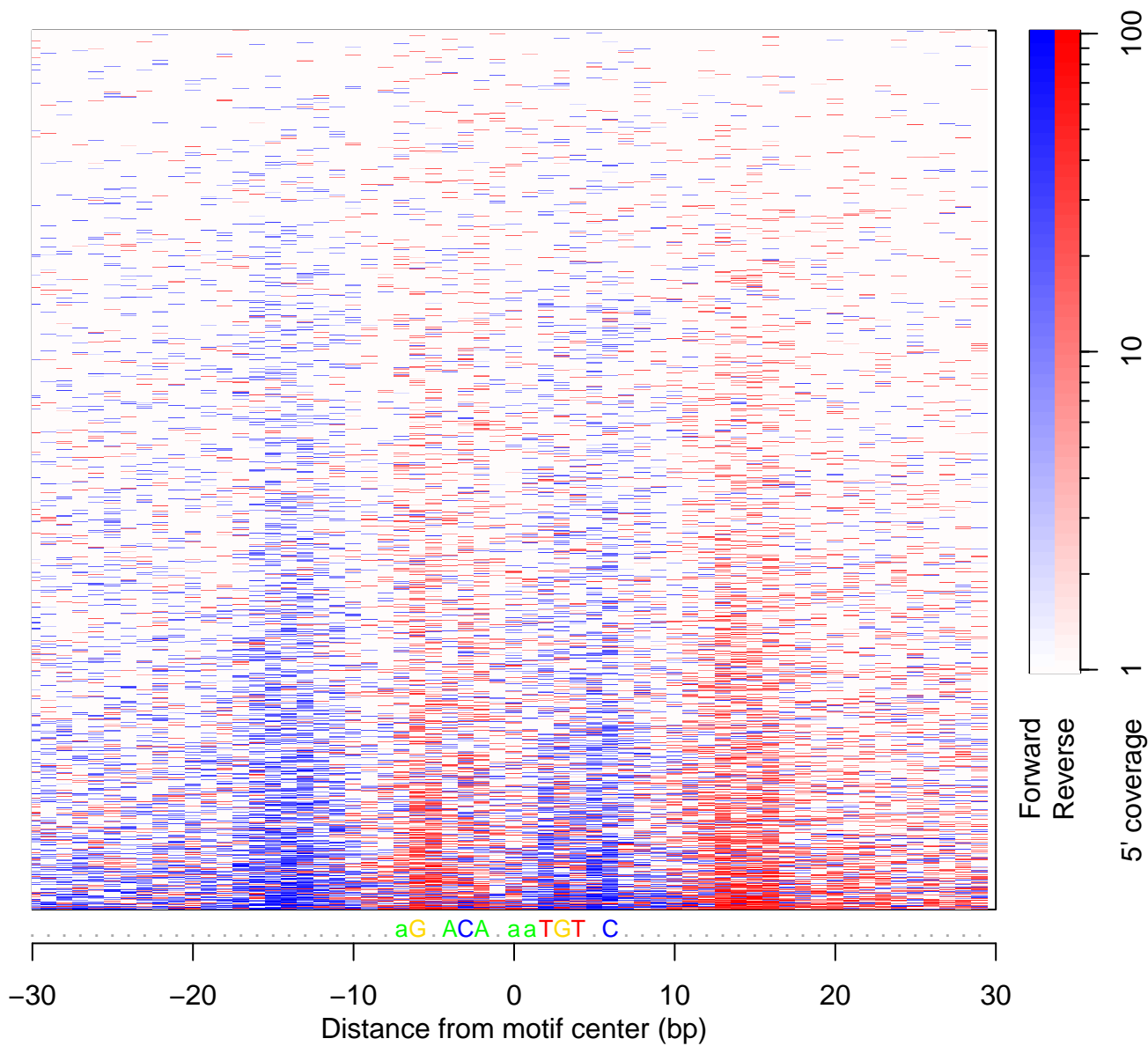

Supplement: Supplemental Material [file supp_gr.185157.114_ExoProfiler-master.zip › ExoProfiler-master/example_result/output_exoprofiler_GR-MA0113-2_0-0001.maxstrand_heatmap.pdf]

Forward      Permuted Fwd  
Reverse      Permuted Rev

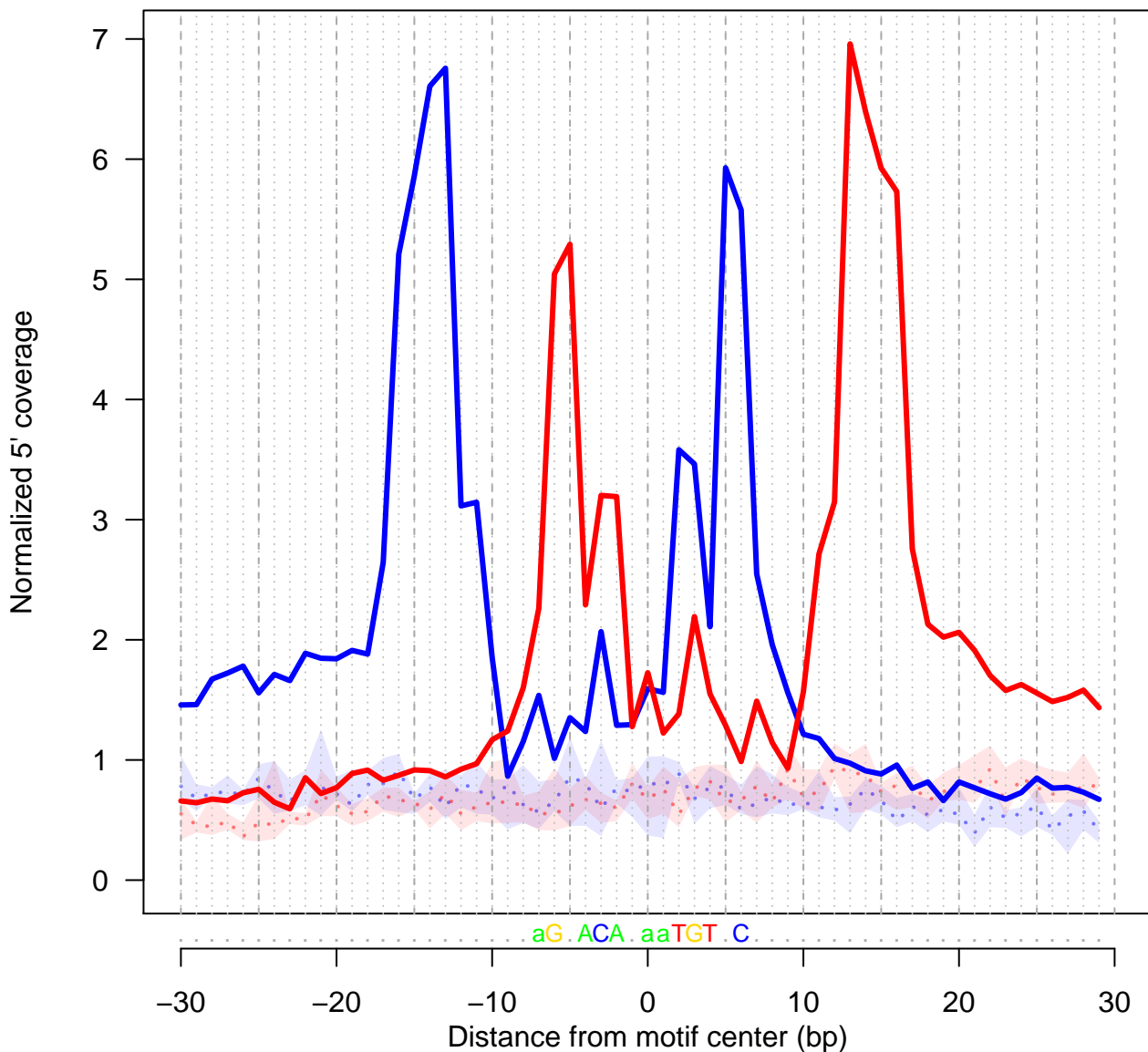

Supplement: Supplemental Material [file supp_gr.185157.114_ExoProfiler-master.zip › ExoProfiler-master/example_result/output_exoprofiler_GR-MA0113-2_0-0001.profile-perm.pdf]

Forward Reverse

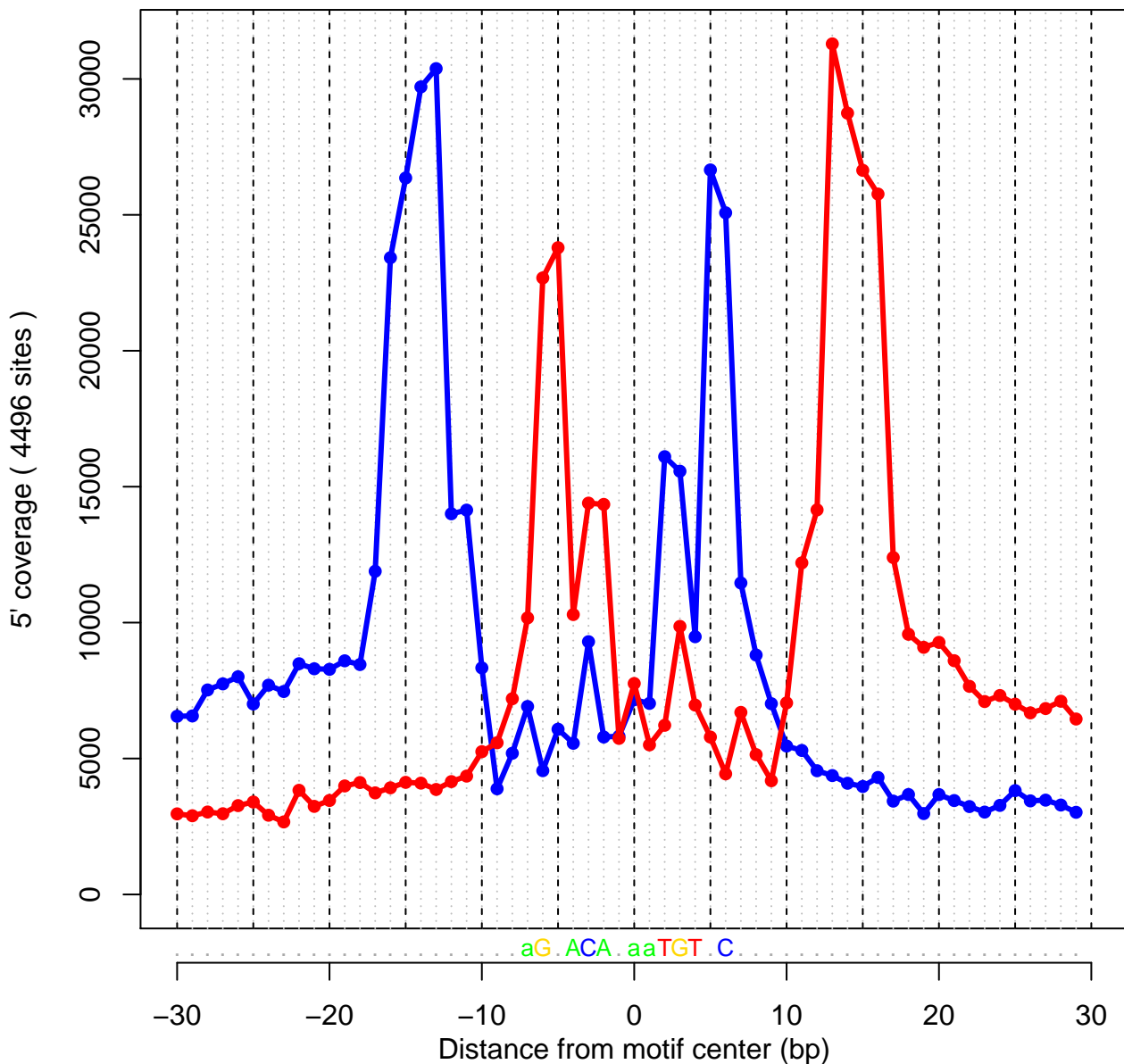

Supplement: Supplemental Material [file supp_gr.185157.114_ExoProfiler-master.zip › ExoProfiler-master/example_result/output_exoprofiler_GR-MA0113-2_0-0001.profile.pdf]

Binding sites ( n = 4496 )

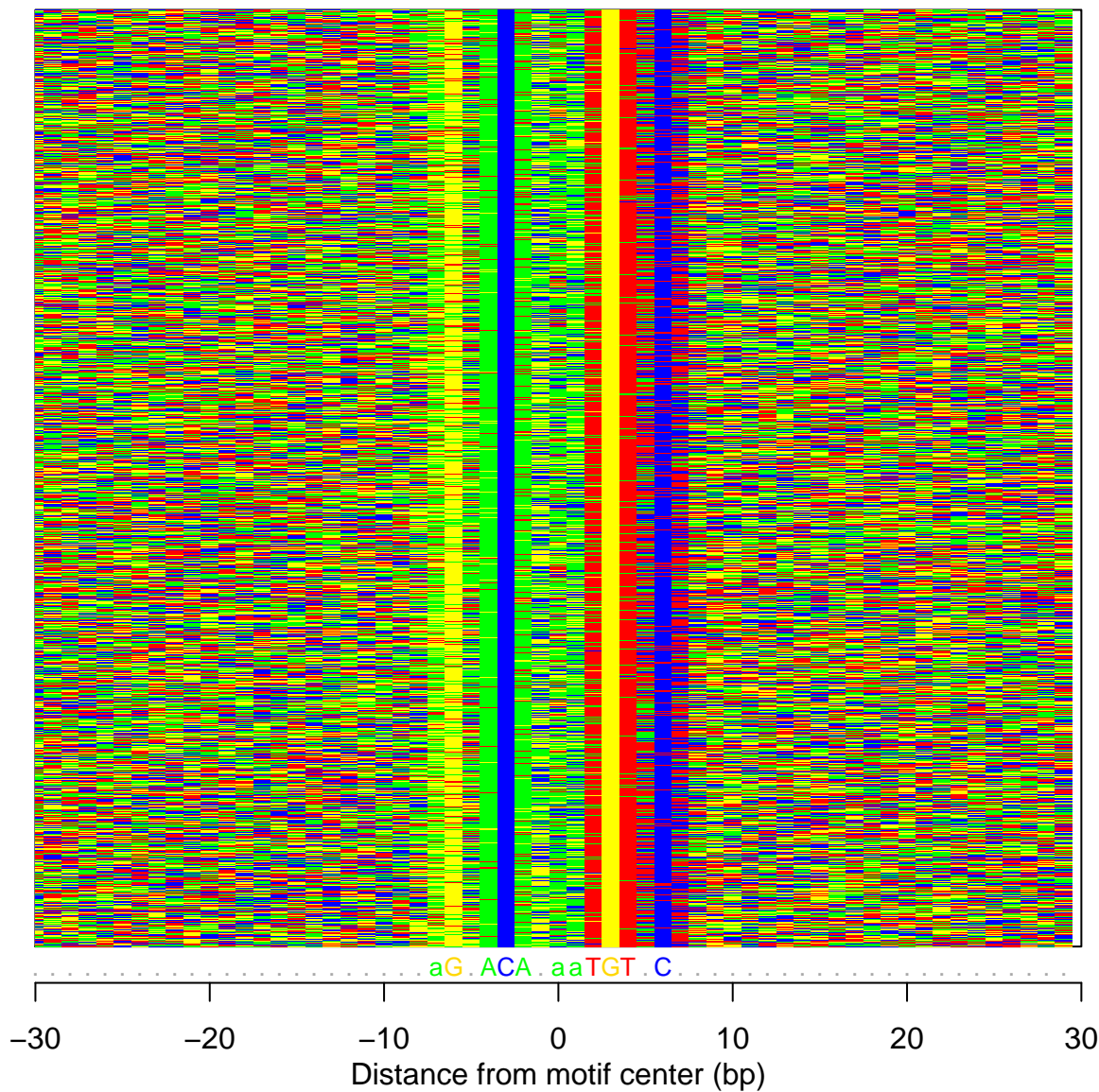

Supplement: Supplemental Material [file supp_gr.185157.114_ExoProfiler-master.zip › ExoProfiler-master/example_result/output_exoprofiler_GR-MA0113-2_0-0001_seq.pdf]
